# Supplementary material for: Hybrid integration of quantum dot single-photon sources with lithium tantalate photonics for on-chip routing
Source: Nat Commun. 2026 Jun 16;17:7638. doi: 10.1038/s41467-026-74029-5 (PMC13434732; doi:10.1038/s41467-026-74029-5)
Supplement: Supplementary file 1 [file 41467_2026_74029_MOESM1_ESM.pdf]

Supplementary Materials for

Hybrid Integration of Quantum Dot Single-Photon Sources with Lithium Tantalate Photonics for On-Chip Routing

Kaili Xiong *et al.*

*Corresponding author. Email: liujin23@mail.sysu.edu.cn; liuliuopt@zju.edu.cn; chenyan@nudt.edu.cn; tjiang@nudt.edu.cn

This PDF file includes:

Supplementary Text
Figure S1 to Figure S10
Table S1 to Table S2
References

I. Simulation and Fabrication of Thin-film Lithium Tantalate Mach-Zehnder Interferometer

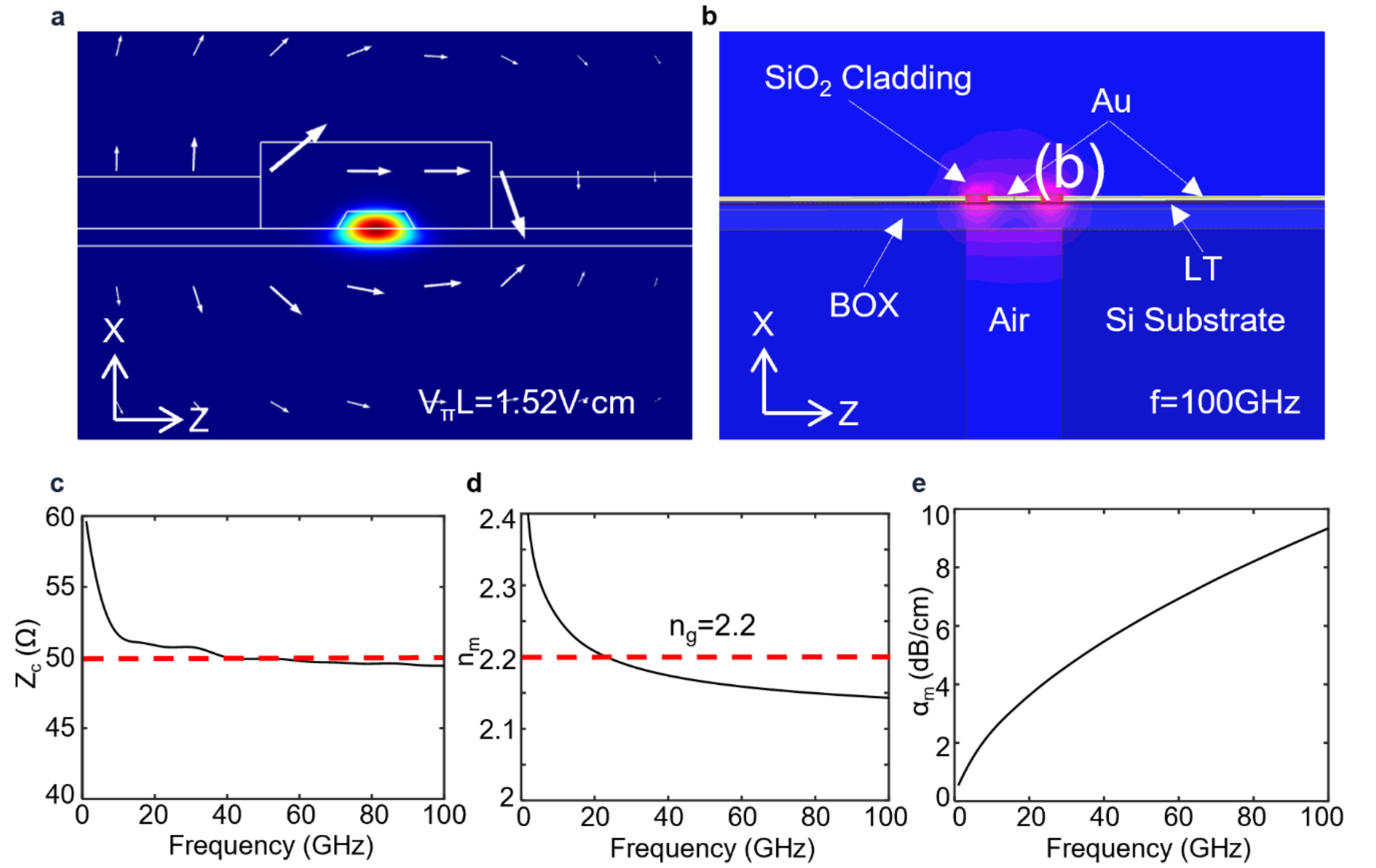

Figure S1. Optic and microwave design of the modulation section. (a) Simulated TE optical mode profile and static electric field distribution, which gives $V_\pi{\cdot}L$ =1.52 V·cm. (b) Simulated distribution of the microwave mode at 100 GHz. (c) Characteristic impedance Z_c ,(d) Microwave effective index n_m, and (e) microwave loss α_m. The two red dashed lines in (c) and (d) indicate 50 Ω and the group index n_g of the optical mode, respectively.

The designed modulator was simulated using a finite element algorithm for both electrical and optical modes of the structure. As shown in Fig. S1a, the calculated $V_\pi{\cdot}L$ for this structure is 1.52 V·cm, and the fundamental transverse-electric (TE) mode exhibits low optical propagation loss of 0.01 dB/cm due to metal absorption. Fig. S1b shows the microwave field distribution, which is localized around the optical waveguides. Through numerical simulations, the frequency-dependent parameters, specifically the characteristic impedance Z_c , the microwave effective index n_m, and the microwave loss α_m, were obtained as shown in Fig. S1c-e, respectively. The characteristic impedance Z_c remains stable at approximately 50 Ω, and the microwave effective index n_m maintains near the optical group index of n_g=2.2.

The lithium tantalate on insulator (LTOI) Mach-Zehnder modulator (MZI) was fabricated through a sequence of standard micro-fabrication processes. First, a 500 nm thick resist (ma-N2405) was patterned on an x-cut LTOI wafer using electron-beam lithography (EBL). Subsequently, 300 nm thick LT layer was etched using inductively coupled plasma reactive ion etching (ICP-RIE) with Ar plasma at an etch rate of ~80 nm/min. The chip was then covered with a 1.5 μm thick SiO_2 layer deposited via plasma enhanced chemical vapor deposition (PECVD). Ultraviolet (UV) contact lithography was employed to pattern the SiO_2 over-cladding, followed by ICP-RIE to remove the exposed SiO_2 below the electrodes. Metal electrodes composed of Au were fabricated using UV contact lithography, metal evaporation, and lift-off processes. Next, the SiO_2 over-cladding over the transfer imprinting area was selectively removed using wet etching. This ensures a smooth surface facilitating the

integration of GaAs waveguides. The silicon substrate was subsequently thinned to a final thickness of 300 µm. Hole patterns were defined on the polished backside of the chip using a double-side mask aligner, and anisotropic silicon dry etching via ICP-RIE was performed to remove the silicon substrate beneath the modulation section until reaching the BOX layer. Finally, the chip was diced and the end facets for optical coupling were polished.

II. The method of GaAs/InAs Waveguides Fabrication

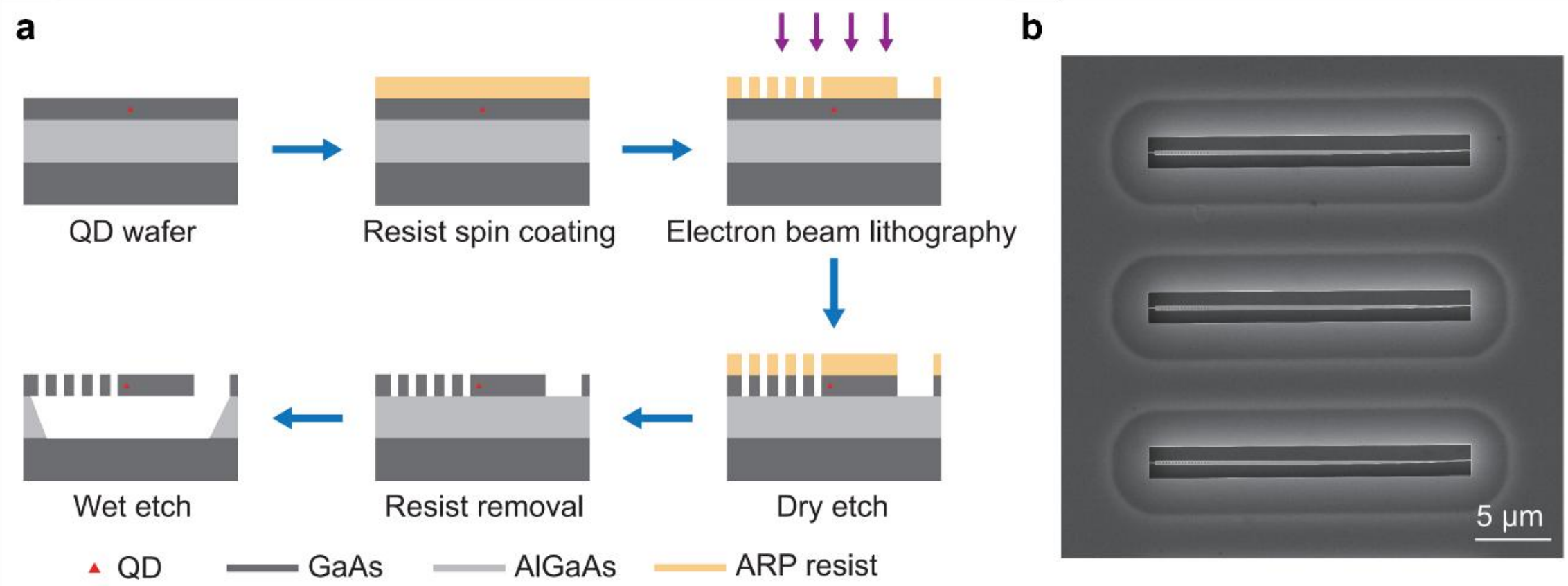

Figure S2. Fabrication of the suspended GaAs/InAs quantum dot waveguide. (a) Schematic of the fabrication process flow. (b) Scanning electron microscope (SEM) image of the fabricated waveguide.

The waveguide was processed from a wafer with embedded InAs/GaAs quantum dots grown by molecular-beam epitaxy. First, an ARP resist was spin-coated and patterned via electron-beam lithography to form a mask. Subsequently, a 200-nm-thick GaAs layer was etched through by inductively coupled plasma etching. After removal of the residual resist, the sample was immersed in a 2 % HF solution to wet-etch the AlGaAs sacrificial layer, ultimately yielding a suspended GaAs/InAs quantum dot waveguide. A scanning electron microscopy (SEM) image of the fabricated waveguide is presented in Fig. S2b.

III. High-Precision Micro-Transfer Printing of GaAs Waveguides

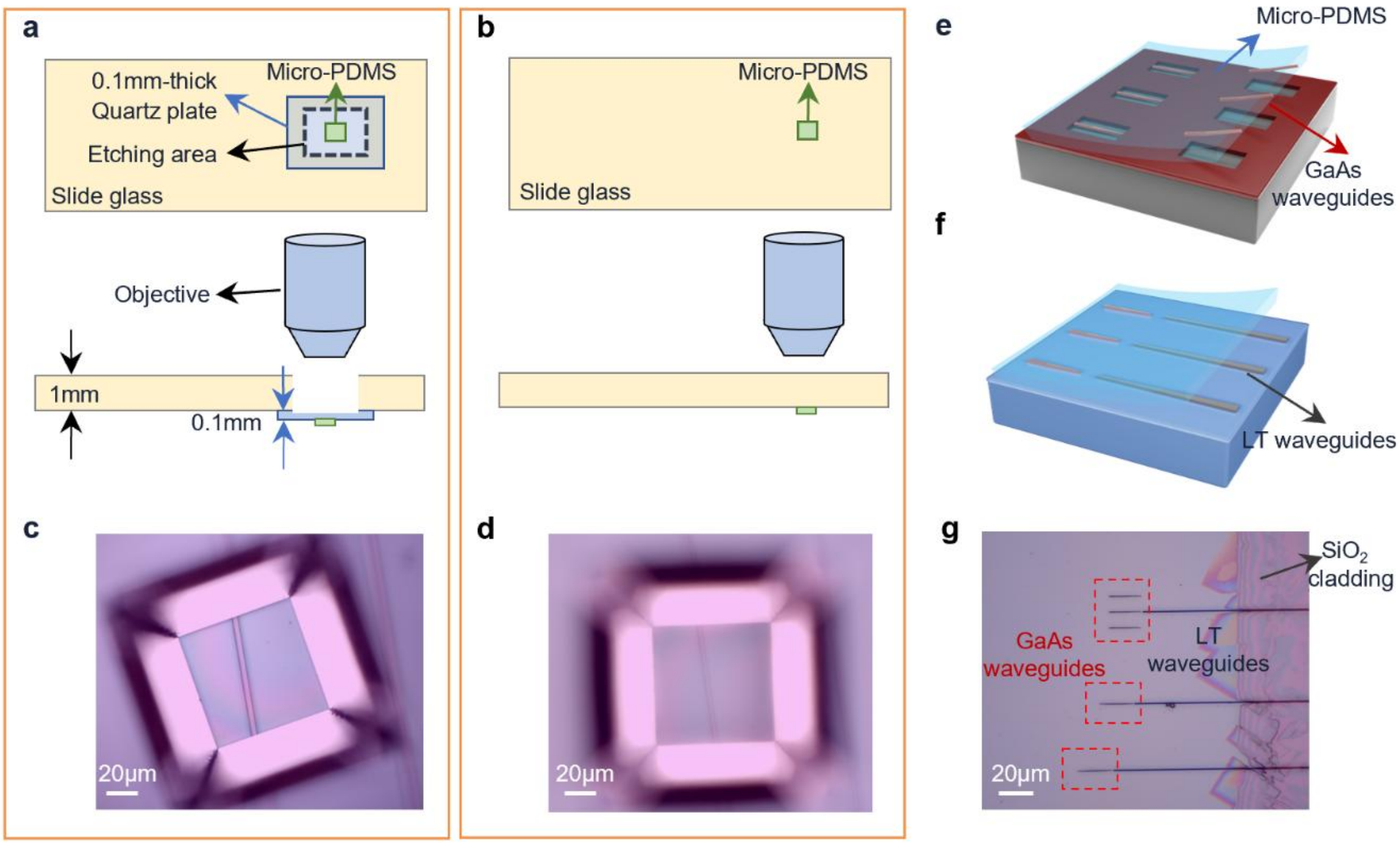

Figure S3. Modified high-precision micro-transfer printing method. (a) The front and sectional views of a modified slide. A square through-hole is laser-etched into a 1 mm-thick slide glass, over which a 0.1 mm-thick quartz plate is attached. The prepared micro-polydimethylsiloxane (PDMS) stamp is then affixed onto the quartz plate. (b) The front and sectional views of a conventional glass slide with a PDMS stamp attached. (c-d) Comparison of waveguide imaging on LT chips through modified vs. conventional slides at 50× magnification. (e) The GaAs waveguide is released via PDMS lift-off. (f) The GaAs waveguide is aligned and transfer-bonded to the LT waveguide. (g) High-precision transfer results are demonstrated in the optical micrograph.

For high-precision transfer of GaAs waveguides onto LT photonic chips, we utilized a high-resolution 3D motorized micro-transfer system with a minimum step size of 100 nm. To enhance transfer accuracy, we developed a micro-polydimethylsiloxane (PDMS) transfer printing method using 0.1 mm-thick quartz plates instead of conventional 1 mm glass slides. The modified slide is illustrated in Fig. S3a, a square through-hole is first laser-etched on a 1 mm glass slides. Then, a thin quartz plate, slightly larger than the etched area, is attached over the hole. Finally, the fabricated PDMS stamp is affixed onto the quartz surface. Fig. S3b shows a conventional glass slide with a PDMS stamp attached. LT waveguide imaging at 50× magnification (Fig. S3c-d) demonstrates significantly enhanced resolution through the modified slide owing to reduced glass thickness between objective and chip, thereby increasing optical transmittance.

During the transfer process, we first fabricated large-scale arrays of quantum dot GaAs waveguides on the wafer using electron beam lithography, chlorine-based dry etching, and selective wet etching. Next, the PDMS stamp on the modified slide was used to retrieve the GaAs waveguides from their substrate, as shown in Fig. S3e. The GaAs substrate was then replaced with the LT photonic chip. Under optical microscopy, the GaAs waveguide was aligned with the LT waveguide. By heating the PDMS to reduce its adhesion, the stamp was gently peeled off to complete the hybrid integration (Fig. S3f). Fig. S3g displays a microscopic

image of the finished LT chip with the hybrid-integrated single-photon source, confirming precise transfer spacing and angular control. As shown in the first red frame, three GaAs waveguides were simultaneously transferred by the stamp. Due to a pitch mismatch between the source array and the target LT chip, only one waveguide was coupled to the LT circuit, while the other two landed intact on unpatterned regions and remain fully functional. This simultaneous transfer demonstrates that, with optimized layout designs, our approach is highly scalable and capable of parallel, large-scale integration.

IV. Analysis of Propagation Loss in LTOI Waveguides

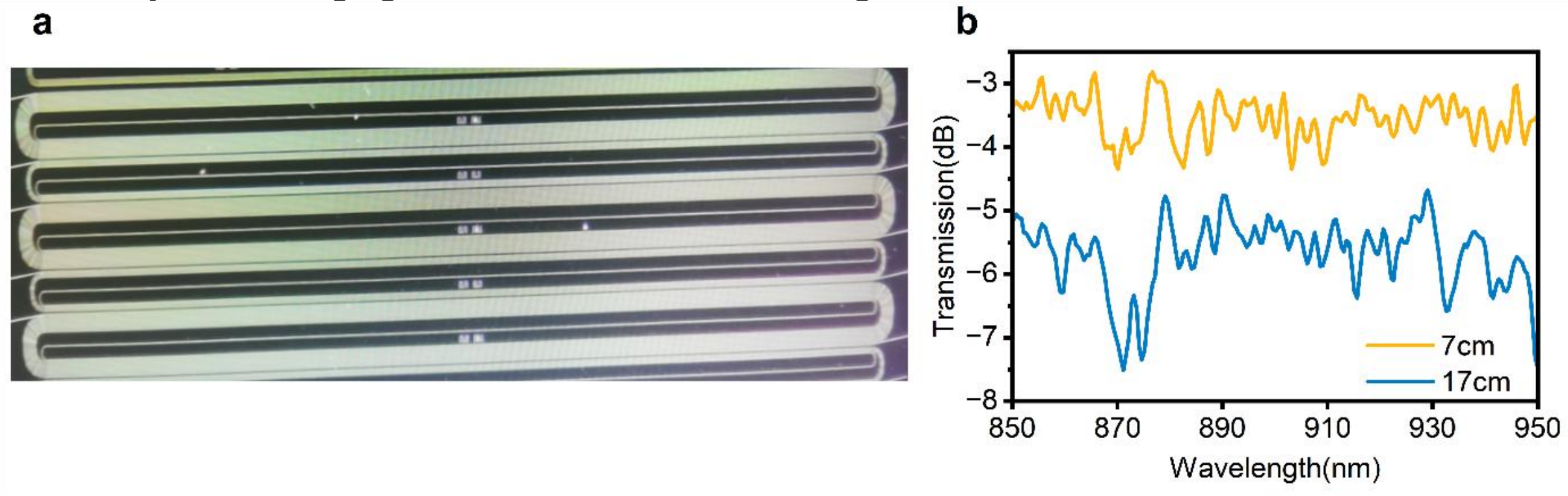

Figure S4. Measurement of the waveguide propagation loss. (a) Optical microscope picture showing a set of spiral waveguides of different lengths for testing the propagation loss of the LTOI waveguide. (b) The propagation loss of spiral waveguides with a width of 1 μm and lengths of 7 cm and 17 cm in the 850-950 nm.

To quantitatively evaluate the propagation loss of our LT waveguides, we designed and fabricated spiral waveguide structures with total lengths of 7 cm and 17 cm and a uniform width of 1 μm. These structures were fabricated using the identical process flow as the devices reported in the main text.

The transmission loss was characterized around 900-nm wavelengths using the cut-back method. The total insertion loss for each spiral includes contributions from the intrinsic waveguide propagation and the fiber-to-chip coupling losses at both input and output facets. The coupling loss is assumed to be constant for identical coupling conditions. By comparing the losses of the 7 cm and 17 cm long spirals, we extracted a propagation loss of 0.30±0.04 dB/cm.

This low loss value is a key metric indicating the high quality of our nano-fabrication process, which results in waveguides with smooth sidewalls that minimize scattering loss. The residual loss is primarily attributed to scattering from sidewall roughness and intrinsic material absorption within the LTOI. This low propagation loss is a fundamental prerequisite for achieving the high-performance modulation demonstrated in our devices.

V. Full Experimental Setup

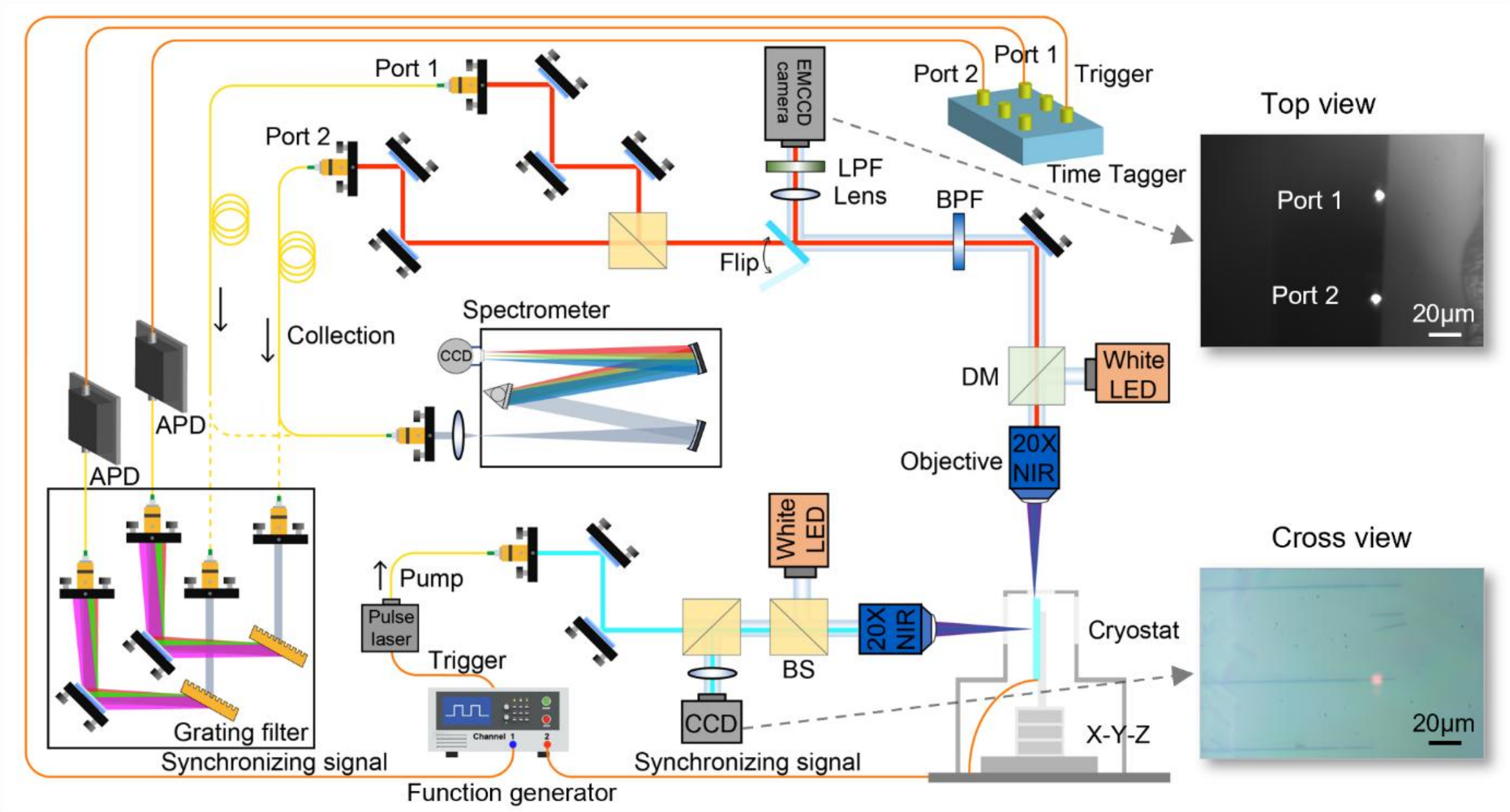

Figure S5. Schematic diagram of the side-excitation with vertical-collection optical path. The chip is vertically mounted on a 3D positioning stage within a closed-cycle helium cryostat, with optical glass windows on the side and top. The side-excitation module consists of a pulse laser, charge-coupled device (CCD), light-emitting diode (LED), beam splitter (BS), and a 20× microscope objective, among other components. The pulsed laser serves as a trigger signal output to a function generator. The synchronizing signals from channel 1 and channel 2 of the function generator are routed as follows: one path connects to an RF port on the exterior of the cryostat, with the RF signal transmitted via internal wiring to act on the chip electrodes; the other path connects to a Time Tagger for performing coincidence counting. The vertical-collection module comprises a 20× microscope objective, LED, electron-multiplying charge coupled device (EMCCD), fiber collimators, spectrometer, grating filter modules, avalanche photodiodes (APDs) and time tagger. The bottom-right cross view shows an image of the chip surface captured by the CCD. The laser is focused on the GaAs waveguide to excite quantum dots, and the photoluminescence (PL) is coupled into the LT waveguide. The upper-right panel shows a top-view image of the chip end-facet, acquired by an EMCCD. The PL signal is output from port 1 and port 2 after passing through the MZI. The spectrometer is focused on either port 1 or port 2 to analyze the PL signal of the quantum dots. The optical paths of APD1 and APD2 are focused on port 1 and port 2, respectively, to perform time-correlated measurements on the modulated single-photon signals from the modulator.

VI. Analysis of Cryogenic Modulation Depth and Residual Intensity

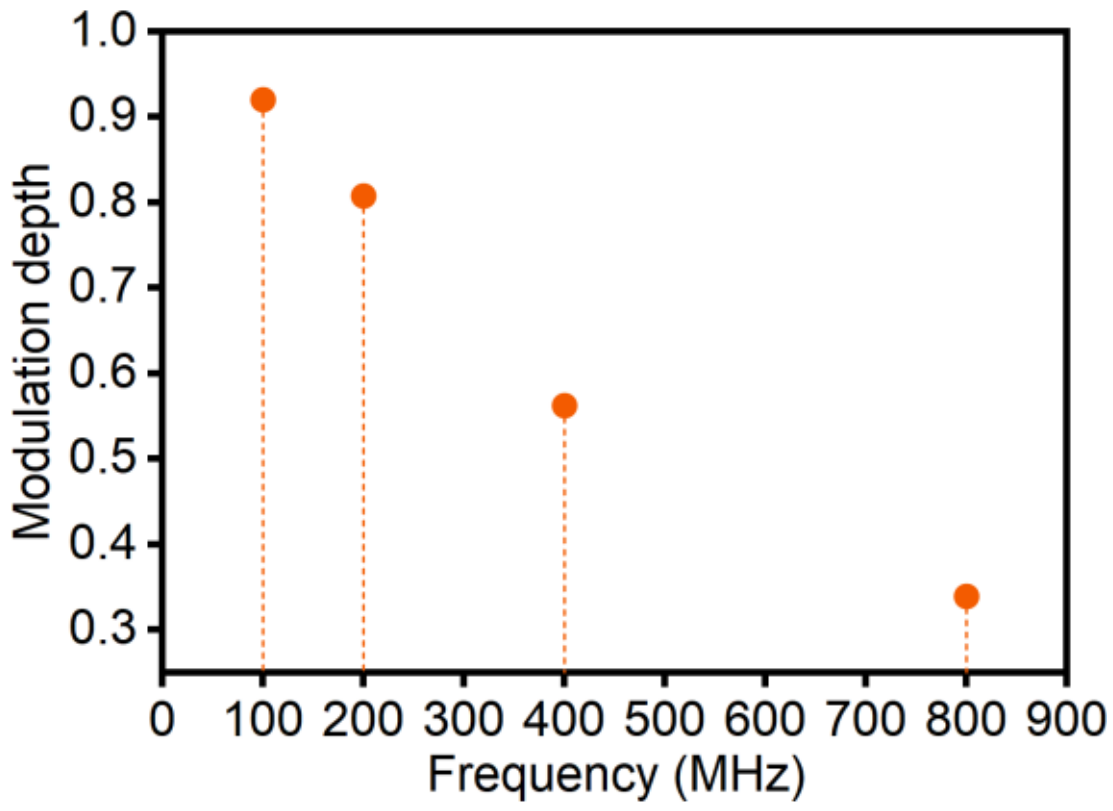

Figure S6. Measured modulation depth as a function of frequency at cryogenic temperature. The decreasing trend confirms that the modulation efficiency is limited by the RF packaging bandwidth (wire bonding and impedance mismatch).

The incomplete extinction (modulation depth of ~90%) at cryogenic temperatures is primarily attributed to the limitations of the electrical packaging used in the cryostat, specifically regarding impedance matching and wire bonding:

1) Impedance Mismatch: Unlike our room-temperature characterization where precise 50 Ω termination was applied, the cryogenic setup lacked perfect impedance matching throughout the transmission line. This mismatch causes signal reflections, preventing the effective voltage across the electrodes from fully reaching the required V_π for complete extinction.
2) Parasitic Inductance from Wire Bonding: As noted in the manuscript, the device was connected to the RF carrier via wire bonding for cryogenic operation, rather than using high-speed RF probes. The bonding wires introduce parasitic inductance, which acts as a low-pass filter and attenuates the RF signal reaching the traveling wave electrodes.

To verify that this is a packaging-induced bandwidth limitation rather than an intrinsic device defect, we performed additional measurements of the modulation depth at varying frequencies (100 MHz, 200 MHz, 400 MHz, and 800 MHz). As shown in Figure S6, the modulation depth decreases significantly as the frequency increases. This frequency-dependent roll-off confirms that the residual intensity is due to the RF attenuation caused by the wire bonds and impedance mismatch, preventing the drive signal from achieving the full amplitude required for 100% modulation depth.

VII. Analysis of Collection Efficiency

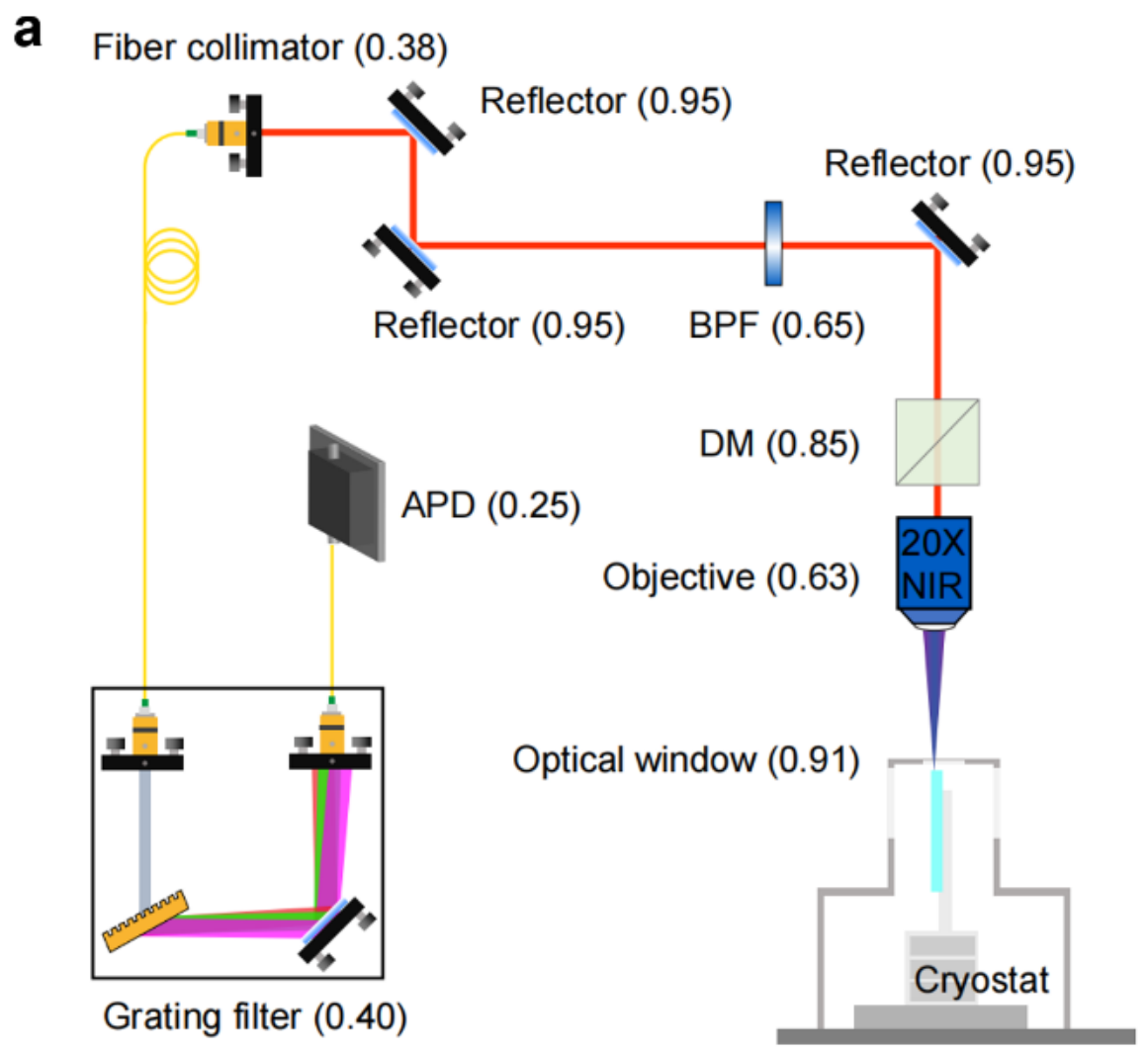

b

Optical setup	Transmission
Optical window	0.91±0.01
Objective	0.63±0.03
DM	0.85±0.02
BPF	0.65±0.02
Reflector×3	$(0.95\pm0.01)^3$
Fiber collimator	0.38±0.05
Grating filter	0.40±0.10
APD	0.25±0.02
Total setup	0.0103±0.0012

Figure S7. Calibration of optical transmission losses of the optic elements in the setup. (a) All the optical elements used in the collection path. (b) Transmissions of the optical elements in the setup with a total efficiency of 0.0103±0.0012.

To precisely evaluate the performance of our hybrid-integrated platform, we meticulously calibrated the transmission losses of the optical elements in the setup. The signal sequentially passes through an optical window, objective lens, dichroic mirror (DM), bandpass filter (BPF), reflector, fiber collimator (which includes facet loss), and grating filter before detection by an APD. The total transmission efficiency of the setup was measured at 0.0103 ± 0.0012. By employing an 80 MHz femtosecond pulsed laser for above-band excitation of the quantum dot, the saturated count rate of 0.245 ± 0.005 MHz was extracted from the power-dependent photoluminescence saturation curve in Fig. 4b. This yields a measured single-photon extraction efficiency at the first objective lens of $\eta_{exp} = \frac{0.245\pm0.005}{(0.0103\pm0.0012)\times 80} = 0.297 \pm 0.035$.

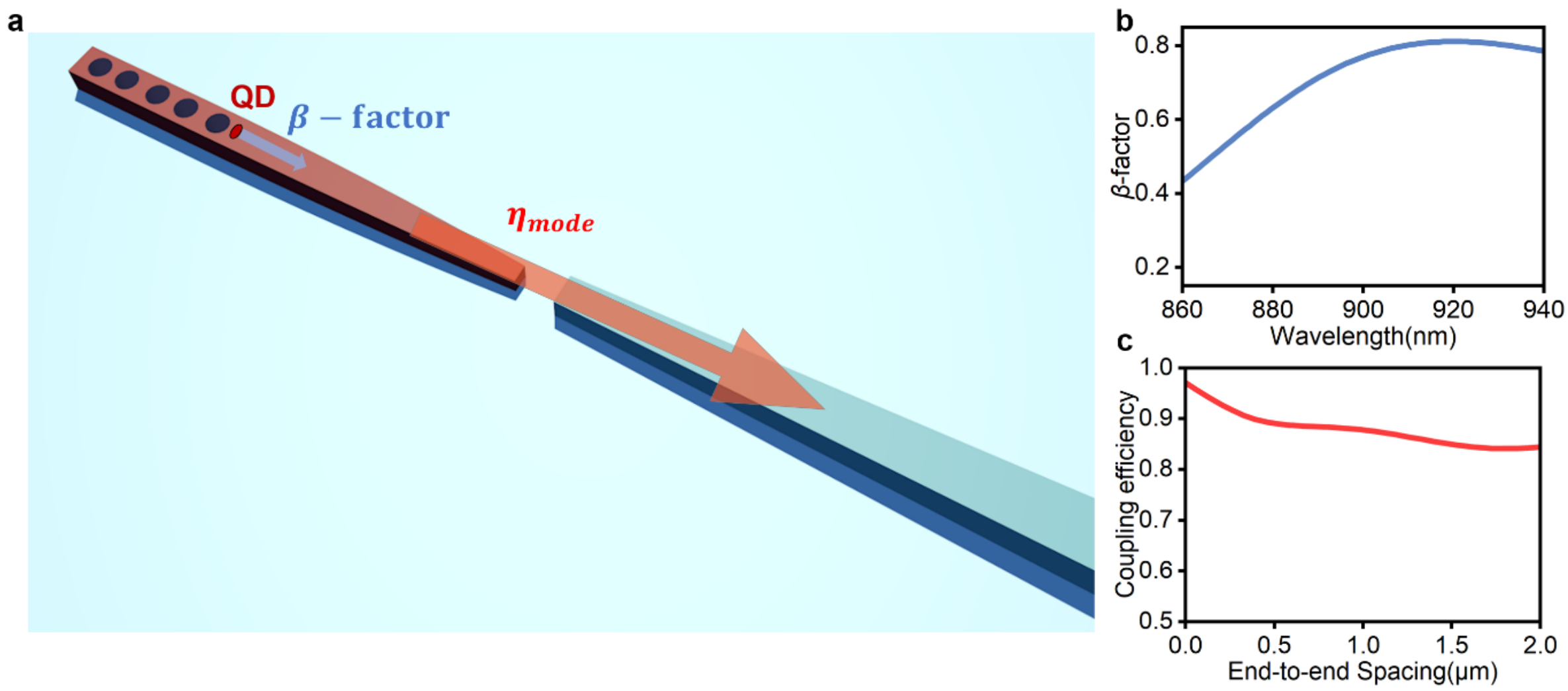

Figure S8. Simulation analysis of the source-to-waveguide coupling efficiency. (a) Schematic illustration of the hybrid interface employing an inverted taper design. The total probability of a photon coupling into the LTOI waveguide is determined by the product of the quantum dot's spontaneous emission coupling factor ($\beta - factor$) and the structural mode transfer efficiency (η_{mode}). (b) Calculated $\beta - factor$ describing the coupling of the quantum

dot's spontaneous emission into the GaAs nanobeam fundamental mode. (c) Calculated adiabatic mode transfer efficiency (η_{mode}) as a function of the end-to-end spacing between the waveguides.

To benchmark this measured efficiency, we performed a comprehensive component-by-component breakdown of the theoretical efficiency at the same position (the first objective lens). The total theoretical extraction efficiency incorporates the following key components:

1) Source-to-Waveguide Coupling ($\eta_{QD-LT} \approx 0.80$): This represents the total probability of a photon emitted by the QD successfully coupling into the LTOI waveguide mode. It is the product of two sub-processes: the coupling of the QD's spontaneous emission into the fundamental mode of the GaAs nanobeam ($\beta - factor \approx 0.82$, simulated at 920 nm) and the subsequent adiabatic mode transfer from the GaAs nanobeam to the LTOI waveguide ($\eta_{mode} \approx 0.98$). This high structural mode transfer efficiency (>85% as generally stated in the main text, peaking at ≈ 0.98 for the optimized spacing) is achieved via the inverted taper design (Fig. S8c).
2) Propagation Efficiency ($\eta_{trans} \approx 1$): Given the short device length and the optimized theoretical design, we approximate the transmission efficiency as unity.
3) Device Insertion Loss ($\eta_{MZI} \approx 0.92$): The single photons pass through two multimode interference (MMI) couplers within the MZI. Simulations indicate $\sim$4% loss per MMI ($0.96 \times 0.96 \approx 0.92$).

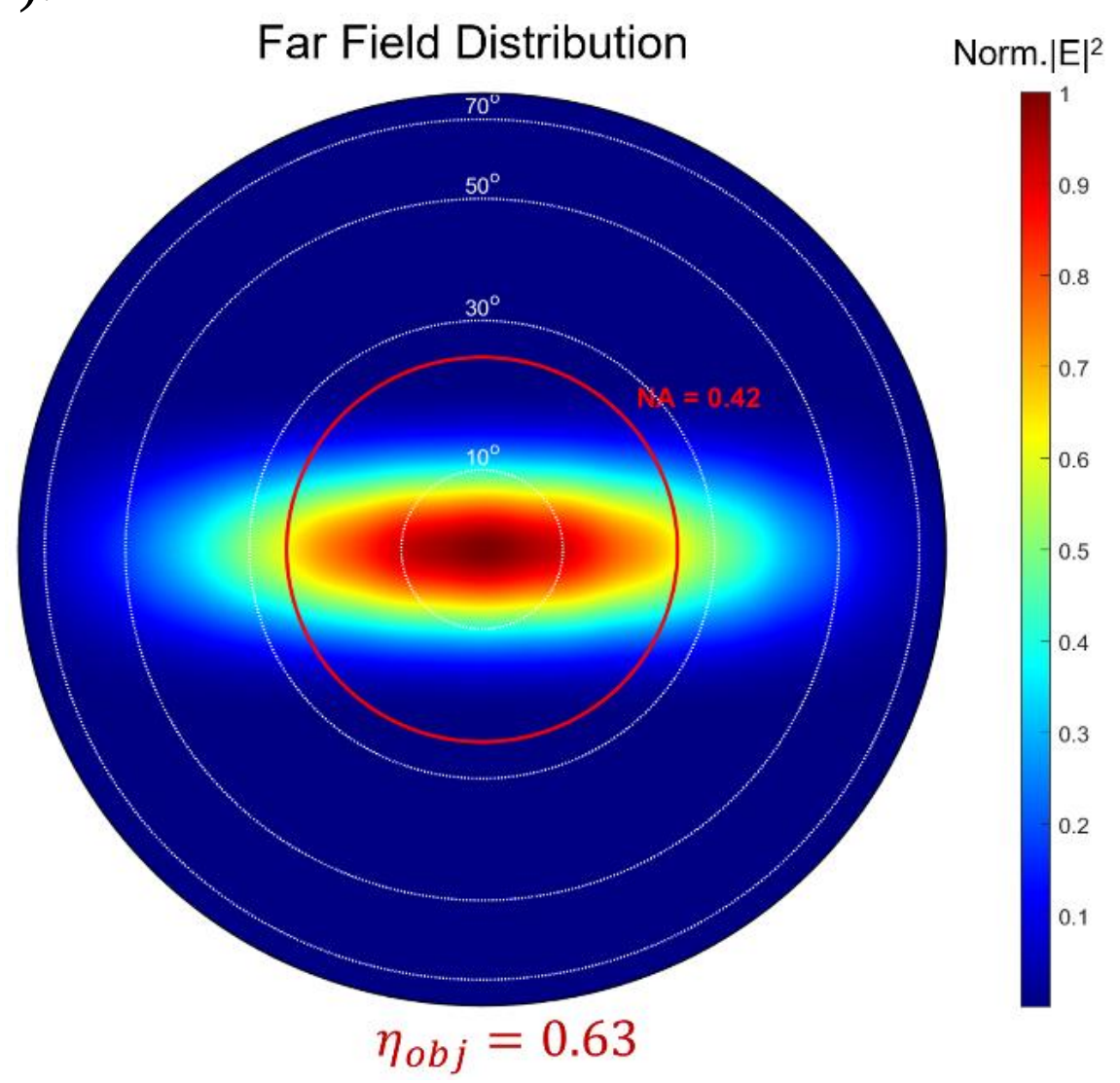

Figure S9. Far-field emission characteristics analysis. Simulated far-field radiation intensity distribution ($|E|^2$) emitted from the LTOI waveguide facet. The red circle delineates the acceptance cone of the objective lens (*NA*=0.42), which collects approximately 63% of the total radiated power.

4) Objective Collection Efficiency ($\eta_{obj} \approx 0.63$): Light out-couples from the waveguide facet. Due to the optical confinement in the LT ridge waveguide, the beam diverges significantly. As shown in Figure S9, our finite-difference time-domain (FDTD) simulation indicates that the objective lens with *NA*=0.42 (marked by the red circle) collects approximately 63% of the total emitted energy.

Multiplying these simulated components yields a theoretical extraction efficiency of:

$$\eta_{sim} = \eta_{QD-LT} \times \eta_{trans} \times \eta_{MZI} \times \eta_{obj} \approx 0.80 \times 1 \times 0.92 \times 0.63 \approx 0.46$$

The measured efficiency (29.7%) is lower than the theoretical limit (46%), a difference we attribute to experimental constraints and fabrication imperfections not captured in ideal

models—specifically, scattering at the hybrid interface and waveguide sidewalls, non-ideal reflectivity of the GaAs mirror, and potential misalignment. However, these are addressable engineering challenges. Our analysis confirms the high intrinsic efficiency of the hybrid interface, suggesting that future optimizations in etching quality and end-facet design could significantly boost overall performance.

VIII. Two-photon interference and visibility correction

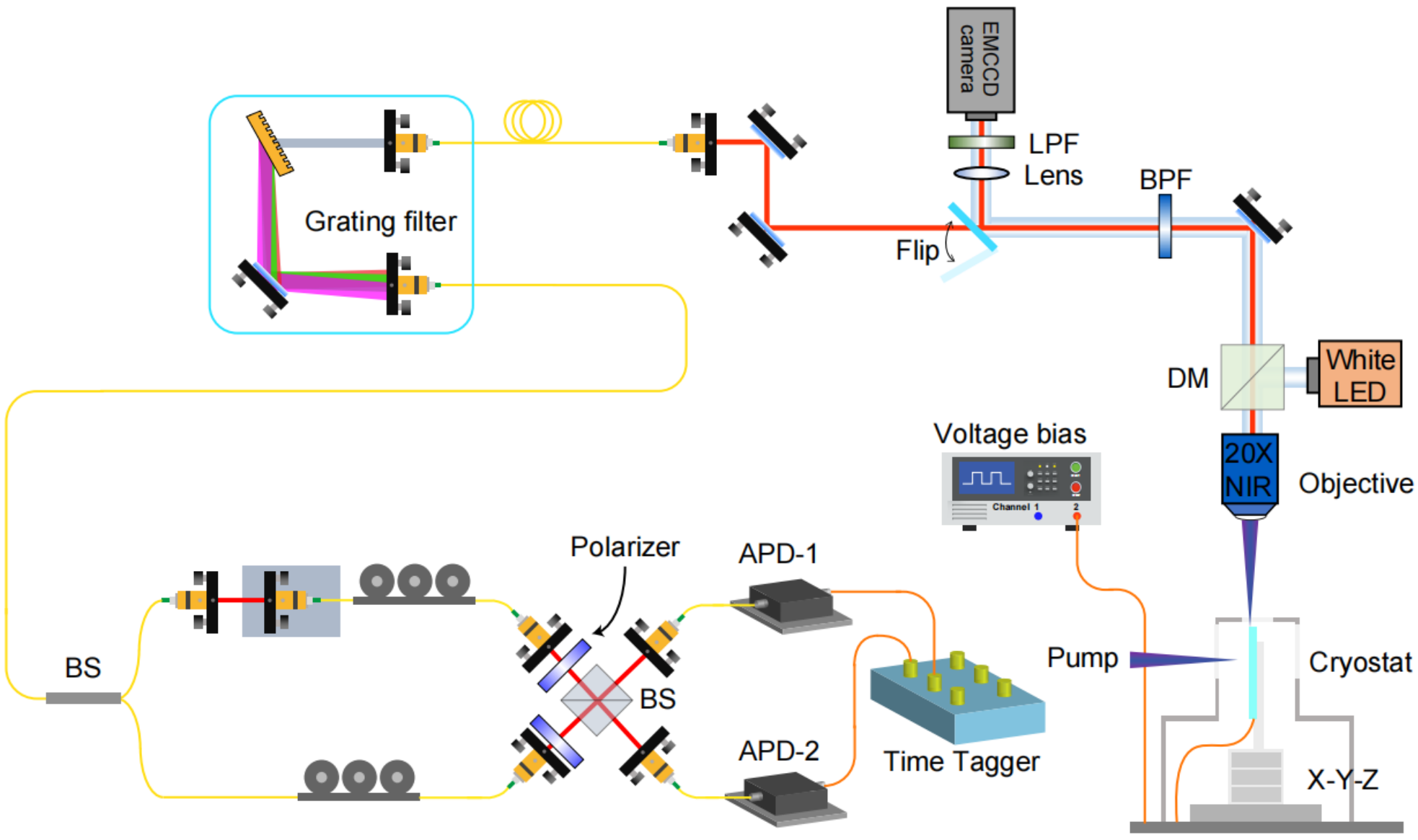

Figure S10. Schematic of the off-chip Hong-Ou-Mandel (HOM) two-photon interference setup.

To measure the indistinguishability of the emitted single photons, we performed a Hong-Ou-Mandel (HOM) interference experiment using an unbalanced MZI (Fig. S10). The optical delay difference between the two arms was set to match the repetition period of the pulsed excitation laser ($\sim 12.5\ ns$). A half-wave plate (HWP) and a polarizing beam splitter (PBS) were used to control the polarization state of the photons, allowing us to switch between co-polarized (parallel) and cross-polarized (orthogonal) configurations. The output photons are then detected by APDs and analyzed using a coincidence counter.

The raw two-photon interference visibility is calculated directly from the areas of the central coincidence peaks:

$$V = 1 - \frac{A_{\parallel}}{A_{\perp}}$$

where $A_{\parallel}$and $A_{\perp}$are the areas of the zero-delay peaks for co-polarized ($A_{\parallel}$ = 0.119(2)) and cross-polarized ($A_{\perp}$ = 0.514(3)) photons, yielding V =76.9 $\pm$ 0.4%.

IX. Comparison of Modulation Mechanisms for Integrated Deterministic Sources

To comprehensively benchmark our platform against the state-of-the-art, we compare the key parameters of the primary modulation mechanisms used for integrating deterministic single-photon sources with fast reconfigurability. These mechanisms include Thermo-optic, MEMS, Acousto-optic (SAW), and Electro-optic (GaAs and LTOI) schemes.

As detailed in Table S1, each platform faces specific physical and engineering trade-offs, which our hybrid LTOI approach aims to resolve:

1) Thermo-optic Tuning[1,2]: Thermo-optic phase shifters are the standard for room-temperature photonics but face severe limitations in quantum applications. The milliwatt-level heat dissipation can disturb the cryostat temperature, causing spectral drifting of Quantum Dots (QDs). Furthermore, the slow thermal response (kHz) makes them unsuitable for feed-forward operations, which require nanosecond-scale latencies.
2) MEMS Actuation[3]: Nanomechanical (MEMS) approaches successfully address the heat issue. However, their switching speed is physically limited by mechanical inertia to the MHz regime. This bandwidth is insufficient for the rapid active routing required in modern quantum computing architectures.
3) Acousto-optic (SAW)[4]: Surface acoustic wave devices achieve GHz speeds but are inherently resonant, operating efficiently only at specific RF frequencies (narrowband). This lacks the broadband DC-to-RF flexibility required for arbitrary circuit reconfiguration.
4) GaAs Electro-optic[5]: Monolithic GaAs electro-optic modulators offer high speeds. However, efficient modulation often requires doped layers or complex suspended structures, which can introduce additional propagation loss or fabrication challenges that compromise the yield of large-scale circuits.
5) Our Solution (LTOI Pockels Effect): Our work leverages the LTOI platform to overcome these bottlenecks. By harnessing the Pockels effect in LTOI, we achieve the GHz-bandwidth required for fast feed-forward control while maintaining negligible static power dissipation and low optical loss. This combination, enabled by our heterogeneous integration, provides a robust solution for scalable, active quantum photonic processors.

Table S1: Comparison of modulation mechanisms for integrated deterministic sources.

Modulation Mechanism	Integration Type	Speed / Bandwidth	Heat Load / Cryo-Compatibility
**Thermo-optic**	Hybrid (SiO_xN_y) [1]<br>Hybrid (SiN) [2]	**Slow** (~kHz)	**High (mW dissipation, incompatible with dense integration at 4K)**
**MEMS (Mechanical)**	Monolithic (GaAs) [3]	**Medium** (~10 MHz)	**Low (Capacitive, excellent for cryo)**
**Acousto-optic (SAW)**	Monolithic (GaAs) [4]	**Fast** (~GHz)	**Medium (RF heating, Fixed resonance frequency)**
**Electro-optic (GaAs)**	Monolithic (GaAs) [5]	**Fast** (~GHz)	**Medium/High (Trade-off between propagation loss in doped layers and modulation efficiency)**
**Electro-optic (Pockels) This Work**	Heterogeneous	**Fast** (> GHz)	**Low (Voltage-driven, negligible static power, low loss)**

X. Comparison with State-of-the-Art Quantum Photonic Processors

To rigorously assess the potential of our hybrid quantum dot-lithium tantalate (QD-LT) platform within the broader quantum hardware landscape, we analyzed established architectures based on three critical performance metrics: source determinism, reconfiguration, and detection integration. As detailed in Table S2, while existing platforms excel in specific metrics, they face inherent trade-offs that prevent the simultaneous realization of all key requirements for active quantum processing.

1) The first category encompasses heterogeneous integration efforts that have achieved the crucial step of combining high-quality III-V quantum dots with low-loss waveguides (such as Si3N4 or Si)[6–8]. However, these platforms have not yet integrated high-speed single-photon modulators into their demonstrated on-chip devices. Instead, they typically rely on thermo-optic phase shifters for tuning. This approach is limited by inherent thermal inertia, which restricts modulation speeds to the kHz range. Furthermore, the associated heat dissipation poses a significant challenge to maintaining the strict cryogenic stability required for quantum dot operation.
2) The second category comprises monolithic III-V platforms, which successfully integrate sources, waveguides, and detectors (SNSPDs) on a single chip[9,10]. However, achieving low-loss, high-speed electro-optic modulation on GaAs is fundamentally challenging; it often requires doping that introduces free-carrier absorption, creating a trade-off between propagation loss and modulation speed that limits the scale of the processor.
3) The third category includes large-scale reconfigurable silicon/MEMS circuits. While *Seok et al.* demonstrated impressive scalability with 64×64 switch matrices[11] and *Gyger et al.* solved the cryogenic compatibility issue using MEMS[12], both approaches face bandwidth limitations. MEMS actuators are restricted by mechanical inertia to MHz speeds.
4) The fourth category represents high-speed LNOI platforms, which offer GHz-bandwidth modulation and detection but lack integrated deterministic sources[13,14]. Reliance on external probabilistic sources (e.g., SPDC) or off-chip lasers introduces significant coupling losses and hinders the scaling of multi-photon logic.

In contrast, the hybrid LTOI-QD platform presented here uniquely bridges the gap between "Deterministic Generation" and "Ultra-fast Routing." By heterogeneously integrating InAs QDs with thin-film Lithium Tantalate, we combine the deterministic photon emission of the former with the superior Pockels effect of the latter. As demonstrated in our work, this allows for modulation bandwidths exceeding 40 GHz—orders of magnitude faster than MEMS or thermal approaches—without the propagation losses associated with doped GaAs or the coupling losses of external sources. This performance improvement is critical because it unlocks the potential for active quantum feed-forward (measuring a qubit and reconfiguring the circuit within the photon’s lifetime), a capability that is indispensable for future fault-tolerant quantum computing.

Table S2: Comparison of this work with state-of-the-art quantum photonic processors.

Platform Category	Representative Works	Integrated Deterministic Source?	Modulation Mechanism & Speed	Integrated Detection?	Critical Limitation for Active Quantum Processing
**I. Heterogeneous Integration (Source-focused)**	Chanana et al. (2022)[7]	Yes	No	No	**Lacking high-speed single-photon modulators.**
**II. Monolithic III-V**	Schwartz et al. (2018)[9]	Yes	No	Yes	**Lacking high-speed single-photon modulators.**
**III. MEMS**	Seok et al. (2016) [11] Gyger et al. (2021)[12]	No	MEMS (MHz / kHz)	Yes	**Bandwidth Bottleneck: Response time (~μs) exceeds photon lifetime.**
**IV. High-Speed LNOI**	Lomonte et al. (2021)[13]	No	Electro-optic (> GHz)	Yes	**Missing Source: Relies on external inputs, limiting scalability.**
**This Work**	(Our Manuscript)	Yes	Electro-optic (> 40 GHz)	Compatible	**Lacking detection.**

Supplementary References

1. Ellis, D. J. P. *et al.* Independent indistinguishable quantum light sources on a reconfigurable photonic integrated circuit. *Appl. Phys. Lett.* **112**, 211104 (2018).
2. Elshaari, A. W. *et al.* On-chip single photon filtering and multiplexing in hybrid quantum photonic circuits. *Nat. Commun.* **8**, 379 (2017).
3. Papon, C. *et al.* Nanomechanical single-photon routing. *Optica* **6**, 524 (2019).
4. Bühler, D. D. *et al.* On-chip generation and dynamic piezo-optomechanical rotation of single photons. *Nat. Commun.* **13**, 6998 (2022).
5. Midolo, L. *et al.* Electro-optic routing of photons from a single quantum dot in photonic integrated circuits. *Opt. Express* **25**, 33514 (2017).
6. Kim, J.-H. *et al.* Hybrid integration of solid-state quantum emitters on a silicon photonic chip. *Nano Lett.* **17**, 7394–7400 (2017).
7. Chanana, A. *et al.* Ultra-low loss quantum photonic circuits integrated with single quantum emitters. *Nat. Commun.* **13**, 7693 (2022).
8. Davanco, M. *et al.* Heterogeneous integration for on-chip quantum photonic circuits with single quantum dot devices. *Nat. Commun.* **8**, 889 (2017).
9. Schwartz, M. *et al.* Fully on-chip single-photon Hanbury-Brown and Twiss experiment on a monolithic semiconductor–superconductor platform. *Nano Lett.* **18**, 6892–6897 (2018).
10. Reithmaier, G. *et al.* On-chip generation, routing, and detection of resonance fluorescence. *Nano Lett.* **15**, 5208–5213 (2015).
11. Seok, T. J., Quack, N., Han, S., Muller, R. S. & Wu, M. C. Large-scale broadband digital silicon photonic switches with vertical adiabatic couplers. *Optica* **3**, 64 (2016).
12. Gyger, S. *et al.* Reconfigurable photonics with on-chip single-photon detectors. *Nat. Commun.* **12**, 1408 (2021).
13. Lomonte, E. *et al.* Single-photon detection and cryogenic reconfigurability in lithium niobate nanophotonic circuits. *Nat. Commun.* **12**, 6847 (2021).
14. Najafi, F. *et al.* On-chip detection of non-classical light by scalable integration of single-photon detectors. *Nat. Commun.* **6**, 5873 (2015).
