# Supplementary material for: Hybrid integration of quantum dot single-photon sources with lithium tantalate photonics for on-chip routing
Source: Nat Commun. 2026 Jun 16;17:7638. doi: 10.1038/s41467-026-74029-5 (PMC13434732; doi:10.1038/s41467-026-74029-5)
Supplement: Supplementary file 2 [file 41467_2026_74029_MOESM2_ESM.pdf]

nature portfolio

Peer Review File

Hybrid Integration of Quantum Dot Single-Photon Sources with Lithium Tantalate Photonics for On-Chip Routing

Corresponding Author: Professor Jin Liu

This file contains all reviewer reports in order by version, followed by all author rebuttals in order by version.

Version 0:

Reviewer comments:

Reviewer #1

(Remarks to the Author)
Kaili Xiong et al. demonstrate dynamical single-photon routing from heterogeneously integrated quantum dot devices and an LTOI chip. InAs quantum dots provide an ideal source of bright and coherent single photons, while LTOI has recently attracted significant attention as an emerging photonic platform due to its low loss and capability of fast modulation even at low temperature. The authors effectively leverage the strengths of both platforms using a hybrid integration approach. Although several prior works have shown hybrid integration, most studies have focused on single-photon coupling or switching under static voltages. In contrast, the present manuscript successfully achieves dynamic modulation of on-demand single photons at high speed, clearly showing the advantages of the LTOI platform at low temperature and marking a meaningful advancement beyond earlier results.
Overall, the experiment is well supported by both simulations and measured data, and the manuscript is well organized. The achievement could be though as an progress in this field. Therefore, I recommend the paper for publication with minor comments listed below.
[1] Figure 1 illustrates a hybrid-integrated architecture with multiple arrays of single-photon sources. However, it is not clear how this scheme can be practically realized. In particular, considering the spectral randomness of quantum dots, spectral tuning of individual emitters on a chip is essential. This requirement and a possible solution to address it should be discussed in the manuscript.
[2] In addition to spectral randomness, positional randomness is another major challenge for quantum dot single-photon sources. The spatial position of each quantum dot can strongly affect device efficiency and thus the fabrication yield of integrated systems. A proper discussion of these difficulties associated with solid-state quantum emitters is necessary to provide a balanced perspective on the proposed approach.
[3] Fig. 3a shows the modulation depth of ~ 90%. But there is no explanation of the nonzero intensity. A clear discussion of the origin of the residual intensity is needed.
[4] The authors claim an on-chip extraction efficiency of 29% for the integrated quantum dot devices. Characterizing the efficiency in this hybrid-integrated platform is important for determining the dominant loss channels. The total extraction efficiency includes multiple components, such as the single-photon extraction efficiency from the GaAs nanobeam, coupling efficiency between the GaAs and LTOI platforms, propagation loss in the LTOI waveguide, and the collection efficiency at the waveguide output. Listing more details on the contributions of each component would provide important information about the integrated devices.

Reviewer #2

(Remarks to the Author)
In this work, K. Xiong et al. report the first demonstration of a quantum dot single-photon source integrated on-chip with a nanophotonic electro-optic modulator. In my opinion, this can be considered as a milestone achievement in the field of integrated quantum photonics, making the article suitable for publication on Nat Comm. However, before the article gets published, there are some points that I would like the authors to carefully address:

1) A first concern is that, with the chosen geometry, the waveguide is likely multimode at a 900 nm wavelength. And the excitation of higher order modes in a waveguide is known to be highly detrimental for the coherence of a quantum state. The fact that the waveguide is operated in a multimode regime is in my opinion quite evident from the plots of Fig. S3b, where one can see that the measured transmission spectrum displays several dips at random wavelengths. The situation gets in fact worse for the spiral with a 17 cm length, since in a longer waveguide the excitation of higher order modes becomes more likely.
As an additional concern, shallow-etched waveguides are often affected by problems of TE-TM hybridization.
Ideally, to verify that working with a multimode waveguide, or TE-TM hybridization, is not an issue, the authors should: 1) perform an HOM experiment off-chip by interfering two photons from the quantum dot on a 50:50 beam splitter; 2) repeat the experiment on-chip and check whether the measured HOM visibility is the same as the one measured off-chip. If, understandably, a similar measurement is not possible, this point should at least thoroughly discussed in the manuscript.

2) One concern that I have is that, even in theory, the coupling efficiency between the QD single-photon source and the LTOI waveguide is quite limited. In fact, while 80-90% might seem already a good value, for demanding applications such as fault-tolerant quantum computing, or the demonstration of quantum computational advantage by boson sampling, one would like to work with a coupling efficiency as close as possible to 100%. Can the authors discuss alternative configurations enabling a higher coupling efficiency? (see, e.g., the configuration proposed in https://www.mdpi.com/2073-4352/11/11/1376)

3) In my opinion, the article fails to discuss precisely the various perspectives enabled by the integration of QD single-photon sources with fast electro-optic modulators + relevant references. These could include, for instance, the realization of demultiplexed single-photon sources, fusion measurements for cluster state quantum computing, or frequency shifting of single photons. I would ask the authors to add a similar discussion in the article.

4) In the article, the QD is operated under non-resonant excitation. However, to achieve a single-photon purity and indistinguishability as high as possible, a resonant excitation scheme is required. Can the authors discuss alternative configuration for the GaAs waveguide that could enable to operate the QD under resonant excitation?

5) A relevant article (published only very recently) which is to not cited in the manuscript, is: https://www.nature.com/articles/s41563-025-02398-1 . In particular, this article demonstrates that, by leveraging the piezoelectric properties of lithium niobate, it is possible to spectrally tune individual QDs and achieve indistinguishable emission between separated single-photon sources. I think this is a very important perspective which deserves to be discussed.

6) Lines 48-50:
“Notably, the successful integration of cryogenic-compatible LNOI circuits with superconducting nanowire single-photon detectors (SNSPDs) has underscored their immense potential for quantum technologies (7,8).”
Ref. 8 doesn’t demonstrate the integration of LNOI circuits with SNSPDs. More relevant articles that the authors might want to cite in addition to Ref. 7, are: https://pubs.aip.org/aip/apl/article/116/15/151102/38104/Lithium-niobate-on-insulator-waveguide-integrated , https://pubs.acs.org/doi/10.1021/acs.nanolett.3c02324
, https://pubs.acs.org/doi/full/10.1021/acsphotonics.3c01628 .

7) Lines 52-55:
“Moreover, the LTOI platform exhibits advantages such as expanded transparent window (0.28-5.5um), decreased optical anisotropy, stable operation at low frequency drive and high optical power, which renders it beneficial for building photonic integrated 54 circuits (PICs) at shorter wavelengths (9-11).”
Here one could argue that: i) since the emission wavelength of QDs is 900 nm, which is well within the transparency range of LN, having an expanded transparency window is not much useful; ii) while the DC bias drift of LNOI is a big issue at room temperature, it gets strongly suppressed, and becomes almost negligible, at cryogenic temperatures (see https://www.nature.com/articles/s41467-021-27205-8 and also https://opg.optica.org/ol/fulltext.cfm?uri=ol-50-17-5398)

8) Lines 261-262:
“This analysis yielded an on-chip extraction efficiency of 29.7%, representing the fraction of emitted photons successfully coupled into and guided by the hybrid GaAs-LT waveguide system”.
Maybe I didn’t understand well what the authors mean, but 29.7%, according to the Supplementary Material, shouldn’t be the extraction efficiency at the first objective? I would not call this “on-chip” extraction efficiency since, according to the Supplementary, it also takes into account the imperfect overlap of the waveguide mode with the objective (or with the fiber placed after the objective, see the next comment).

9) One question strictly related to the previous one. Supplementary Materials, lines 158-160:
“However, the mode overlap between the output field of the LT waveguide and the Gaussian mode accepted by the NA=0.42 objective was only 0.39, due to the lack of structural optimization at the waveguide output facet.”
I am a bit confused by this part, but I would be prone to think that, if the NA of the objective is sufficiently high, all the light should be collected by the objective independently from the exact shape of the waveguide mode. On the other hand, one should certainly take into account the reduced overlap of the collected light with the fiber placed after the objective. But perhaps this is just what the authors mean.

10) Lines 281-282:
“The butt-coupled waveguide architecture achieves >85% mode coupling efficiency”.
According to the supplementary, it shouldn’t be slightly lower than 80%?

11) Lines 283-289:
“Looking forward, full-scale quantum information processing demands ultra-low-loss delay lines (~1 dB/m) and integrated superconducting detectors (e.g., SNSPDs), both challenged by LT's current propagation loss and fabrication complexity (39). Heterogeneous integration with SiN waveguides (~1dB/m loss) (40) and monolithic SNSPD fabrication on LN substrates (41,42) provide viable solutions.”
I have two observations: i) despite the fact that prop loss down to 1 dB/m have been also demonstrated on LNOI (and I would assume the same should be possible with LTOI) integrating everything on-chip is probably hopeless, and one should also efficiently couple the waveguides with optical fibers for the realization of very long delay lines; ii) Ref. 41 was already cited as Ref. 7, while Ref. 42 doesn’t demonstrate the integration of SNSPDs with LNOI waveguides (see comment 6 for more relevant references). Please carefully check if similar issues are present with also other references within the manuscript.

12) Lines 290-293:
“As ultra-low-loss photonics and cryogenic detector technologies mature, this convergence enables fully integrated quantum processors for boson sampling, quantum walks, and entanglement distribution—unlocking optical quantum computing's potential within 292 near-term experimental reach.”
Please discuss more precisely the perspectives enabled by the integration of QDs with fast electro-optic modulators (see also comment 3) and add some relevant references.

Reviewer #3

(Remarks to the Author)
Editor-specific Questions:
1. What are the noteworthy results?
- The authors integrate of a single photon source comprised of an indium-arsenide quantum dot into a lithium tantalate photonic platform with high-speed configurability.
- The authors show a relatively high, but not record, photon extraction efficiency whilst integrating an additional component into the single photon source (the modulator) at cryogenic temperatures.
2. Will the work be of significance to the field and related fields? How does it compare to the established literature? If the work is not original, please provide relevant references.
The work has significance as a technology demonstrator, specifically for hybrid hardware platforms for integrated photonics with deterministic single photon sources. For the broader photonic quantum processor, and quantum computing fields in general, with the limited demonstration presented here, the significance is still unclear.
- Without any demonstration of source indistinguishability, photon interference, or gate operations, the link to being a suitable hardware platform for quantum photonic processors is tenuous at best.
- A comparison should be made to other papers that have looked at integrating deterministic single photon sources with fast reconfigurability to compare key parameters.
- A comparison of the potential of this platform to other quantum photonic processors would be instructive as to the value of this work in the broader quantum hardware community. Currently only a comparison to prior work on very similar photonic platforms is offered. This comparison should show how this work has improved the performance of photonic platforms based on deterministic single photon sources compared to other photonic platforms.
- What is the yield of the transfer process, and are there limitations on the maximum possible yield? Is this fabrication methodology scalable in terms of both yield and volume? In the supplementary it is mentioned that three GaAs waveguides are simultaneously transferred, and yet there is only data shown for one of these. Do the other two not work, or not work as well? This information should be included for readers to effectively evaluate the significance of this result.
3. Does the work support the conclusions and claims, or is additional evidence needed?
The data supports the conclusions and claims made about the work undertaken here, however some of the broader implications claimed seem to be far-fetched.
- In the abstract the authors state: "The scalability of quantum photonic processors relies on simultaneously integrating deterministic single-photon sources, low loss photonic circuitry and fast reconfigurability." This is quite a strong statement considering some of the largest photonic quantum computing companies, (e.g. PSIQuantum, Xanadu) do not follow this approach. I also do not think the contents of this paper supports this conclusion. I would recommend including an analysis of this statement in the body of the paper, providing references to prove other photonic quantum computing approaches infeasible, and to prove this approach as the only suitable one.
- The authors offer a list of advantages of lithium tantalate over lithium niobate, but it is not clear to me how these properties are an advantage for building a quantum photonic processor. Specifically how do an expanded transparency window, and decreased optical anisotropy help for this endeavour?
- The electro-optic modulators demonstrated here have a large footprint (>3mm long). How does the size of this platform affect its integration density potential, and its utility for small and large-scale quantum photonic processors, especially concerning cryogenic cooling space requirements? I think some numbers are necessary to provide a convincing claim that this platform is useful for quantum photonic processors.

4. Are there any flaws in the data analysis, interpretation and conclusions? - Do these prohibit publication or require revision?
I found no flaws in the data analysis that would prevent publication, however there are some things that should be more precisely stated.
- The estimation of detection efficiency does not contain any error margins in the transmission of various optical components. While this would normally be immaterial, when the total system transmission is so poor 1%, these uncertainties may make a significant difference to the uncertainty in the final extraction efficiency. This final extraction efficiency should be quoted with

an uncertainty.
- A plot of the experimental output mode profile that was measured to have an overlap of 0.39 with the objective would also be a good visual support to the discussion about system collection efficiency. A discussion on the use of tapers or gratings to improve this coupling efficiency, and how far it could be improved could increase the impact by allowing readers to recognise the potential performance improvements available in this system.
5. Is the methodology sound? Does the work meet the expected standards in your field?
The methodology is sound and meets expected standards for original research of moderate importance in this field.
- How are differences in coupling efficiency accounted for during the measurement of propagation loss through the cutback method. The standard method for propagation loss is to measure the quality factor of a resonant structure. Why is this non-standard method used?
- The performance of the modulation should be reported using with common measurements for modulation performance, including insertion loss, 3dB modulation bandwidth, and Extinction ratio in dB.
- Why are all of the transmission values for optical components so low? Is nothing in this setup anti-reflection coated, as would be standard?
6. Is there enough detail provided in the methods for the work to be reproduced?
There is enough detail in the methods to reproduce this work with the following exception:
- A reference, or more detail, is necessary to be able to reproduce the GaAs/InAs quantum dot waveguide fabrication method.

Journal-specific Guidelines:
• The quality of the data — whether they are technically sound, obtained with appropriate techniques, analysed and interpreted carefully, and presented in sufficient detail.
• While Fig 1a. is appropriate for a later section discussing the future potential in this platform, I believe a schematic of the actual device under test is much more suitable for inclusion in a figure presenting experimental results. Furthermore without a schematic of the actual device under test it is not so clear what this paper has achieved.
• Fig 1b,c,d seems like a very strange way to represent this data. As there is very little wavelength dependency, why not plot the independent variable vs the dependent variable, and represent the wavelength as a series. This would make the plots clearer. Additionally it would be instructive to include the experimentally measured values of spacing, lateral offset, and angle offset for the three placed GaAs waveguides either in the text or on these plots, to demonstrate what values were achieved.
• In figure 4a, the uncertainty of the fitted radiative lifetime should be quoted.
• In figure 4c, the count rate appears to peak, and then reduce with increasing pump power. What is the likely cause of this?

• The level of support for the conclusions — whether sufficiently strong evidence is provided for the authors' claims and all appropriate controls have been included.
Covered in Editors Q3
• The potential significance of the results — whether these results will be important to the field and advance understanding in a way that will move the field forward. (Note that posting of preprints and/or conference proceedings does not compromise novelty.)
• Covered in Editors Q2
Other Comments:
The labelling in fig 1 seems odd with fig f coming before fig e.

Version 1:

Reviewer comments:

Reviewer #1

(Remarks to the Author)
The authors have addressed my previous comments and concerns in a satisfactory manner. The revised manuscript is now clearer and much improved. Therefore, I recommend that the manuscript is suitable for publication.

Reviewer #2

(Remarks to the Author)
The authors have adequately addressed all my comments and the article can be published.

Reviewer #3

(Remarks to the Author)
The authors have extremely thoroughly addressed all my concerns, and have significantly improved the contextualisation of this result within the wider field, making the work more readily accessible to a larger audience across quantum computing hardware platforms. The work is of particular significance to the field of quantum photonic computation, the data supports the claims, the methodology is sound, meeting expected standards in the field, and as far as I can see the published information allows reproducibility.

Open Access This Peer Review File is licensed under a Creative Commons Attribution 4.0 International License, which permits use, sharing, adaptation, distribution and reproduction in any medium or format, as long as you give appropriate credit to the original author(s) and the source, provide a link to the Creative Commons license, and indicate if changes were made.
In cases where reviewers are anonymous, credit should be given to 'Anonymous Referee' and the source.
The images or other third party material in this Peer Review File are included in the article's Creative Commons license, unless indicated otherwise in a credit line to the material. If material is not included in the article's Creative Commons license and your intended use is not permitted by statutory regulation or exceeds the permitted use, you will need to obtain permission directly from the copyright holder.
To view a copy of this license, visit https://creativecommons.org/licenses/by/4.0/

Response Letter

Manuscript ID: NCOMMS-25-74738
Manuscript title: Hybrid Integration of Quantum Dot Single-Photon Sources with Lithium Tantalate Photonics for On-Chip Routing
Authors: Kaili Xiong, Defeng Shan, Xueshi Li, Ziliang Ruan, Bin Chen, Zhanling Wang, Jiawei Wang, Ying Yu, Wei Wu, Pingxing Chen, Jin Liu, Liu Liu, Yan Chen, Tian Jiang

Reviewer #1:

[Comment 1]

Kaili Xiong et al. demonstrate dynamical single-photon routing from heterogeneously integrated quantum dot devices and an LTOI chip. InAs quantum dots provide an ideal source of bright and coherent single photons, while LTOI has recently attracted significant attention as an emerging photonic platform due to its low loss and capability of fast modulation even at low temperature. The authors effectively leverage the strengths of both platforms using a hybrid integration approach. Although several prior works have shown hybrid integration, most studies have focused on single-photon coupling or switching under static voltages. In contrast, the present manuscript successfully achieves dynamic modulation of on-demand single photons at high speed, clearly showing the advantages of the LTOI platform at low temperature and marking a meaningful advancement beyond earlier results.
Overall, the experiment is well supported by both simulations and measured data, and the manuscript is well organized. The achievement could be though as an progress in this field. Therefore, I recommend the paper for publication with minor comments listed below.

Response:

We sincerely appreciate the reviewer positive feedback and valuable insights on our manuscript. In the following we provide point-to-point responses to address the concerns.

[Comment 2]

Figure 1 illustrates a hybrid-integrated architecture with multiple arrays of single-photon sources. However, it is not clear how this scheme can be practically realized. In particular, considering the spectral randomness of quantum dots, spectral tuning of individual emitters on a chip is essential. This requirement and a possible solution to address it should be discussed in the manuscript.

Response:

We sincerely thank the reviewer for this constructive comment. We agree that the spectral randomness of quantum dots (QDs) is a fundamental challenge for scaling up hybrid-integrated quantum photonic circuits, and individual spectral tuning is essential for the practical realization of the architecture shown in Figure 1a.
To address this concern, we have added a discussion in the revised manuscript regarding the practical implementation of on-chip tuning. We highlight two prominent, circuit-compatible solutions:

1) The Quantum-Confined Stark Effect (QCSE): Previous work by Aghaeimeibodi et al. [*Appl. Phys. Lett. 114, 071105 (2019)*] has demonstrated large wavelength tunability of individual

quantum dot emitters.

2) Piezoelectric Strain Tuning: Leveraging the piezoelectric properties of thin-film lithium niobate (TFLN), Wang et al. [*Nat. Mater. 24, 1898–1905 (2025)*] recently achieved a local, reversible strain-tuning technique. Notably, they demonstrated the scalability of this mechanism by independently tuning the emission spectra of 20 deterministic single-photon sources on a single chip.

These advancements provide a clear roadmap for the practical realization of multi-array architecture. We have updated the manuscript to reflect this discussion.

Manuscript Revision:

" Figure 1a illustrates the future architecture of this chip platform, where arrays of QDs can be integrated on-chip. A challenge to realizing this scalable multi-array architecture is the inherent spectral randomness of self-assembled QDs. The spectral inhomogeneity can be addressed by integrating circuit-compatible local tuning mechanisms. For instance, individual emitters can be tuned using local electric fields via the Quantum-Confined Stark Effect[41]. Furthermore, the intrinsic piezoelectric properties of ferroelectric platforms enable dynamic and reversible local strain tuning, which can independently tune the spectra of individual single-photon sources over a large range, offering a practical pathway to scalable on-chip quantum networks[42]."

41. Aghaeimeibodi, S., Lee, C.-M., Buyukkaya, M. A., Richardson, C. J. K. & Waks, E. Large stark tuning of InAs/InP quantum dots. *Appl. Phys. Lett.* **114**, 071105 (2019).

42. Wang, X. *et al.* Large-scale quantum dot–lithium niobate hybrid integrated photonic circuits enabling on-chip quantum networking. *Nat. Mater.* **24**, 1898–1905 (2025).

[Comment 3]

In addition to spectral randomness, positional randomness is another major challenge for quantum dot single-photon sources. The spatial position of each quantum dot can strongly affect device efficiency and thus the fabrication yield of integrated systems. A proper discussion of these difficulties associated with solid-state quantum emitters is necessary to provide a balanced perspective on the proposed approach.

Response:

We thank the reviewer for this insightful comment. We agree that the spatial randomness as a consequence of self-assembly is a critical factor that determines the coupling efficiency and the overall fabrication yield of integrated quantum systems.

In the revised manuscript, we have added a discussion on deterministic integration strategies to address positional randomness:

1) High-precision optical positioning: Techniques such as grid-marker-assisted photoluminescence (PL) imaging, leveraging dual-color LED co-illumination and EMCCD imaging, can localize single QDs with an average uncertainty of less than 30 nm [*Nat Commun 6, 7833 (2015)*]. This enables the subsequent deterministic alignment of photonic structures.

2) Cryogenic in situ electron-beam lithography: As demonstrated by Gschrey et al. [*Nat Commun* **6**, 7662 (2015)], integrating PL imaging, spectroscopy, and E-beam lithography in a

single cryogenic system allows for high-precision patterning without the need for complex coordinate transformations or external reference markers.

By incorporating these state-of-the-art methodologies, the positional randomness of solid-state emitters can be effectively managed, facilitating the high-yield fabrication of the proposed hybrid architecture.

Manuscript Revision:

" In addition to spectral alignment, the positional randomness of self-assembled QDs can be mitigated using grid-marker-assisted photoluminescence imaging combined with dual-color light-emitting diode (LED) co-illumination and electron-multiplying charge-coupled device (EMCCD) imaging, or via in situ cryogenic three-dimensional electron-beam lithography[43,44]. These methodologies provide a robust pathway for the deterministic and scalable production of high-performance quantum photonic circuits."

43. Sapienza, L., Davanço, M., Badolato, A. & Srinivasan, K. Nanoscale optical positioning of single quantum dots for bright and pure single-photon emission. *Nat. Commun.* **6**, 7833 (2015).

44. Gschrey, M. *et al.* Highly indistinguishable photons from deterministic quantum-dot microlenses utilizing three-dimensional in situ electron-beam lithography. *Nat. Commun.* **6**, 7662 (2015).

[Comment 4]

Fig. 3a shows the modulation depth of ~ 90%. But there is no explanation of the nonzero intensity. A clear discussion of the origin of the residual intensity is needed.

Response:

We thank the reviewer for this insightful observation regarding the modulation depth in Fig. 3a. Following the suggestion, we added a clear discussion on the origin of the residual (nonzero) intensity in the revised **Supplementary Information Note VI**.

The incomplete extinction (modulation depth of ~90%) at cryogenic temperatures is primarily attributed to the limitations of the electrical packaging used in the cryostat, specifically regarding impedance matching and wire bonding:

1) Impedance Mismatch: Unlike our room-temperature characterization where precise 50 Ω termination was applied, the cryogenic setup lacked perfect impedance matching throughout the transmission line. This mismatch causes signal reflections, preventing the effective voltage across the electrodes from fully reaching the required V_π for complete extinction.
2) Parasitic Inductance from Wire Bonding: As noted in the manuscript, the device was connected to the RF carrier via wire bonding for cryogenic operation, rather than using high-speed RF probes. The bonding wires introduce parasitic inductance, which acts as a low-pass filter and attenuates the RF signal reaching the traveling wave electrodes.

To verify that this is a packaging-induced bandwidth limitation rather than an intrinsic device defect, we performed additional measurements of the modulation depth at varying frequencies (100 MHz, 200 MHz, 400 MHz, and 800 MHz). As shown in the newly added Figure R1, the modulation depth decreases significantly as the frequency increases. This frequency-dependent roll-off confirms that the residual intensity is due to the RF attenuation caused by the wire bonds and impedance mismatch, preventing the drive signal from achieving the full amplitude required for 100% modulation depth.

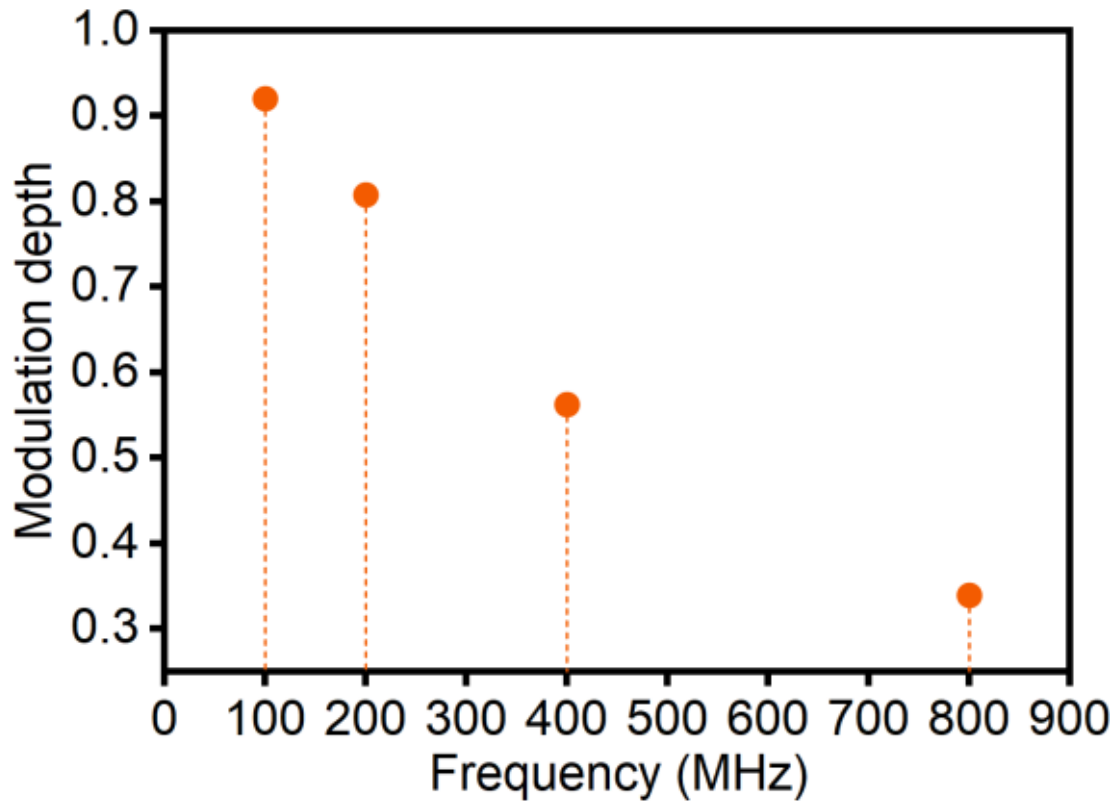

Figure R1. Measured modulation depth as a function of frequency at cryogenic temperature. The decreasing trend confirms that the modulation efficiency is limited by the RF packaging bandwidth (wire bonding and impedance mismatch).

[Comment 5]

The authors claim an on-chip extraction efficiency of 29% for the integrated quantum dot devices. Characterizing the efficiency in this hybrid-integrated platform is important for determining the dominant loss channels. The total extraction efficiency includes multiple components, such as the single-photon extraction efficiency from the GaAs nanobeam, coupling efficiency between the GaAs and LTOI platforms, propagation loss in the LTOI waveguide, and the collection efficiency at the waveguide output. Listing more details on the contributions of each component would provide important information about the integrated devices.

Response:

We thank the reviewer for pointing this out. We apologize for the lack of clarity regarding the efficiency characterization in the original manuscript. In the revised **Supplementary Information Note VII**, we have provided a detailed component-by-component breakdown based on our updated FDTD simulations and experimental parameters.

First, we clarify that the measured **29.7%** represents **the extraction efficiency at the first objective lens, excluding the subsequent fiber coupling losses**. To benchmark this result, we performed a detailed breakdown of the theoretical efficiency at the same position:

1) Source-to-Waveguide Coupling ($\eta_{QD-LT} \approx 0.80$): This aggregate efficiency represents the probability of a photon emitted by the quantum dot being successfully coupled into the LTOI waveguide mode. It is determined by the product of two sub-processes: the coupling of the quantum dot's spontaneous emission into the GaAs nanobeam fundamental mode ($\beta - factor \approx 0.82$) and the subsequent adiabatic mode transfer from the GaAs nanobeam to the LTOI waveguide ($\eta_{mode} \approx 0.98$). The high transfer efficiency is achieved through the optimized inverted taper design shown in Figure R2. We clarify that the value ">85%"

mentioned in the main text refers specifically to the structural mode transfer efficiency (η_{mode}), while the total source coupling efficiency ($\eta_{QD-LT} \approx 0.80$) accounts for the intrinsic $\beta - factor$.

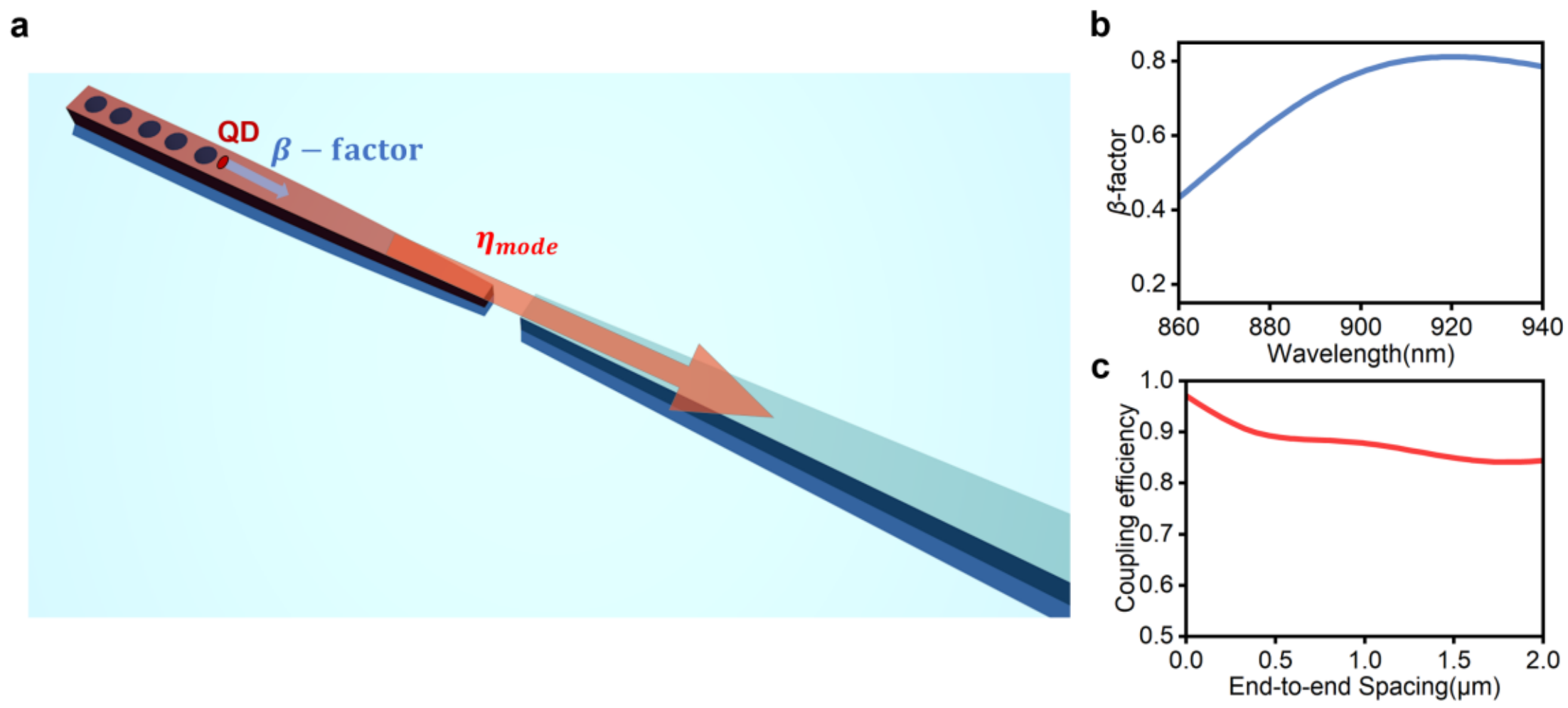

Figure R2. **Simulation analysis of the source-to-waveguide coupling efficiency.** (a) Schematic illustration of the hybrid interface employing an inverted taper design. The total probability of a photon coupling into the LTOI waveguide is determined by the product of the quantum dot's spontaneous emission coupling factor ($\beta - factor$) and the structural mode transfer efficiency (η_{mode}). (b) Calculated $\beta - factor$ describing the coupling of the quantum dot's spontaneous emission into the GaAs nanobeam fundamental mode. At the wavelength of 920 nm, the simulation yields a $\beta - factor \approx 0.82$. (c) Calculated adiabatic mode transfer efficiency (η_{mode}) as a function of the end-to-end spacing between the waveguides. For the optimized configuration used in our experiment, the simulation demonstrates a high structural transfer efficiency of $\approx 98\%$.

2) Propagation Efficiency ($\eta_{trans} \approx 1$): Given the short device length and the optimized theoretical design, we approximate the transmission efficiency as unity.
3) Device Insertion Loss ($\eta_{MZI} \approx 0.92$): The single photons pass through two Multi-Mode Interference (MMI) couplers within the Mach-Zehnder Interferometer (MZI). Simulations indicate $\sim 4\%$ loss per MMI ($0.96 \times 0.96 \approx 0.92$).
4) Objective Collection Efficiency ($\eta_{obj} \approx 0.63$): Finally, the light out-couples from the waveguide facet. Due to the optical confinement in the LT ridge waveguide, the beam diverges significantly. As shown in Figure R3(a), our FDTD simulation indicates that the objective lens with NA=0.42 (marked by the red circle) collects approximately 63% of the total emitted energy.

Multiplying these simulated components yields a theoretical extraction efficiency of:

$$\eta_{sim} = \eta_{QD-LT} \times \eta_{trans} \times \eta_{MZI} \times \eta_{obj} \approx 0.80 \times 1 \times 0.92 \times 0.63 \approx 0.46$$

The measured efficiency (29.7%) is lower than the theoretical limit (46%), a difference we attribute to experimental constraints and fabrication imperfections not captured in ideal models—specifically, scattering at the hybrid interface and waveguide sidewalls, non-ideal reflectivity of the GaAs mirror, and potential misalignment. However, these are addressable engineering challenges. Our analysis confirms the high intrinsic efficiency of the hybrid interface, suggesting that future optimizations in

etching quality and end-facet design could significantly boost overall performance.

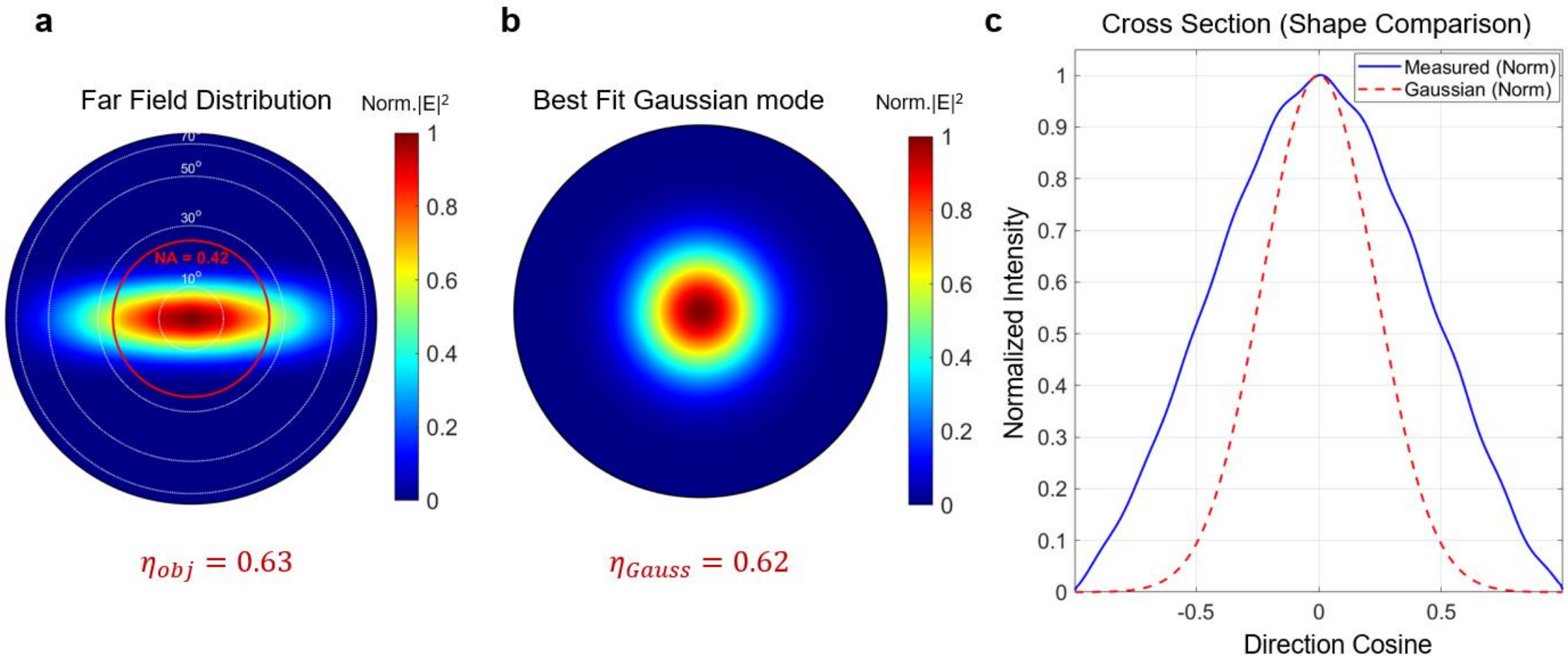

Figure R3. Far-field emission characteristics and fiber coupling analysis. (a) Simulated far-field radiation intensity distribution ($|E|^2$) emitted from the LTOI waveguide facet. The red dashed circle delineates the acceptance cone of the objective lens (NA=0.42), which collects approximately 63% of the total radiated power. (b) The fundamental Gaussian mode profile that provides the maximum overlap with the emitted far-field pattern. The calculated overlap integral yields a mode matching efficiency of $\eta_{Gauss} \approx 0.62$. (c) Comparison of the cross-sectional intensity profiles between the waveguide far-field emission (solid line) and the fitted Gaussian mode (dashed line). The deviation between the two curves visually illustrates the mode mismatch responsible for energy loss.

Reviewer #2:

[Comment 0]

In this work, K. Xiong et al. report the first demonstration of a quantum dot single-photon source integrated on-chip with a nanophotonic electro-optic modulator. In my opinion, this can be considered as a milestone achievement in the field of integrated quantum photonics, making the article suitable for publication on Nat Comm. However, before the article gets published, there are some points that I would like the authors to carefully address:

Response:

We sincerely thank the reviewer for the positive evaluation of our work and for the encouraging comments. We are particularly grateful for the reviewer's recognition of this work as a "milestone achievement" in the field of integrated quantum photonics. We have carefully revised the manuscript. In the following, we give a point by point response.

[Comment 1]

A first concern is that, with the chosen geometry, the waveguide is likely multimode at a 900 nm wavelength. And the excitation of higher order modes in a waveguide is known to be highly detrimental for the coherence of a quantum state. The fact that the waveguide is operated in a multimode regime is in my opinion quite evident from the plots of Fig. S3b, where one can see that the measured transmission spectrum displays several dips at random wavelengths. The situation gets in fact worse for the spiral with a 17 cm length, since in a longer waveguide the excitation of higher order modes becomes more likely.

As an additional concern, shallow-etched waveguides are often affected by problems of TE-TM hybridization.

Ideally, to verify that working with a multimode waveguide, or TE-TM hybridization, is not an issue, the authors should: 1) perform an HOM experiment off-chip by interfering two photons from the quantum dot on a 50:50 beam splitter; 2) repeat the experiment on-chip and check whether the measured HOM visibility is the same as the one measured off-chip. If, understandably, a similar measurement is not possible, this point should at least thoroughly discussed in the manuscript.

Response:

We thank the reviewer for this critical observation regarding the waveguide mode properties. We agree that preventing higher-order mode excitation is essential for preserving the coherence of the quantum state. Per the reviewer's suggestion to thoroughly discuss this point, we provide the following clarification on why the multimode behavior observed in the characterization structures (Fig. S4) does not impact the performance of the actual quantum routing device.

1) **Origin of Spectral Ripples in Fig. S4b.** The spectral fluctuations observed in Fig. S4b originate primarily from the input/output edge couplers rather than the intrinsic propagation of the test waveguide. The characterization structure consists of a narrow test waveguide (1 μm width) connected to wide edge couplers (3 μm width) to facilitate fiber alignment. These 3 μm-wide sections support multiple higher-order modes, and the excitation and interference of these modes are significantly stronger than in the single-mode test waveguide. Although we performed normalization to remove the system envelope, the multimode interference patterns (ripples) vary between the reference and test structures, making it difficult to isolate the

intrinsic mode purity of the test waveguide from the dominant noise introduced by the wide couplers.

2) **Geometric Distinction.** There is a fundamental geometric distinction between the loss-characterization spirals and the actual modulator device that prevents mode mixing in the latter. The test structure is a 17 cm long spiral requiring numerous bends to fold the optical path; both the extreme length and the cumulative scattering at these bends significantly increase the probability of higher-order mode excitation. In contrast, the active device used for single-photon routing is extremely compact, with a modulation length of only ~3 mm and contains only a single gentle bend ($R = 200\ \mu m$). In this "short-path, few-bend" regime, the excitation of higher-order modes is negligible compared to the long spirals shown in Fig. S4b.
3) Simulation Verification of Mode Purity To rigorously verify the mode behavior in the actual device geometry, we performed 3D-FDTD simulations of the adiabatic taper-to-taper structure used in the modulator. The simulation results indicate that the conversion efficiency from the fundamental mode to higher-order modes during transmission is approaching zero. This theoretically confirms that the fundamental mode launched from the quantum dot remains preserved throughout the short transmission distance of the routing device, effectively avoiding the coherence degradation concerns associated with multimode propagation.

Regarding the concern about TE-TM hybridization, we clarify that Lithium Tantalate (LT) exhibits relatively weak anisotropy compared to Lithium Niobate, and our eigenmode simulations of the specific shallow-etched geometry used here show no evidence of TE-TM mode hybridization. Furthermore, given that the quantum dots predominantly emit TE-polarized light and the device transmission distance is very short, depolarization effects due to fabrication imperfections (e.g., sidewall asymmetry) are minimal and do not compromise the device performance.

We performed the indistinguishability of the emitted single photons via a standard Hong-Ou-Mandel (HOM) two-photon interference experiment using phonon-assisted excitation. As shown in the revised Fig. 4(d), we deduce a high intrinsic photon indistinguishability of $V = 76.9 \pm 0.4\%$. The preservation of indistinguishability confirms that our shallow-etched LTOI waveguide robustly maintains the fundamental TE mode. We have added these HOM results and the related discussion to the revised main text and **Supplementary Information Note VIII**.

Manuscript Revision:
"We also characterized the indistinguishability of the emitted single photons via a standard Hong-Ou-Mandel (HOM) two-photon interference experiment using phonon-assisted excitation. The photons were sent into an unbalanced MZI with a path-length difference matching the excitation laser repetition period. As shown in Fig. 4(d), we recorded the coincidence counts for consecutive photons prepared in completely distinguishable (cross-polarized) and indistinguishable (co-polarized) states. The raw two-photon interference visibility is determined to be $V = 1 - A_{\parallel}/A_{\perp} = 76.9 \pm 0.4\%$ *(detailed in Supplementary Note VIII)."*

[Comment 2]
2) One concern that I have is that, even in theory, the coupling efficiency between the QD single-photon source and the LTOI waveguide is quite limited. In fact, while 80-90% might seem already a good value, for demanding applications such as fault-tolerant quantum computing, or the

demonstration of quantum computational advantage by boson sampling, one would like to work with a coupling efficiency as close as possible to 100%. Can the authors discuss alternative configurations enabling a higher coupling efficiency? (see, e.g., the configuration proposed in https://www.mdpi.com/2073-4352/11/11/1376)

Response:

We thank the reviewer for raising this important point regarding coupling efficiency and for providing the relevant reference. The reviewer points out that vertical (evanescent) coupling, as demonstrated in the cited work [*Wei et al., Crystals 2021*], is a powerful technique. However, the choice of coupling architecture—**End-to-End Coupling vs. Vertical Coupling**—strictly depends on the specific waveguide geometry and film thickness. We chose the end-to-end architecture for this work based on the following considerations:

1) **Optimization for the 600 nm LTOI Platform.** The reference provided employs a **300 nm** Lithium Niobate (LN) thin film. In such geometries, the optical mode is tightly confined vertically, making evanescent coupling to a top-loaded GaAs waveguide highly efficient. In contrast, our work utilizes a **600 nm thick** Lithium Tantalate (LT) film with a **300 nm etch depth** (ridge waveguide). In this thicker geometry, the optical mode center is located deeper within the LT layer. To quantitatively justify our choice, we performed a comprehensive simulation comparing the coupling efficiency and alignment tolerance of both schemes on our specific 600 nm platform. As shown in Figure R4, the end-to-end butt coupling achieves a significantly higher theoretical peak efficiency compared to the vertical scheme on this platform, and maintains high efficiency over a wider range of fabrication errors, whereas the vertical coupling efficiency drops more sharply due to the mode mismatch in the thick LT film.

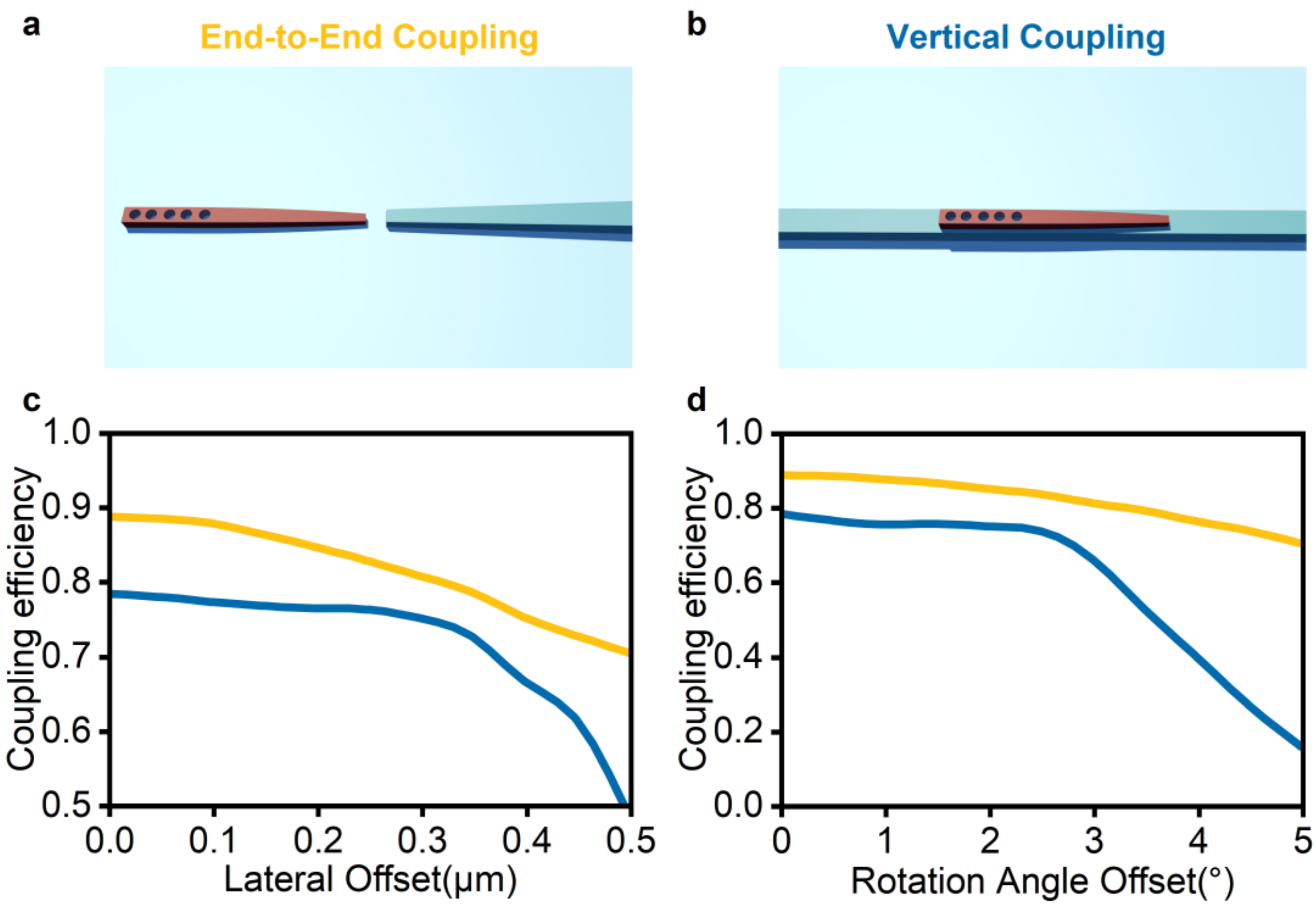

Figure R4. Simulation comparison of coupling efficiency and tolerance on the 600 nm LTOI platform. (a) Schematic illustration of the end-to-end coupling scheme. (b) Schematic illustration of the vertical coupling scheme. (c) Calculated coupling efficiency as a function of lateral offset

(misalignment). (d) Calculated coupling efficiency as a function of rotation angle offset (angular misalignment). In (c) and (d), the yellow and blue solid curves represent the simulated results for the end-to-end and vertical coupling schemes, respectively, demonstrating the higher alignment tolerance of the end-to-end approach.

2) Our group has also mastered the vertical coupling technique. As shown in Figure R5, in our very recent work [*Nat. Nanotechnol.* (2026). https://doi.org/10.1038/s41565-026-02132-1], we successfully transferred GaAs nanobeams onto a **300 nm thick (150 nm etched)** LN microring using the vertical coupling method.

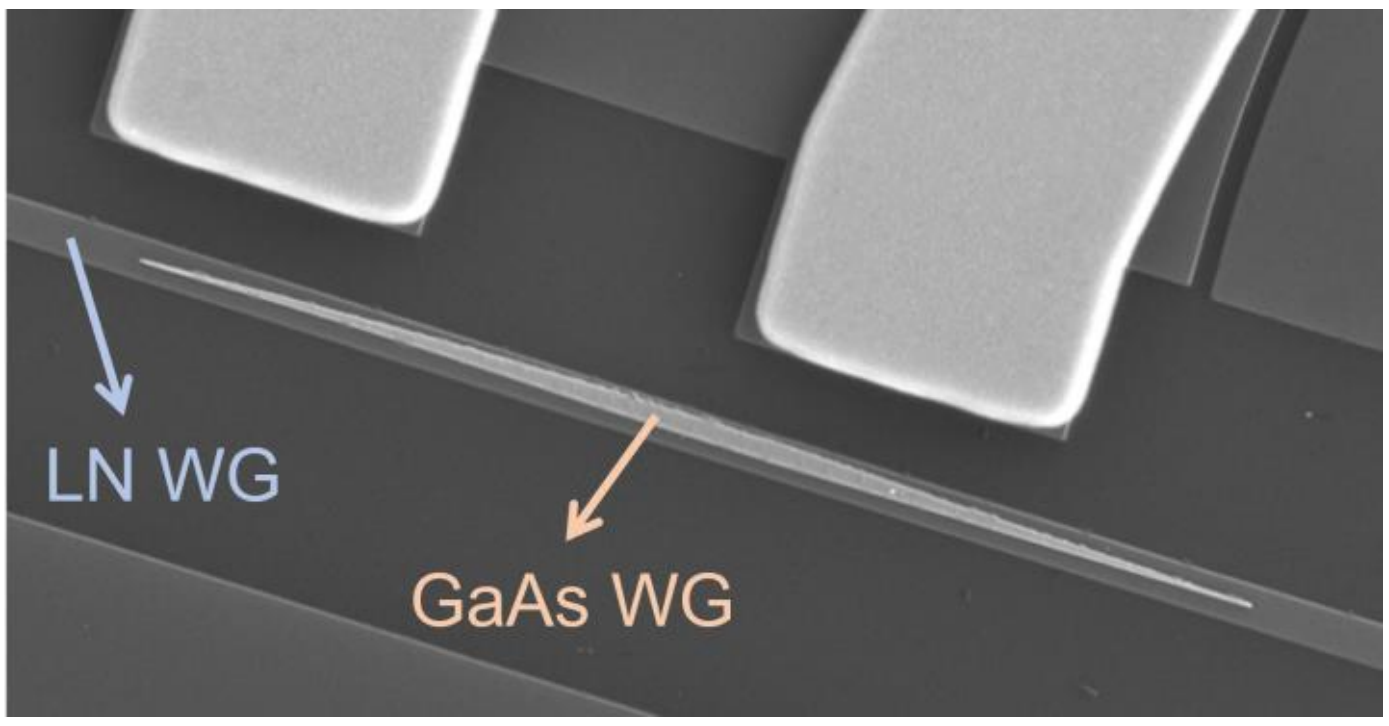

Figure R5. Our recent work employing vertical coupling on a 300 nm LNOI platform. We integrated GaAs waveguides onto a **300 nm thick** LN film using the vertical transfer printing method. On this thinner platform, vertical coupling is efficient and we achieved excellent alignment and device performance.

Therefore, our decision to use end-to-end coupling in this manuscript is a deliberate design choice optimized for the **600 nm LT ridge waveguide**. We believe that for this specific platform, the current architecture offers the best trade-off between coupling efficiency and mode matching.

Manuscript Revision:

"We deliberately employ this end-to-end coupling architecture rather than a vertical evanescent coupling scheme to optimally match our 600-nm-thick LTOI platform. This specific thickness is advantageous for accommodating the traveling-wave electrodes required for high-speed electro-optic modulation. Because the optical mode is confined deeper within this thicker LT layer, our planar butt-coupling is significantly more efficient and misalignment-tolerant than a vertical configuration. However, to achieve the near-unity coupling efficiencies required for highly demanding applications like fault-tolerant quantum computing, alternative configurations utilizing vertical coupling on thinner (e.g., 300 nm) ferroelectric films can be adopted, a methodology we have recently demonstrated successfully[45]."

45. Chen, Y. *et al.* On-chip non-Hermitian cavity quantum electrodynamics. *Nat. Nanotechnol.* https://doi.org/10.1038/s41565-026-02132-1 (2026).

[Comment 3]

3) In my opinion, the article fails to discuss precisely the various perspectives enabled by the integration of QD single-photon sources with fast electro-optic modulators + relevant references.

These could include, for instance, the realization of demultiplexed single-photon sources, fusion measurements for cluster state quantum computing, or frequency shifting of single photons. I would ask the authors to add a similar discussion in the article.

Response:

We thank the reviewer for this insightful suggestion. We agree that discussing the broader perspectives enabled by the integration of deterministic QD sources with fast electro-optic (EO) modulators is crucial for highlighting the impact of our platform.

In the revised "Discussion" section, we have added a comprehensive paragraph detailing several key applications enabled by our hybrid QD-LTOI platform:

1) **Demultiplexed single-photon sources:** Leveraging the fast switching speed of EO modulators to route photons into multiple spatial modes for scalable tasks like Boson sampling.
2) **Fusion measurements for cluster states:** Utilizing the deterministic generation of entangled photons combined with on-chip routing to facilitate fusion gates, which are essential for measurement-based quantum computing.
3) **Frequency shifting:** Integrating nonlinear functionalities to convert QD emission to telecom bands, bridging the gap between on-chip processing and fiber networks.

We have incorporated the relevant references as suggested to provide a balanced and forward-looking perspective.

Manuscript Revision:

" Beyond this initial demonstration, the hybrid integration of QD emitters with high-speed LTOI photonics opens several transformative avenues for scalable quantum information processing. First, the high-speed EO modulators enable the realization of demultiplexed single-photon sources[28]. By dynamically routing temporally separated photons from a single QD into different spatial modes via electro-optic switches, one can efficiently generate the multi-photon states required for large-scale Boson sampling[29,30,53,54]. Combined with low-loss optical delay lines to compensate for timing differences, this active demultiplexing approach offers a deterministic pathway to high-photon-number experiments. Second, this platform is well-suited for fusion-based quantum computing[55]. The deterministic generation of entangled photon strings from QDs, coupled with on-chip linear optical circuits, allows for fusion measurements to link small-scale entangled states into large-scale cluster states[56,57]. This capability paves the way for measurement-based quantum computing by creating the necessary large-scale entangled resources on a chip. Furthermore, single-photon frequency shifting presents another critical perspective. By leveraging the strong second-order nonlinearity ($\chi^{(2)}$) of the LTOI/LNOI platform, visible or near-infrared photons emitted by QDs can be coherently converted to the telecom C-band[58-60]. This frequency conversion is essential for interfacing high-performance on-chip quantum processors with long-distance fiber-optic quantum communication networks. "

28. Sund, P. I. *et al.* High-speed thin-film lithium niobate quantum processor driven by a solid-state quantum emitter. *Sci. Adv.* **9**, eadg7268 (2023).
29. Wang, Y. *et al.* Deterministic photon source interfaced with a programmable silicon-nitride integrated circuit. *npj Quantum Inf.* **9**, 94 (2023).
30. Maring, N. *et al.* A versatile single-photon-based quantum computing platform. *Nat. Photon.*

18, 603–609 (2024).
53. Wang, H. *et al.* Boson sampling with 20 input photons and a 60-mode interferometer in a 10^{14} -dimensional hilbert space. *Phys. Rev. Lett.* **123**, 250503 (2019).
54. Deng, Y.-H. *et al.* Gaussian boson sampling with pseudo-photon-number-resolving detectors and quantum computational advantage. *Phys. Rev. Lett.* **131**, 150601 (2023).
55. Bartolucci, S. *et al.* Fusion-based quantum computation. *Nat. Commun.* **14**, 912 (2023).
56. Schwartz, I. *et al.* Deterministic generation of a cluster state of entangled photons. *Science* **354**, 434–437 (2016).
57. Wu, B.-H., Alexander, R. N., Liu, S. & Zhang, Z. Quantum computing with multidimensional continuous-variable cluster states in a scalable photonic platform. *Phys. Rev. Res.* **2**, 023138 (2020).
58. Weber, J.H. *et al.* Two-photon interference in the telecom C-band after frequency conversion of photons from remote quantum emitters. *Nat. Nanotechnol.* **14**, 23–26 (2019).
59. Strobel, T. *et al.* Telecom-wavelength quantum teleportation using frequency-converted photons from remote quantum dots. *Nat. Commun.* **16**, 10027 (2025).
60. Ates, S. *et al.* Two-photon interference using background-free quantum frequency conversion of single photons emitted by an InAs quantum dot. *Phys. Rev. Lett.* **109**, 147405 (2012).

[Comment 4]

4) In the article, the QD is operated under non-resonant excitation. However, to achieve a single-photon purity and indistinguishability as high as possible, a resonant excitation scheme is required. Can the authors discuss alternative configuration for the GaAs waveguide that could enable to operate the QD under resonant excitation?

Response:

We thank the reviewer for this insightful comment. We agree that resonant excitation is indeed the standard for achieving the highest single-photon purity and indistinguishability. In this work, our primary focus is to demonstrate the first successful heterogeneous integration of GaAs QDs with LTOI photonics and verify the routing capability, for which non-resonant excitation serves as a robust proof-of-concept.

Regarding the reviewer's question on alternative configurations, our hybrid platform is inherently compatible with advanced waveguide designs that enable on-chip resonant excitation. We provide a detailed discussion of three specific strategies here to demonstrate the feasibility of future upgrades:

1) Polarization-based Rejection: As shown in Figure R6(a), Chanana et al. utilized the high modal birefringence of high-aspect-ratio Si_3N_4 waveguides to achieve on-chip pump suppression. By exciting the QD with TM-polarized light—a mode not supported by the ultra-low-loss waveguide—the pump scatter is intrinsically rejected. Meanwhile, the TE-polarized QD fluorescence couples into the circuit, enabling the observation of resonance fluorescence with a 25 dB signal-to-background ratio. [*Nat. Commun. 13, 7693 (2022)*].
2) Dual-Mode Waveguide Filtering: Alternatively, we can employ a multimode waveguide section where the pump is coupled to a higher-order mode and the QD emits into the fundamental mode. As illustrated in Figure R6(b), a subsequent photonic crystal filter or tapered waveguide selectively blocks the pump mode, enabling "plug-and-play" resonant operation [*Uppu et al., Nat. Commun. 11, 3782 (2020)*].

3) Elliptical Bragg Resonators: Our transfer printing method supports the integration of QDs within elliptical Bragg resonators (Figure R6(c)). The geometric asymmetry lifts the degeneracy of cavity modes, allowing the pump (ω_1) and signal (ω_2) to be spectrally and spatially separated without external filtering [*Chen et al., Photon. Res. 10, 2066 (2022)*].

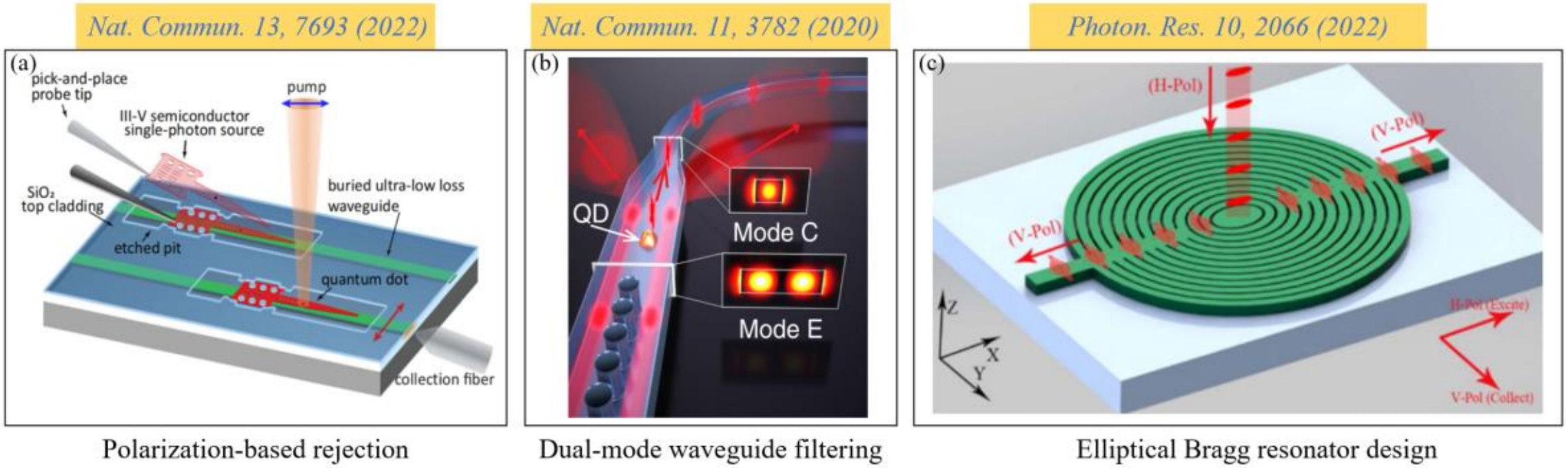

Figure R6. Proven on-chip resonant excitation schemes compatible with our hybrid platform. (a) Control of the pump beam polarization (indicated by the blue arrow) allows resonant QD excitation with minimal pump scattering into the Si3N4 waveguide, allowing observation of resonance fluorescence coupled to the transverse-electric (TE) polarized mode (represented by the red arrow). (b) The resonant pump laser in the first-order waveguide mode excites the emitter and is subsequently squeezed out of the waveguide in the taper section. The QD emission into the fundamental mode of the waveguide is collected efficiently and guided. The photonic crystal acts a mirror for the fundamental mode, thereby enabling the directional out-coupling of the QD signal. (c) The pulsed resonance excitation laser with H-Pol excites the solid-state quantum emitters embedded in the central elliptical disk of the resonator. Only the excited emitter matching the V-Pol resonating mode can emit V-Pol single-photon streams efficiently due to polarization dependent Purcell enhancement.

These configurations confirm that our current hybrid integration approach does not impose any fundamental limitations on achieving resonance fluorescence. We believe this detailed discussion clarifies the pathway toward high-indistinguishability sources on the LTOI platform.

Manuscript Revision:

"While the QDs in this work were operated under non-resonant excitation for proof-of-concept, achieving higher single-photon purity and indistinguishability will benefit from resonant excitation schemes. Our platform is compatible with several on-chip resonant excitation strategies to suppress pump scattering, such as using high-aspect-ratio Si_3N_4 waveguides for polarization-based rejection[36], employing dual-mode waveguide filtering to selectively attenuate the higher-order pump mode via tapered sections[46], or integrating elliptical Bragg resonators to separate the pump and signal through broken geometric degeneracy[47]. These alternative configurations confirm that our hybrid integration approach provides a viable pathway toward high-performance, indistinguishable single-photon sources on the LTOI platform."

36. Chanana, A. *et al.* Ultra-low loss quantum photonic circuits integrated with single quantum emitters. *Nat. Commun.* **13**, 7693 (2022).

46. Uppu, R. *et al.* On-chip deterministic operation of quantum dots in dual-mode waveguides for

a plug-and-play single-photon source. *Nat. Commun.* **11**, 3782 (2020).
47. Chen, X., Su, R., Liu, J., Li, J. & Wang, X.-H. Scalable and highly efficient approach for an on-chip single-photon source. *Photon. Res.* **10**, 2066 (2022).

[Comment 5]

5) A relevant article (published only very recently) which is to not cited in the manuscript, is: https://www.nature.com/articles/s41563-025-02398-1 . In particular, this article demonstrates that, by leveraging the piezoelectric properties of lithium niobate, it is possible to spectrally tune individual QDs and achieve indistinguishable emission between separated single-photon sources. I think this is a very important perspective which deserves to be discussed.

Response:

We are grateful to the reviewer for bringing this highly relevant work to our attention. We have added a discussion in the revised manuscript regarding the practical implementation of on-chip tuning. We highlight two prominent, circuit-compatible solutions:

1) The Quantum-Confined Stark Effect (QCSE): Previous work by Aghaeimeibodi et al. [*Appl. Phys. Lett. 114, 071105 (2019)*] has demonstrated large wavelength tunability of individual quantum dot emitters.
2) Piezoelectric Strain Tuning: Leveraging the piezoelectric properties of thin-film lithium niobate (TFLN), Wang et al. [*Nat. Mater. 24, 1898–1905 (2025)*] recently achieved a local, reversible strain-tuning technique. Notably, they demonstrated the scalability of this mechanism by independently tuning the emission spectra of 20 deterministic single-photon sources on a single chip.

These advancements provide a clear roadmap for the practical realization of multi-array architecture. We have updated the manuscript to reflect this discussion.

Manuscript Revision:

" Figure 1a illustrates the future architecture of this chip platform, where arrays of QDs can be integrated on-chip. A challenge to realizing this scalable multi-array architecture is the inherent spectral randomness of self-assembled QDs. The spectral inhomogeneity can be addressed by integrating circuit-compatible local tuning mechanisms. For instance, individual emitters can be tuned using local electric fields via the Quantum-Confined Stark Effect[41]. Furthermore, the intrinsic piezoelectric properties of ferroelectric platforms enable dynamic and reversible local strain tuning, which can independently tune the spectra of individual single-photon sources over a large range, offering a practical pathway to scalable on-chip quantum networks[42]."

41. Aghaeimeibodi, S., Lee, C.-M., Buyukkaya, M. A., Richardson, C. J. K. & Waks, E. Large stark tuning of InAs/InP quantum dots. *Appl. Phys. Lett.* 114, 071105 (2019).
42. Wang, X. *et al.* Large-scale quantum dot–lithium niobate hybrid integrated photonic circuits enabling on-chip quantum networking. *Nat. Mater.* 24, 1898–1905 (2025).

[Comment 6]

6) Lines 48-50:

“Notably, the successful integration of cryogenic-compatible LNOI circuits with superconducting

nanowire single-photon detectors (SNSPDs) has underscored their immense potential for quantum technologies (7,8)."

Ref. 8 doesn't demonstrate the integration of LNOI circuits with SNSPDs. More relevant articles that the authors might want to cite in addition to Ref. 7, are: https://pubs.aip.org/aip/apl/article/116/15/151102/38104/Lithium-niobate-on-insulator-waveguide-integrated , https://pubs.acs.org/doi/10.1021/acs.nanolett.3c02324 , https://pubs.acs.org/doi/full/10.1021/acsphotonics.3c01628 .

Response:

We sincerely thank the reviewer for the rigorous review and constructive comments, which are crucial for improving the accuracy and quality of our manuscript. We apologize for the oversight regarding the original Reference [8] (*Nat. Commun. 15, 10459 (2024)*). As correctly pointed out by the reviewer, this work focuses on efficient couplers and high-bandwidth phase modulators on the LNOI platform, rather than demonstrating the direct integration of LNOI circuits with superconducting nanowire single-photon detectors (SNSPDs). We have corrected this in the revised manuscript by citing the three highly relevant articles suggested by the reviewer.

Manuscript Revision:

" Notably, the successful integration of cryogenic-compatible LNOI circuits with superconducting nanowire single-photon detectors (SNSPDs) has underscored their immense potential for quantum technologies[7–10]. "

7. Lomonte, E. *et al.* Single-photon detection and cryogenic reconfigurability in lithium niobate nanophotonic circuits. *Nat. Commun.* **12**, 6847 (2021).
8. Sayem, A. A., Cheng, R., Wang, S. & Tang, H. X. Lithium-niobate-on-insulator waveguide-integrated superconducting nanowire single-photon detectors. *Appl. Phys. Lett.* **116**, 151102 (2020).
9. Prencipe, A. *et al.* Wavelength-sensitive superconducting single-photon detectors on thin film lithium niobate waveguides. *Nano Lett.* **23**, 9748–9752 (2023).
10. Colangelo, M. *et al.* Molybdenum silicide superconducting nanowire single-photon detectors on lithium niobate waveguides. *ACS Photonics* **11**, 356–361 (2024).

[Comment 7]

7) Lines 52-55:

"Moreover, the LTOI platform exhibits advantages such as expanded transparent window (0.28-5.5um), decreased optical anisotropy, stable operation at low frequency drive and high optical power, which renders it beneficial for building photonic integrated 54 circuits (PICs) at shorter wavelengths (9-11)."

Here one could argue that: i) since the emission wavelength of QDs is 900 nm, which is well within the transparency range of LN, having an expanded transparency window is not much useful; ii) while the DC bias drift of LNOI is a big issue at room temperature, it gets strongly suppressed, and becomes almost negligible, at cryogenic temperatures (see https://www.nature.com/articles/s41467-021-27205-8 and also https://opg.optica.org/ol/fulltext.cfm?uri=ol-50-17-5398)

Response:

We thank the reviewer for this insightful critique. We acknowledge that for QDs emitting at ~900 nm, the extended UV transparency window of LTOI (down to 0.28 μm) is not a critical advantage compared to LNOI. And we appreciate the reviewer pointing out the recent literature (*Nat. Commun. 12, 6847, 2021* and *Opt. Lett. 50, 5398, 2025*), which convincingly demonstrates that the DC drift issues often observed in LNOI at room temperature are effectively suppressed at cryogenic temperatures.

In light of this, we agree that emphasizing the specific advantages of LTOI over LNOI regarding transparency range and DC stability is not appropriate in the context of this specific work. Therefore, in the revised manuscript, we have reframed this section. Instead of comparing LTOI against LNOI, we now highlight the general suitability of ferroelectric thin-film platforms (including LTOI) for cryogenic quantum photonics, focusing on their shared benefits such as low propagation loss and effective electro-optic reconfiguration.

Manuscript Revision:

"Moreover, ferroelectric thin-film platforms like LT offer exceptional properties for integrated quantum photonics, including stable electro-optic response at cryogenic temperatures and low optical propagation losses at the emission wavelength of the quantum emitters (~900 nm)[11–14]. These characteristics are crucial for building low-loss and reconfigurable photonic integrated circuits that operate effectively with on-chip quantum emitters."

11. Wang, C. *et al.* Lithium tantalate photonic integrated circuits for volume manufacturing. *Nature* **629**, 784–790 (2024).
12. Yu, J. *et al.* Tunable and stable micro-ring resonator based on thin-film lithium tantalate. *APL Photonics* **9**, 036115 (2024).
13. Wang, H. *et al.* Thin-film lithium tantalate modulator operating at high optical power. *ACS Photonics* acsphotonics.5c00159 (2025).
14. Huang, F. *et al.* Toward large‐scale photonic chips using low‐anisotropy thin‐film lithium‐tantalate. *Adv. Sci.* **12**, 2410345 (2025).

[Comment 8]

8) Lines 261-262:

"This analysis yielded an on-chip extraction efficiency of 29.7%, representing the fraction of emitted photons successfully coupled into and guided by the hybrid GaAs-LT waveguide system".

Maybe I didn't understand well what the authors mean, but 29.7%, according to the Supplementary Material, shouldn't be the extraction efficiency at the first objective? I would not call this "on-chip" extraction efficiency since, according to the Supplementary, it also takes into account the imperfect overlap of the waveguide mode with the objective (or with the fiber placed after the objective, see the next comment).

Response:

We thank the reviewer for this accurate correction. We agree that the term "on-chip extraction efficiency" was imprecise, as the measured value of 29.7% represents the photon flux collected by the measurement setup, which inherently includes the extraction loss at the chip-to-free-space interface.

In the revised manuscript, we have renamed this metric to "extraction efficiency at the first objective". This value represents the fraction of emitted photons that are successfully guided through the hybrid waveguide and collected by the objective lens.

We further compared this experimental value (29.7%) with our detailed loss budget simulation. The theoretical efficiency at the first objective is calculated to be $\eta_{sim} \approx 46\%$, which accounts for:

1) Source-to-Waveguide Coupling ($\eta_{QD-LT} \approx 0.80$): This aggregate efficiency represents the probability of a photon emitted by the quantum dot being successfully coupled into the LTOI waveguide mode. It is determined by the product of two sub-processes: the coupling of the quantum dot's spontaneous emission into the GaAs nanobeam fundamental mode ($\beta - factor \approx 0.82$) and the subsequent adiabatic mode transfer from the GaAs nanobeam to the LTOI waveguide ($\eta_{mode} \approx 0.98$). The high transfer efficiency is achieved through the optimized inverted taper design shown in Figure R7. We clarify that the value ">85%" mentioned in the main text refers specifically to the structural mode transfer efficiency (η_{mode}), while the total source coupling efficiency ($\eta_{QD-LT} \approx 0.80$) accounts for the intrinsic $\beta - factor$.

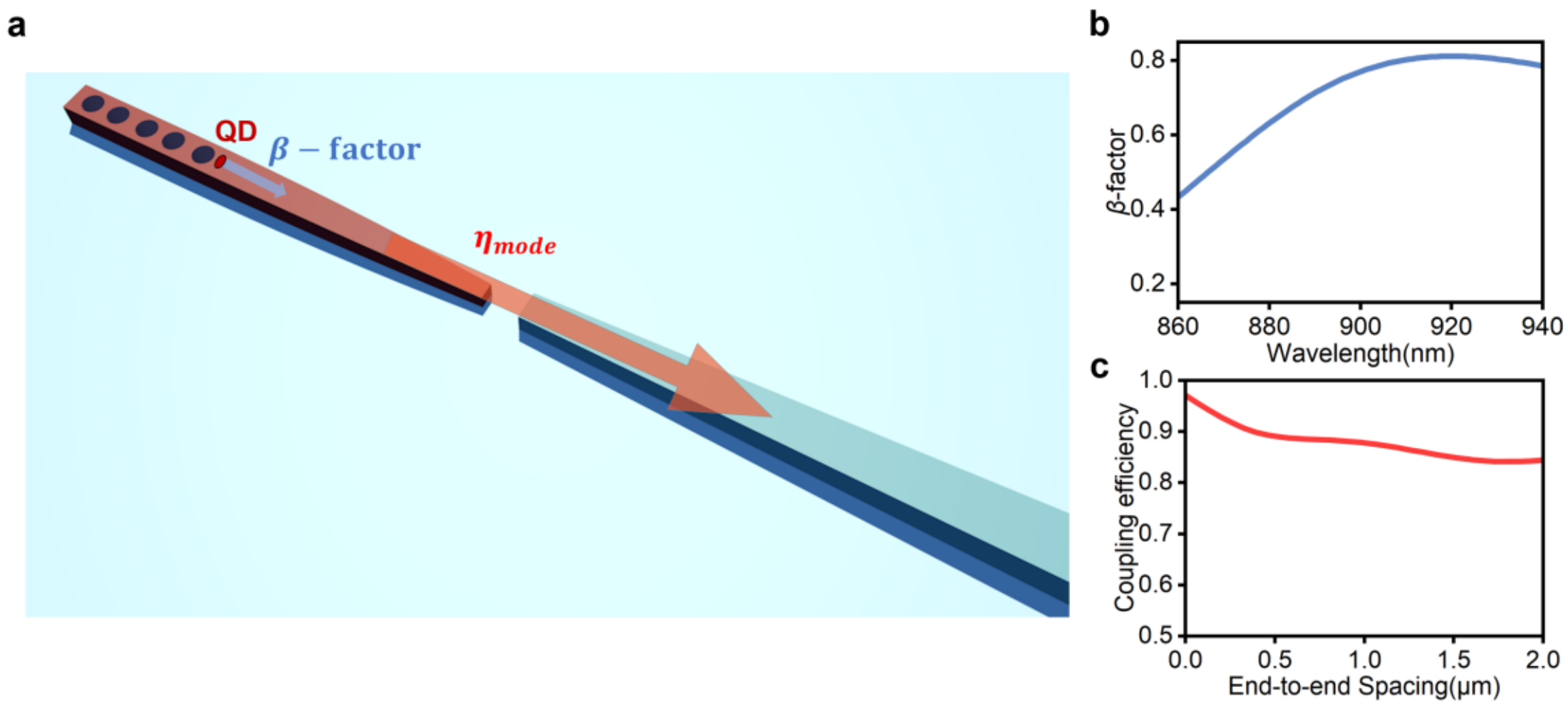

Figure R7. Simulation analysis of the source-to-waveguide coupling efficiency. (a) Schematic illustration of the hybrid interface employing an inverted taper design. The total probability of a photon coupling into the LTOI waveguide is determined by the product of the quantum dot's spontaneous emission coupling factor ($\beta - factor$) and the structural mode transfer efficiency (η_{mode}). (b) Calculated $\beta - factor$ describing the coupling of the quantum dot's spontaneous emission into the GaAs nanobeam fundamental mode. At the wavelength of 920 nm, the simulation yields a $\beta - factor \approx 0.82$. (c) Calculated adiabatic mode transfer efficiency (η_{mode}) as a function of the end-to-end spacing between the waveguides. For the optimized configuration used in our experiment, the simulation demonstrates a high structural transfer efficiency of $\approx 98\%$.

2) Propagation Efficiency ($\eta_{trans} \approx 1$): Given the short device length and the optimized theoretical design, we approximate the transmission efficiency as unity.

3) Device Insertion Loss ($\eta_{MZI} \approx 0.92$): The single photons pass through two Multi-Mode Interference (MMI) couplers within the Mach-Zehnder Interferometer (MZI). Simulations indicate $\sim 4\%$ loss per MMI ($0.96 \times 0.96 \approx 0.92$).

4) Objective Collection Efficiency ($\eta_{obj} \approx 0.63$): Finally, the light out-couples from the

waveguide facet. Due to the optical confinement in the LT ridge waveguide, the beam diverges significantly. As shown in Figure R8(a), our FDTD simulation indicates that the objective lens with NA=0.42 (marked by the red circle) collects approximately 63% of the total emitted energy.

Multiplying these simulated components yields a theoretical extraction efficiency of:

$$\eta_{sim} = \eta_{QD-LT} \times \eta_{trans} \times \eta_{MZI} \times \eta_{obj} \approx 0.80 \times 1 \times 0.92 \times 0.63 \approx 0.46$$

The measured efficiency (29.7%) is lower than the theoretical limit (46%), a difference we attribute to experimental constraints and fabrication imperfections not captured in ideal models—specifically, scattering at the hybrid interface and waveguide sidewalls, non-ideal reflectivity of the GaAs mirror, and potential misalignment. However, these are addressable engineering challenges. Our analysis confirms the high intrinsic efficiency of the hybrid interface, suggesting that future optimizations in etching quality and end-facet design could significantly boost overall performance.

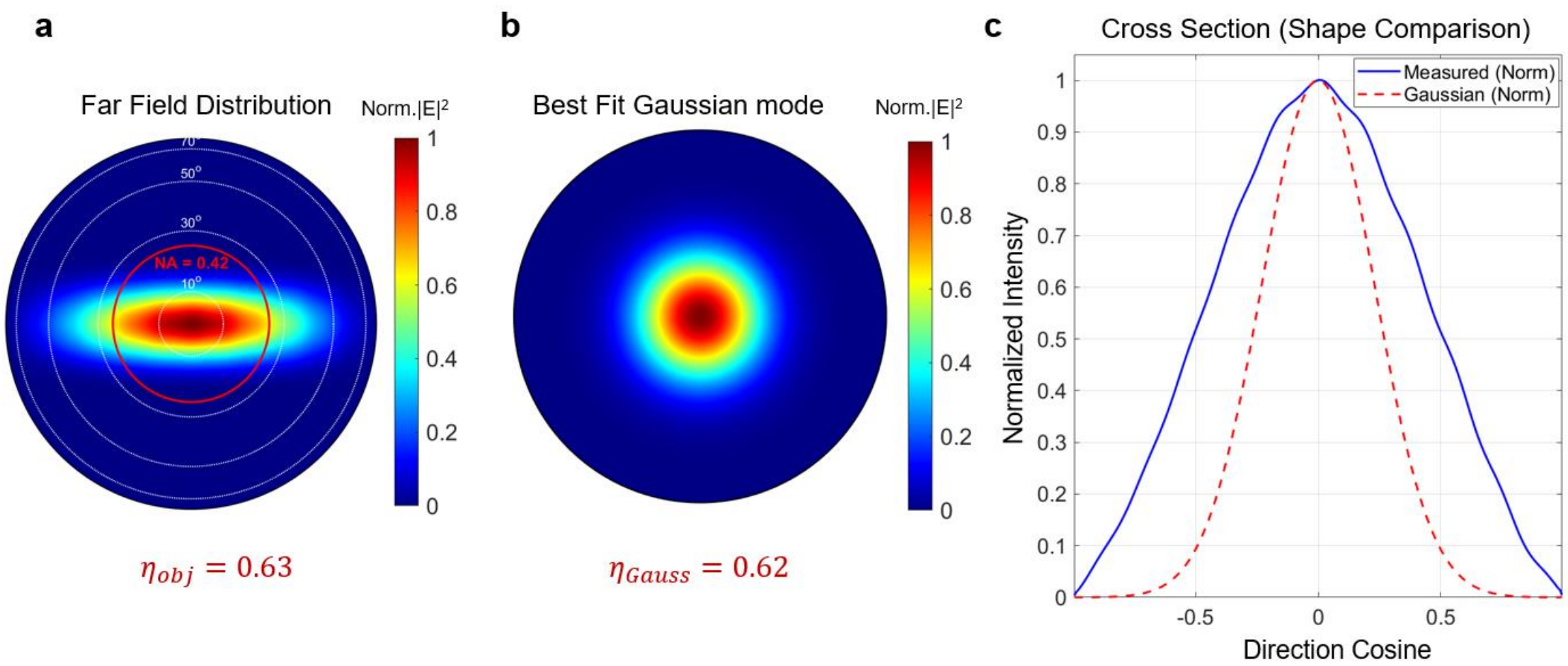

Figure R8. Far-field emission characteristics and fiber coupling analysis. (a) Simulated far-field radiation intensity distribution ($|E|^2$) emitted from the LTOI waveguide facet. The red dashed circle delineates the acceptance cone of the objective lens (NA=0.42), which collects approximately 63% of the total radiated power. (b) The fundamental Gaussian mode profile that provides the maximum overlap with the emitted far-field pattern. The calculated overlap integral yields a mode matching efficiency of $\eta_{Gauss} \approx 0.62$. (c) Comparison of the cross-sectional intensity profiles between the waveguide far-field emission (solid line) and the fitted Gaussian mode (dashed line). The deviation between the two curves visually illustrates the mode mismatch responsible for energy loss.

In the revised **Supplementary Information Note VII**, we have provided a detailed component-by-component breakdown based on our updated FDTD simulations and experimental parameters.

Manuscript Revision:

"This analysis yielded an extraction efficiency at the first objective of 29.7%, representing the fraction of emitted photons successfully guided by the hybrid GaAs-LT waveguide system and collected by the objective lens."

[Comment 9]

9) One question strictly related to the previous one. Supplementary Materials, lines 158-160:

“However, the mode overlap between the output field of the LT waveguide and the Gaussian mode accepted by the NA=0.42 objective was only 0.39, due to the lack of structural optimization at the waveguide output facet.”

I am a bit confused by this part, but I would be prone to think that, if the NA of the objective is sufficiently high, all the light should be collected by the objective independently from the exact shape of the waveguide mode. On the other hand, one should certainly take into account the reduced overlap of the collected light with the fiber placed after the objective. But perhaps this is just what the authors mean.

Response:

We appreciate the reviewer’s deep insight into the coupling mechanism. A high NA objective typically collects most of the light. In our analysis presented in the Supplementary Information, the factor of **0.39** is not solely the collection efficiency of the objective lens, but the product of the **geometric collection efficiency** and the **fiber mode overlap**.

1) **Geometric Collection ($\eta_{obj} \approx 0.63$):** Even with NA=0.42, the objective does not collect "all" the light. Due to the tight optical confinement in the sub-wavelength LTOI ridge waveguide, the output beam diverges significantly. As shown in Figure R8(a), our FDTD far-field simulations show that only **~63%** of the energy falls within the collection cone of the objective.
2) **Fiber Mode Overlap ($\eta_{Guass} \approx 0.62$):** Since the collected light is coupled into a single-mode fiber (SMF) via a collimator, we must account for the mode mismatch. As shown in Figure R8(b), the distorted beam profile from the waveguide ridge results in a maximum overlap of **~62%** with the fundamental Gaussian mode of the fiber collimator.

Therefore, the value of 0.39 cited in the original text corresponds to the total fiber coupling efficiency:

$$\eta_{total} = \eta_{obj} \times \eta_{Gauss} = 0.63 \times 0.62 \approx 0.39$$

[Comment 10]

10) Lines 281-282:

“The butt-coupled waveguide architecture achieves >85% mode coupling efficiency”.

According to the supplementary, it shouldn’t be slightly lower than 80%?

Response:

We apologize for the confusion caused by the ambiguous definitions.

1) The value **">85%"** in the main text referred specifically to the **inter-waveguide mode transfer efficiency** (η_{mode}) between the GaAs nanobeam and the LTOI waveguide. Our FDTD simulations indicate this transfer efficiency is as high as **~98%** (see Fig. R7(b)), so ">85%" was a conservative claim for the coupler design itself.
2) The value **"~80%"** in the Supplementary Information refers to the **total source-to-waveguide coupling efficiency** (η_{QD-LT}), which is the product of the quantum dot's β-factor (82%) and the mode transfer efficiency ($\eta_{mode} \approx 0.98$).

Manuscript Revision:

“The butt-coupled waveguide architecture can achieve ~80% total source-to-waveguide coupling efficiency, enabling the first hybrid quantum photonic chip capable of cryogenic single-photon

routing, a critical milestone for on-chip quantum information processing."

[Comment 11]

11) Lines 283-289:

"Looking forward, full-scale quantum information processing demands ultra-low-loss delay lines (~1 dB/m) and integrated superconducting detectors (e.g., SNSPDs), both challenged by LT's current propagation loss and fabrication complexity (39). Heterogeneous integration with SiN waveguides (~1dB/m loss) (40) and monolithic SNSPD fabrication on LN substrates (41,42) provide viable solutions."

I have two observations: i) despite the fact that prop loss down to 1 dB/m have been also demonstrated on LNOI (and I would assume the same should be possible with LTOI) integrating everything on-chip is probably hopeless, and one should also efficiently couple the waveguides with optical fibers for the realization of very long delay lines; ii) Ref. 41 was already cited as Ref. 7, while Ref. 42 doesn't demonstrate the integration of SNSPDs with LNOI waveguides (see comment 6 for more relevant references). Please carefully check if similar issues are present with also other references within the manuscript.

Response:

We thank the reviewer for this constructive perspective. We agree that for the realization of very long delay lines, relying solely on on-chip integration is limited by footprint constraints, making efficient fiber-to-chip coupling an indispensable requirement.

To address this, our platform is designed to be compatible with high-efficiency fiber interfaces. For instance, recent advances in multi-layer taper structures combined with localized substrate undercutting have demonstrated fabrication-tolerant coupling with losses as low as ~0.25 dB per facet [*Chen et al., APL Photon. 9, 116111 (2024)*]. By merging high-speed LTOI circuits with both ultra-low-loss planar waveguides (such as SiN [*Cai et al., Nat Commun (2026).* https://doi.org/10.1038/s41467-026-69769-3] or Ge-silica [*Chen et al., Nature 649, 338–344 (2026).* https://doi.org/10.1038/s41586-025-09889-w]) and efficient fiber-packaging technologies, we believe a practical and scalable solution for long-range quantum photonic delay lines can be achieved.

For referencing inaccuracies, we sincerely thank the reviewer for the highly careful reading and for pointing out. We have removed the duplicate citation and replaced Ref. 42 with the more relevant references. Furthermore, we have conducted a comprehensive and thorough check of all references and their corresponding citations throughout the entire manuscript.

[Comment 12]

12) Lines 290-293:

"As ultra-low-loss photonics and cryogenic detector technologies mature, this convergence enables fully integrated quantum processors for boson sampling, quantum walks, and entanglement distribution—unlocking optical quantum computing's potential within 292 near-term experimental reach."

Please discuss more precisely the perspectives enabled by the integration of QDs with fast electro-optic modulators (see also comment 3) and add some relevant references.

Response:

We thank the reviewer for this suggestion. As noted in our Response to **Comment 3**, we agree that discussing specific perspectives such as demultiplexing, fusion measurements, and frequency shifting is essential to highlight the potential of our QD-LTOI platform. In the revised "Discussion" section, we have added a comprehensive paragraph detailing several key applications enabled by our hybrid QD-LTOI platform.

Manuscript Revision:

" Beyond this initial demonstration, the hybrid integration of QD emitters with high-speed LTOI photonics opens several transformative avenues for scalable quantum information processing. First, the high-speed EO modulators enable the realization of demultiplexed single-photon sources[28]. By dynamically routing temporally separated photons from a single QD into different spatial modes via electro-optic switches, one can efficiently generate the multi-photon states required for large-scale Boson sampling[29,30,53,54]. Combined with low-loss optical delay lines to compensate for timing differences, this active demultiplexing approach offers a deterministic pathway to high-photon-number experiments. Second, this platform is well-suited for fusion-based quantum computing[55]. The deterministic generation of entangled photon strings from QDs, coupled with on-chip linear optical circuits, allows for fusion measurements to link small-scale entangled states into large-scale cluster states[56,57]. This capability paves the way for measurement-based quantum computing by creating the necessary large-scale entangled resources on a chip. Furthermore, single-photon frequency shifting presents another critical perspective. By leveraging the strong second-order nonlinearity ($\chi^{(2)}$) of the LTOI/LNOI platform, visible or near-infrared photons emitted by QDs can be coherently converted to the telecom C-band[58-60]. This frequency conversion is essential for interfacing high-performance on-chip quantum processors with long-distance fiber-optic quantum communication networks."

28. Sund, P. I. *et al.* High-speed thin-film lithium niobate quantum processor driven by a solid-state quantum emitter. *Sci. Adv.* **9**, eadg7268 (2023).
29. Wang, Y. *et al.* Deterministic photon source interfaced with a programmable silicon-nitride integrated circuit. *npj Quantum Inf.* **9**, 94 (2023).
30. Maring, N. *et al.* A versatile single-photon-based quantum computing platform. *Nat. Photon.* **18**, 603–609 (2024).
53. Wang, H. *et al.* Boson sampling with 20 input photons and a 60-mode interferometer in a 10^{14}-dimensional hilbert space. *Phys. Rev. Lett.* **123**, 250503 (2019).
54. Deng, Y.-H. *et al.* Gaussian boson sampling with pseudo-photon-number-resolving detectors and quantum computational advantage. *Phys. Rev. Lett.* **131**, 150601 (2023).
55. Bartolucci, S. *et al.* Fusion-based quantum computation. *Nat. Commun.* **14**, 912 (2023).
56. Schwartz, I. *et al.* Deterministic generation of a cluster state of entangled photons. *Science* **354**, 434–437 (2016).
57. Wu, B.-H., Alexander, R. N., Liu, S. & Zhang, Z. Quantum computing with multidimensional continuous-variable cluster states in a scalable photonic platform. *Phys. Rev. Res.* **2**, 023138 (2020).

58. Weber, J.H. *et al.* Two-photon interference in the telecom C-band after frequency conversion of photons from remote quantum emitters. *Nat. Nanotechnol.* **14**, 23–26 (2019).
59. Strobel, T. *et al.* Telecom-wavelength quantum teleportation using frequency-converted photons from remote quantum dots. *Nat. Commun.* **16**, 10027 (2025).
60. Ates, S. *et al.* Two-photon interference using background-free quantum frequency conversion of single photons emitted by an InAs quantum dot. *Phys. Rev. Lett.* **109**, 147405 (2012).

Reviewer #3:

Editor-specific Questions:

[Comment 1]

1. What are the noteworthy results?

- The authors integrate of a single photon source comprised of an indium-arsenide quantum dot into a lithium tantalate photonic platform with high-speed configurability.
- The authors show a relatively high, but not record, photon extraction efficiency whilst integrating an additional component into the single photon source (the modulator) at cryogenic temperatures.

Response:

We thank the reviewer for the summary and the positive assessment. In the following, we provide point to point response to improve the quality of the work.

[Comment 2]

2. Will the work be of significance to the field and related fields? How does it compare to the established literature? If the work is not original, please provide relevant references.

The work has significance as a technology demonstrator, specifically for hybrid hardware platforms for integrated photonics with deterministic single photon sources. For the broader photonic quantum processor, and quantum computing fields in general, with the limited demonstration presented here, the significance is still unclear.

- Without any demonstration of source indistinguishability, photon interference, or gate operations, the link to being a suitable hardware platform for quantum photonic processors is tenuous at best.

Response:

We sincerely thank the reviewer for pointing out this critical aspect. We agree that a fully functional quantum photonic processor requires the seamless monolithic integration of deterministic single-photon sources, complex gate operations, long delay lines, and highly efficient detectors. However, achieving this "full on-chip processing" represents a massive engineering challenge that the entire field is currently striving to overcome. To realize a complete on-chip quantum processor, several stringent prerequisites need to be met simultaneously. For the quantum sources, achieving the near-unity indistinguishability may require the application of local electric fields to purify the electromagnetic environment and suppress charge noise. And the subsequent deterministic gate operations—such as the fusion gates currently being advanced in continuous-variable architectures—require perfectly balanced interferometers and high-strength interactions. Furthermore, multi-photon synchronization strictly relies on macroscopic, ultra-low-loss delay lines to buffer photons without significant signal degradation or massive footprint overhead. Finally, the integration of superconducting nanowire single-photon detectors (SNSPDs) involves complex cryogenic packaging that must not introduce additional optical loss to the active waveguide circuits.

While preliminary on-chip two-photon interference has been recently demonstrated in a hybrid platform ([*Nat. Mater. 24, 1898–1905 (2025)*]), simultaneously integrating optimal materials and structures for all these functions onto a single chip remains a universally recognized bottleneck. In our current experimental framework, constrained by material limitations—the absence of a p-i-n diode structure in our transferred GaAs membranes—and current fabrication limits regarding ultra-

low-loss macroscopic delay lines, we acknowledge that a complete on-chip interference test is currently out of reach. Nevertheless, to quantitatively evaluate the potential of our hardware for quantum processing applications, we have now performed an off-chip Hong-Ou-Mandel (HOM) two-photon interference experiment using phonon-assisted excitation. Using an unbalanced Mach-Zehnder interferometer with a delay matching the laser repetition period, we measured the two-photon interference fringes under both co-polarized (indistinguishable) and cross-polarized (distinguishable) configurations. By comparing the integrated areas of the central peaks ($A_{\parallel}$and $A_{\perp}$), we extracted an HOM interference visibility of $V = 1 - A_{\parallel}/A_{\perp} = 76.9 \pm 0.4\%$. This high degree of indistinguishability clearly demonstrates the suitability and high potential of our hybrid platform. We have updated Figure 4 in the revised manuscript with the HOM interference data and added detailed discussions in both the main text and the Supplementary Information Note VIII.

Most importantly, we emphasize that the primary significance of this work lies in the first demonstration of deterministic, high-speed on-chip routing of single photons at cryogenic temperatures. Previous hybrid integration efforts have largely been limited to passive coupling or static transmission. By harnessing the strong electro-optic properties of lithium tantalate, we provide the crucial active routing capability necessary for dynamic demultiplexing. This cryogenic reconfigurability is a foundational stepping stone that bridges the gap toward the fully integrated scalable quantum photonic networks discussed above.

Manuscript Revision:

"We also characterized the indistinguishability of the emitted single photons via a standard Hong-Ou-Mandel (HOM) two-photon interference experiment using phonon-assisted excitation. The photons were sent into an unbalanced MZI with a path-length difference matching the excitation laser repetition period. As shown in Fig. 4(d), we recorded the coincidence counts for consecutive photons prepared in completely distinguishable (cross-polarized) and indistinguishable (co-polarized) states. The raw two-photon interference visibility is determined to be $V = 1 - A_{\parallel}/A_{\perp} = 76.9 \pm 0.4\%$ *(detailed in Supplementary Note VIII)."*

- A comparison should be made to other papers that have looked at integrating deterministic single photon sources with fast reconfigurability to compare key parameters.

Response:

We thank the reviewer for this valuable suggestion. To comprehensively benchmark our platform against the state-of-the-art, we have expanded our comparison to include the suggested works, covering the primary modulation mechanisms: **Thermo-optic**, **MEMS**, **Acousto-optic (SAW)**, and **Electro-optic (GaAs & LTOI)** schemes.

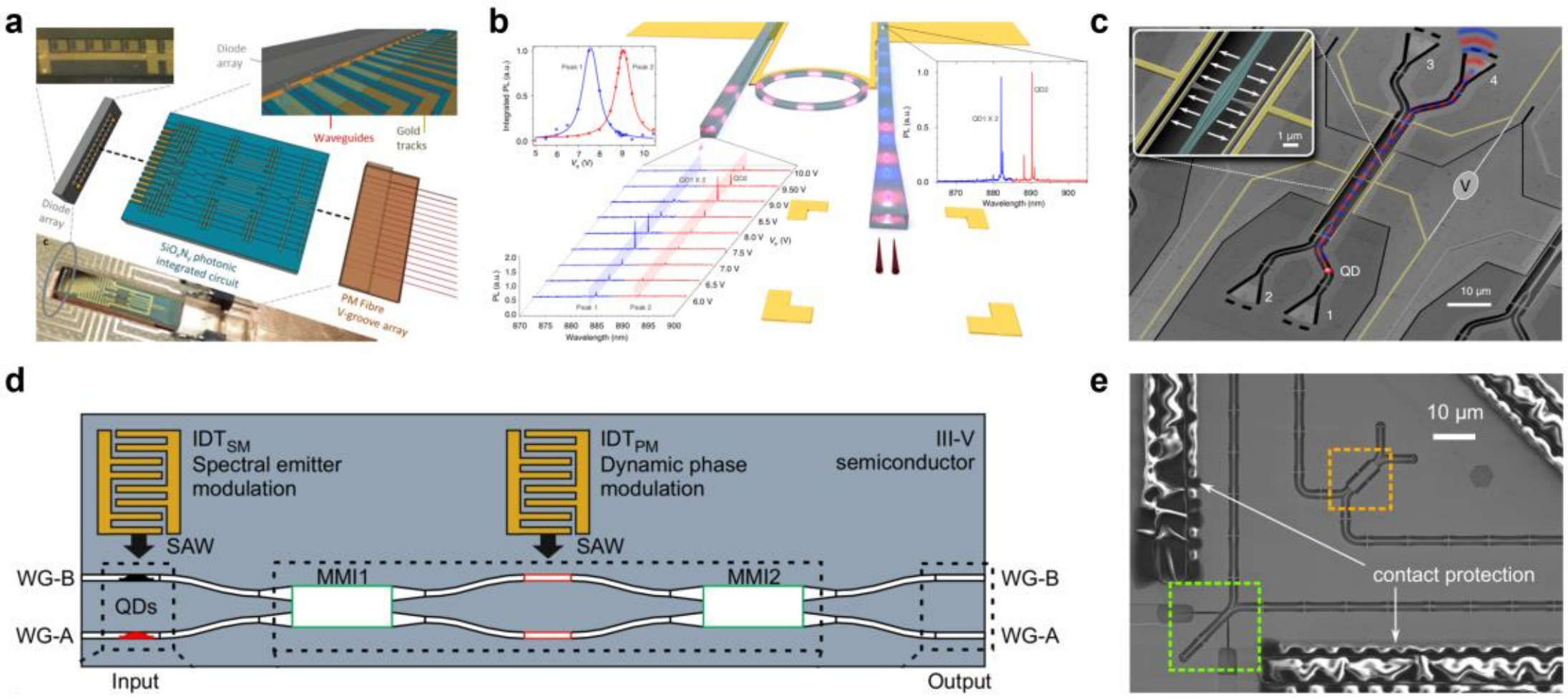

Figure R9. Landscape of integrated deterministic single-photon sources with reconfigurability. (a) Hybrid SiO_xN_y + Thermo-optic[1]: This QPIC features a reconfigurable MZI, with a lithographically defined resistive heater. (b) Hybrid SiN + Thermo-optic[2]: QD source hybrid integrated with SiN ring resonator using thermal heaters to realize wavelength-division multiplexing. (c) Monolithic GaAs + MEMS[3]: Nanomechanical routing of photons. (d) Monolithic GaAs + SAW (Acousto-optic) [4]: Dynamic control using Surface Acoustic Waves. (e) Monolithic GaAs + Electro-optic[5]: Phase shifting via the electro-optic effect in suspended GaAs waveguides.

Table R1: Comparison of modulation mechanisms for integrated deterministic sources.

Modulation Mechanism	Integration Type	Speed / Bandwidth	Heat Load / Cryo-Compatibility
**Thermo-optic**	Hybrid (SiO_xN_y) [1]<br>Hybrid (SiN) [2]	**Slow** (~kHz)	**High (mW dissipation, incompatible with dense integration at 4K)**
**MEMS (Mechanical)**	Monolithic (GaAs) [3]	**Medium** (~10 MHz)	**Low (Capacitive, excellent for cryo)**
**Acousto-optic (SAW)**	Monolithic (GaAs) [4]	**Fast** (~GHz)	**Medium (RF heating, Fixed resonance frequency)**
**Electro-optic (GaAs)**	Monolithic (GaAs) [5]	**Fast** (~GHz)	**Medium/High (Trade-off between propagation loss in doped layers and modulation efficiency)**
**Electro-optic (Pockels) This Work**	Heterogeneous	**Fast** (> GHz)	**Low (Voltage-driven, negligible static power, low loss)**

As shown in Table R1, each platform faces specific trade-offs, which our work aims to resolve:

1) **Thermo-optic Tuning[1,2]:** Thermo-optic phase shifters are the standard for room-temperature photonics but face severe limitations in quantum applications. The milliwatt-level heat dissipation can disturb the cryostat temperature, causing spectral drifting of Quantum Dots (QDs). Furthermore, the slow thermal response (kHz) makes them unsuitable for **feed-forward operations**, which require nanosecond-scale latencies.
2) **MEMS Actuation[3]:** Nanomechanical (MEMS) approaches successfully address the heat issue. However, their switching speed is physically limited by mechanical inertia to the **MHz regime**. This bandwidth is insufficient for the rapid active routing required in modern quantum computing architectures.
3) **Acousto-optic (SAW) [4]:** Surface acoustic wave devices achieve GHz speeds but are inherently

resonant, operating efficiently only at specific RF frequencies (narrowband). This lacks the broadband DC-to-RF flexibility required for arbitrary circuit reconfiguration.

4) **GaAs Electro-optic[5]:** Monolithic GaAs electro-optic modulators offer high speeds. However, efficient modulation often requires doped layers or complex suspended structures, which can introduce additional **propagation loss** or fabrication challenges that compromise the yield of large-scale circuits.

5) **Our Solution (LTOI Pockels Effect):** Our work leverages the LTOI platform to overcome these bottlenecks. By harnessing the Pockels effect in LTOI, we achieve the **GHz-bandwidth** required for fast feed-forward control while maintaining **negligible static power dissipation** and **low optical loss**. This combination, enabled by our heterogeneous integration, provides a robust solution for scalable, active quantum photonic processors.

In the revised **Supplementary Information Note IX**, we have added a comparison of modulation mechanisms for integrated deterministic sources.

1. Ellis, D. J. P. *et al.* Independent indistinguishable quantum light sources on a reconfigurable photonic integrated circuit. *Appl. Phys. Lett.* **112**, 211104 (2018).
2. Elshaari, A. W. *et al.* On-chip single photon filtering and multiplexing in hybrid quantum photonic circuits. *Nat. Commun.* **8**, 379 (2017).
3. Papon, C. *et al.* Nanomechanical single-photon routing. *Optica* **6**, 524 (2019).
4. Bühler, D. D. *et al.* On-chip generation and dynamic piezo-optomechanical rotation of single photons. *Nat. Commun.* **13**, 6998 (2022).
5. Midolo, L. *et al.* Electro-optic routing of photons from a single quantum dot in photonic integrated circuits. *Opt. Express* **25**, 33514 (2017).

- A comparison of the potential of this platform to other quantum photonic processors would be instructive as to the value of this work in the broader quantum hardware community. Currently only a comparison to prior work on very similar photonic platforms is offered. This comparison should show how this work has improved the performance of photonic platforms based on deterministic single photon sources compared to other photonic platforms.

Response:

We sincerely appreciate the reviewer's suggestion to contextualize our work within the broader quantum hardware landscape. We agree that a direct comparison with other quantum photonic processors is essential to articulate the unique value of our hybrid Quantum Dot-Lithium Tantalate (QD-LT) platform. To this end, we provide a detailed comparison table (Table R2) in this response letter to visually summarize these technical distinctions.

To rigorously assess the potential of our platform, we analyzed established architectures based on three critical performance metrics: source determinism, reconfiguration, and detection integration. Our comparative analysis indicates that while existing platforms excel in specific metrics, they face inherent trade-offs that prevent the simultaneous realization of all key requirements for active quantum processing.

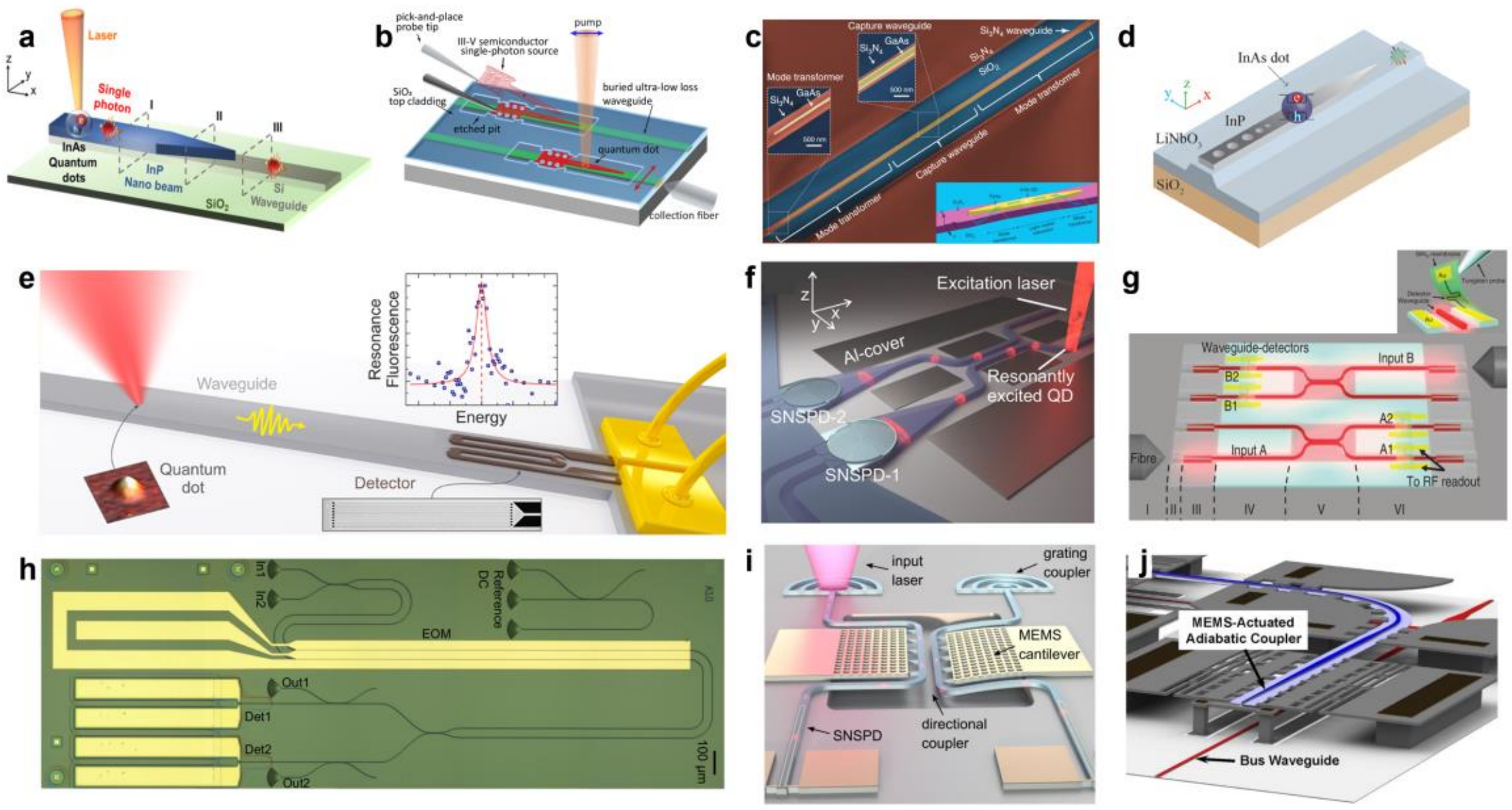

Figure R10. Landscape of state-of-the-art integrated quantum photonic platforms. (a–d) Heterogeneous Integration (Source-focused) [6–8,15]**:** These works achieve high-quality source integration but lacking active, high-speed single-photon modulator. **(e–f) Monolithic III-V Platforms Integration**[9,10]**:** These devices demonstrate integration high-purity single-photon generation and efficient in-situ detection. **(g–j) Scalable Routing & Detection (Speed/Scale-focused)**[11–14]**:** These platforms demonstrate large-scale switching or high-speed LNOI modulation. However, they either lack integrated deterministic sources or are limited by MEMS response times.

Table R2: Comparison of this work with state-of-the-art quantum photonic processors.

Platform Category	Representative Works	Integrated Deterministic Source?	Modulation Mechanism & Speed	Integrated Detection?	Critical Limitation for Active Quantum Processing
**I. Heterogeneous Integration (Source-focused)**	Chanana et al. (2022)[7]	Yes	No	No	**Lacking high-speed single-photon modulators.**
**II. Monolithic III-V**	Schwartz et al. (2018)[9]	Yes	No	Yes	**Lacking high-speed single-photon modulators.**
**III. MEMS**	Seok et al. (2016)[11] Gyger et al. (2021)[12]	No	MEMS (MHz / kHz)	Yes	**Bandwidth Bottleneck: Response time ($\sim\mu s$) exceeds photon lifetime.**
**IV. High-Speed LNOI**	Lomonte et al. (2021)[13]	No	Electro-optic (> GHz)	Yes	**Missing Source: Relies on external inputs, limiting scalability.**
**This Work**	(Our Manuscript)	Yes	Electro-optic (> 40 GHz)	Compatible	**Lacking detection.**

1) The first category encompasses heterogeneous integration efforts that have achieved the crucial

step of combining high-quality III-V quantum dots with low-loss waveguides (such as Si3N4 or Si)[6–8]. However, these platforms have not yet integrated high-speed single-photon modulators into their demonstrated on-chip devices. Instead, they typically rely on thermo-optic phase shifters for tuning. This approach is limited by inherent thermal inertia, which restricts modulation speeds to the kHz range. Furthermore, the associated heat dissipation poses a significant challenge to maintaining the strict cryogenic stability required for quantum dot operation.

2) The second category comprises **monolithic III-V platforms**, which successfully integrate sources, waveguides, and detectors (SNSPDs) on a single chip[9,10]. However, achieving low-loss, high-speed electro-optic modulation on GaAs is fundamentally challenging; it often requires doping that introduces free-carrier absorption, creating a trade-off between propagation loss and modulation speed that limits the scale of the processor.
3) The third category includes **large-scale reconfigurable silicon/MEMS circuits**. While *Seok et al.* demonstrated impressive scalability with 64×64 switch matrices[11] and *Gyger et al.* solved the cryogenic compatibility issue using MEMS[12], both approaches face bandwidth limitations. MEMS actuators are restricted by mechanical inertia to MHz speeds.
4) The fourth category represents **high-speed LNOI platforms**, which offer GHz-bandwidth modulation and detection but lack integrated deterministic sources[13,14]. Reliance on external probabilistic sources (e.g., SPDC) or off-chip lasers introduces significant coupling losses and hinders the scaling of multi-photon logic.

In contrast, the hybrid LTOI-QD platform presented here uniquely bridges the gap between "Deterministic Generation" and "Ultra-fast Routing." By heterogeneously integrating InAs QDs with thin-film Lithium Tantalate, we combine the deterministic photon emission of the former with the superior Pockels effect of the latter. As demonstrated in our work, this allows for modulation bandwidths exceeding 40 GHz—orders of magnitude faster than MEMS or thermal approaches—without the propagation losses associated with doped GaAs or the coupling losses of external sources. This performance improvement is critical because it unlocks the potential for active quantum feed-forward (measuring a qubit and reconfiguring the circuit within the photon's lifetime), a capability that is indispensable for future fault-tolerant quantum computing.

In the revised **Supplementary Information Note X**, we have added a comparison of this work with state-of-the-art quantum photonic processors.

6. Kim, J.-H. *et al.* Hybrid integration of solid-state quantum emitters on a silicon photonic chip. *Nano Lett.* **17**, 7394–7400 (2017).
7. Chanana, A. *et al.* Ultra-low loss quantum photonic circuits integrated with single quantum emitters. *Nat. Commun.* **13**, 7693 (2022).
8. Davanco, M. *et al.* Heterogeneous integration for on-chip quantum photonic circuits with single quantum dot devices. *Nat. Commun.* **8**, 889 (2017).
9. Schwartz, M. *et al.* Fully on-chip single-photon Hanbury-Brown and Twiss experiment on a monolithic semiconductor–superconductor platform. *Nano Lett.* **18**, 6892–6897 (2018).
10. Reithmaier, G. *et al.* On-chip generation, routing, and detection of resonance fluorescence. *Nano Lett.* **15**, 5208–5213 (2015).
11. Seok, T. J., Quack, N., Han, S., Muller, R. S. & Wu, M. C. Large-scale broadband digital silicon

photonic switches with vertical adiabatic couplers. *Optica* **3**, 64 (2016).
12. Gyger, S. *et al.* Reconfigurable photonics with on-chip single-photon detectors. *Nat. Commun.* **12**, 1408 (2021).
13. Lomonte, E. *et al.* Single-photon detection and cryogenic reconfigurability in lithium niobate nanophotonic circuits. *Nat. Commun.* **12**, 6847 (2021).
14. Najafi, F. *et al.* On-chip detection of non-classical light by scalable integration of single-photon detectors. *Nat. Commun.* **6**, 5873 (2015).
15. Aghaeimeibodi, S. *et al.* Integration of quantum dots with lithium niobate photonics. *Appl. Phys. Lett.* **113**, 221102 (2018).

- What is the yield of the transfer process, and are there limitations on the maximum possible yield? Is this fabrication methodology scalable in terms of both yield and volume? In the supplementary it is mentioned that three GaAs waveguides are simultaneously transferred, and yet there is only data shown for one of these. Do the other two not work, or not work as well? This information should be included for readers to effectively evaluate the significance of this result.

Response:

We sincerely thank the reviewer for raising these important questions regarding the yield, scalability, and clarity of our transferred samples. These are indeed crucial metrics for evaluating the practical significance of our hybrid integration approach. We have addressed each of your concerns in detail below.

1) Yield and Scalability of the Transfer Process

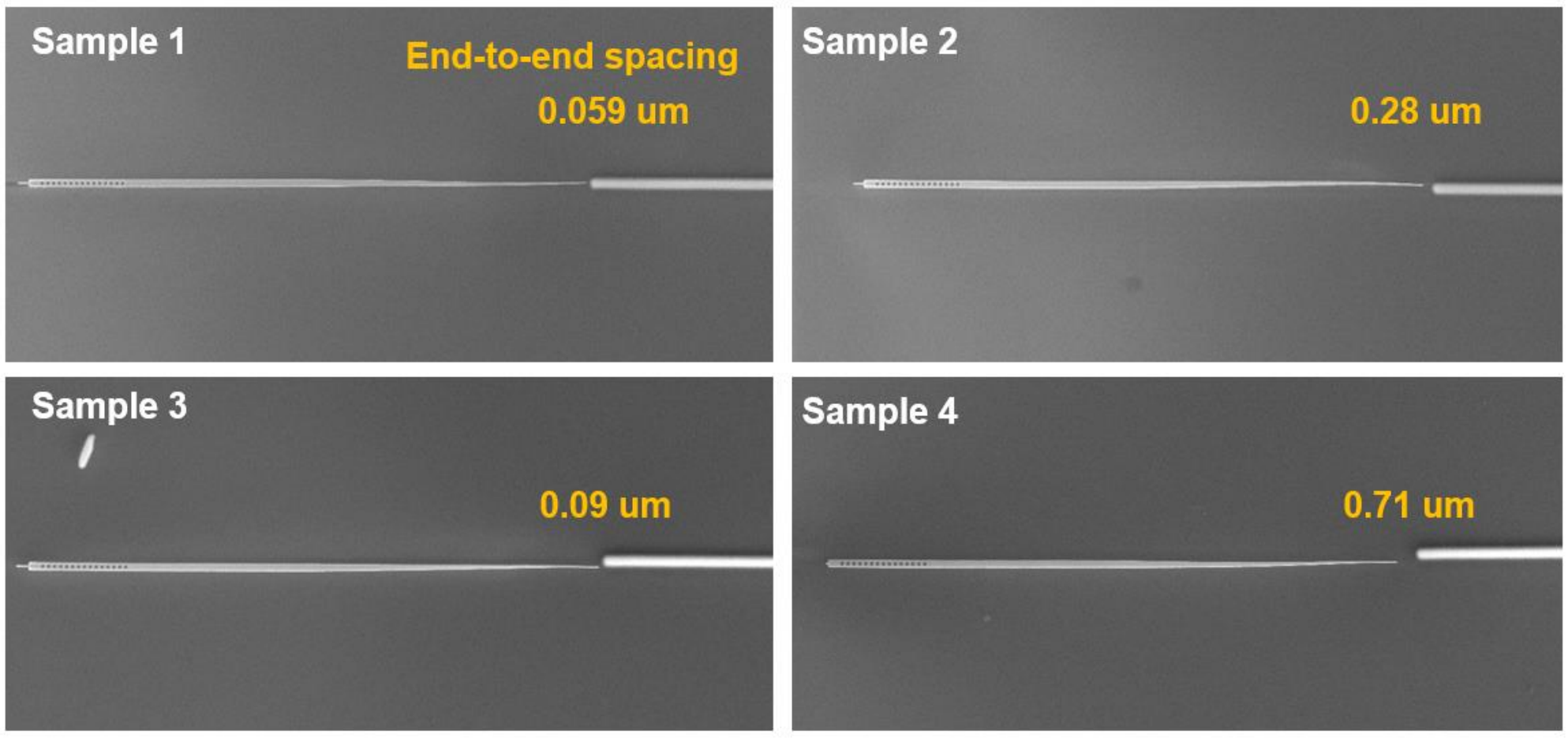

Figure R11. Four of the samples transferred using the high-precision micro-PDMS transfer printing method. The numbers labeled above indicate the end-to-end spacing.

In this proof-of-concept study, our focus was on demonstrating the physical mechanism and single-device performance of the hybrid integration between Quantum Dot sources and the Lithium Tantalate (LT) platform. Therefore, the transfers were performed on a single-sample basis in a laboratory setting, and we did not focus on optimizing for mass-production yield. However, our manual process consistently achieved the required alignment precision for successful coupling. To better illustrate the reliability of our current technique, we have added additional SEM images of

other transferred samples (Fig. R11) to demonstrate that our transfer technology can repeatedly achieve high alignment accuracy.

Regarding the fundamental limitations and maximum possible yield, the methodology we employed (Micro-Transfer Printing) is intrinsically highly scalable in terms of both yield and volume. While our lab-scale setup is geared towards prototyping, state-of-the-art commercial wafer-scale MTP systems (e.g., from ASM AMICRA) have already achieved massively parallel, automated integrations. These commercial tools can routinely achieve sub-micron alignment accuracies of ±300 nm to ±500 nm (3σ) [D. Gomez et al., "Micro Transfer Printing for Micro Assembly of Heterogeneous Integrated Compound Semiconductor Components," CS MANTECH Conference, May 9-12, 2022.], which is well within the tolerance required for our hybrid waveguide architectures. Thus, transitioning this approach from lab-scale single transfers to high-yield, high-volume wafer-level production is completely feasible utilizing existing industry-standard equipment.

2) Clarification on the Three Simultaneously Transferred GaAs Waveguides

We apologize for the confusion regarding the three transferred waveguides mentioned in the Supplementary Information. We want to clarify that the other two GaAs waveguides are structurally intact and fully functional; the reason we only presented data for one is purely due to the geometric design of our target chip.

During the transfer process, the PDMS stamp was large enough to pick up three closely spaced GaAs waveguides from the dense source array simultaneously. However, the spacing (pitch) of the pre-fabricated LT waveguides on the target chip did not match the spacing of the GaAs waveguides. Consequently, only one GaAs waveguide could be aligned and coupled to the target LT waveguide for routing. The other two GaAs waveguides were transferred simply as a byproduct; they landed on the unpatterned area of the LT chip and lacked a corresponding target structure to couple light into. If the target LT waveguides were designed with a matching pitch, all three could operate simultaneously. We have clarified this point explicitly in the revised Supplementary Information to prevent any misunderstanding by future readers.

Supplementary Information Revision:

"As shown in the first red frame, three GaAs waveguides were simultaneously transferred by the stamp. Due to a pitch mismatch between the source array and the target LT chip, only one waveguide was coupled to the LT circuit, while the other two landed intact on unpatterned regions and remain fully functional. This simultaneous transfer demonstrates that, with optimized layout designs, our approach is highly scalable and capable of parallel, large-scale integration."

[Comment 3]

3. Does the work support the conclusions and claims, or is additional evidence needed?

The data supports the conclusions and claims made about the work undertaken here, however some of the broader implications claimed seem to be far-fetched.

- In the abstract the authors state: "The scalability of quantum photonic processors relies on simultaneously integrating deterministic single-photon sources, low loss photonic circuitry and fast reconfigurability." This is quite a strong statement considering some of the largest photonic quantum computing companies, (e.g. PSIQuantum, Xanadu) do not follow this approach. I also do not think the contents of this paper supports this conclusion. I would recommend including an analysis of this

statement in the body of the paper, providing references to prove other photonic quantum computing approaches infeasible, and to prove this approach as the only suitable one.

Response:

We thank the reviewer for this critical and valuable observation. We agree that our original statement ("relies on") was too absolute, as it inadvertently dismissed other successful approaches such as those based on probabilistic heralded sources (e.g., PsiQuantum) or continuous-variable squeezed states (e.g., Xanadu).

In the revised manuscript, we have softened this statement in the abstract to present our approach as "a promising pathway" rather than the only solution. We clarify that the specific advantage of our hybrid architecture lies in the deterministic nature of QDs, which can significantly reduce the resource overhead associated with multiplexing probabilistic sources.

Furthermore, to concretely support the scalability of our platform, we have added a comprehensive discussion in the Discussion section. We explicitly analyze how integrating QDs with fast modulators enables scalable functionalities, such as active demultiplexing (for generating multi-photon states) and fusion measurements (for cluster state generation).

Nowadays, some leading commercial entities such as **Quandela** and **Sparrow Quantum**, whose recent achievements demonstrate that deterministic QD sources are transitioning from academic research to robust engines for quantum computing. Quandela has successfully commercialized the MosaiQ system, which they distinguish as "the first datacenter-ready photonic quantum computer." This system integrates their "eDelight" sources—InAs quantum dots deterministically coupled to micropillars—which operate at 80 MHz clock rates with indistinguishability >95% and purity $g^{(2)}(0)< 0.01$. The deployment of such rack-mounted, cloud-accessible systems in standard data centers serves as a definitive proof-of-concept for the scalability of discrete-variable quantum computing. [Quandela. MosaiQ: The first full-stack photonic quantum computer. *Quandela Official Website* https://www.quandela.com/products/mosaiq (2025).]

Similarly, Sparrow Quantum's "Sparrow Core" leverages a planar nanophotonic waveguide design to effectively filter pump light while maximizing collection. This architecture delivers near-unity waveguide collection efficiency (β> 95%), exceptional single-photon purity ($g^{(2)}(0) \approx 0.01$), and indistinguishability >97%. Such specifications provide a "plug-and-play" deterministic resource that eliminates the need for the massive spatiotemporal multiplexing networks required by parametric down-conversion (SPDC) schemes. [Sparrow Quantum. Sparrow Core: Deterministic single-photon source specifications. *Sparrow Quantum Technology* https://sparrowquantum.com/technology (2025).]

Manuscript Revision:

" A promising pathway towards scalable quantum photonic processors involves the simultaneous integration of deterministic single-photon sources, low-loss photonic circuitry, and fast reconfigurability."

- The authors offer a list of advantages of lithium tantalate over lithium niobate, but it is not clear to me how these properties are an advantage for building a quantum photonic processor. Specifically how do an expanded transparency window, and decreased optical anisotropy help for this endeavour?

Response:

We appreciate the reviewer for this insightful critique. This is also mentioned by reviewer 2. We agree with that since our InAs QDs emit at ~900 nm, the extended UV transparency window of LTOI (down to 0.28 μm) is indeed not a critical advantage for this specific work compared to LNOI. Therefore, in the revised manuscript, we have removed the emphasis on comparing LTOI against LNOI regarding these aspects. Instead, we now highlight the general suitability of ferroelectric thin-film platforms (including both LTOI and LNOI) for cryogenic quantum photonics, focusing on their shared benefits such as low propagation loss, high electro-optic coefficients, and strong optical confinement.

Manuscript Revision:

"Moreover, ferroelectric thin-film platforms like LT offer exceptional properties for integrated quantum photonics, including stable electro-optic response at cryogenic temperatures and low optical propagation losses at the emission wavelength of the quantum emitters (~900 nm)[11–14]. These characteristics are crucial for building low-loss and reconfigurable photonic integrated circuits that operate effectively with on-chip quantum emitters."

11. Wang, C. *et al.* Lithium tantalate photonic integrated circuits for volume manufacturing. *Nature* **629**, 784–790 (2024).
12. Yu, J. *et al.* Tunable and stable micro-ring resonator based on thin-film lithium tantalate. *APL Photonics* **9**, 036115 (2024).
13. Wang, H. *et al.* Thin-film lithium tantalate modulator operating at high optical power. *ACS Photonics* acsphotonics.5c00159 (2025).
14. Huang, F. *et al.* Toward large - scale photonic chips using low - anisotropy thin - film lithium - tantalate. *Adv. Sci.* **12**, 2410345 (2025).

- The electro-optic modulators demonstrated here have a large footprint (>3mm long). How does the size of this platform affect its integration density potential, and its utility for small and large-scale quantum photonic processors, especially concerning cryogenic cooling space requirements? I think some numbers are necessary to provide a convincing claim that this platform is useful for quantum photonic processors.

Response:

We thank the reviewer for raising this practical concern. We would like to clarify the integration potential from two aspects: the device aspect ratio and future miniaturization.

1) High Aspect Ratio Packing: While the interaction length is >3 mm to ensure low driving voltage and adiabatic transitions, the lateral footprint of each device is extremely compact. As shown in Figure R12, the device width is only 0.34 mm. This high aspect ratio allows for dense parallel integration. Even on a standard 10mm×10mm chip carrier used in cryostats, we can accommodate an array of >25 independent modulators side-by-side. This density is already sufficient for many intermediate-scale quantum processing tasks, such as multi-mode Boson sampling.
2) Path to Miniaturization: Furthermore, the high refractive index contrast of LTOI allows for

folded waveguide architectures [*Opt. Express 30, 9193-9207 (2022)*]. Future designs can fold the 3 mm length into a compact spiral or meander structure *[Opt. Express 30, 40666-40681 (2022), Opt. Express 30, 40666-40681 (2022)*], reducing the footprint to <1mm^2, further increasing the integration density by an order of magnitude.

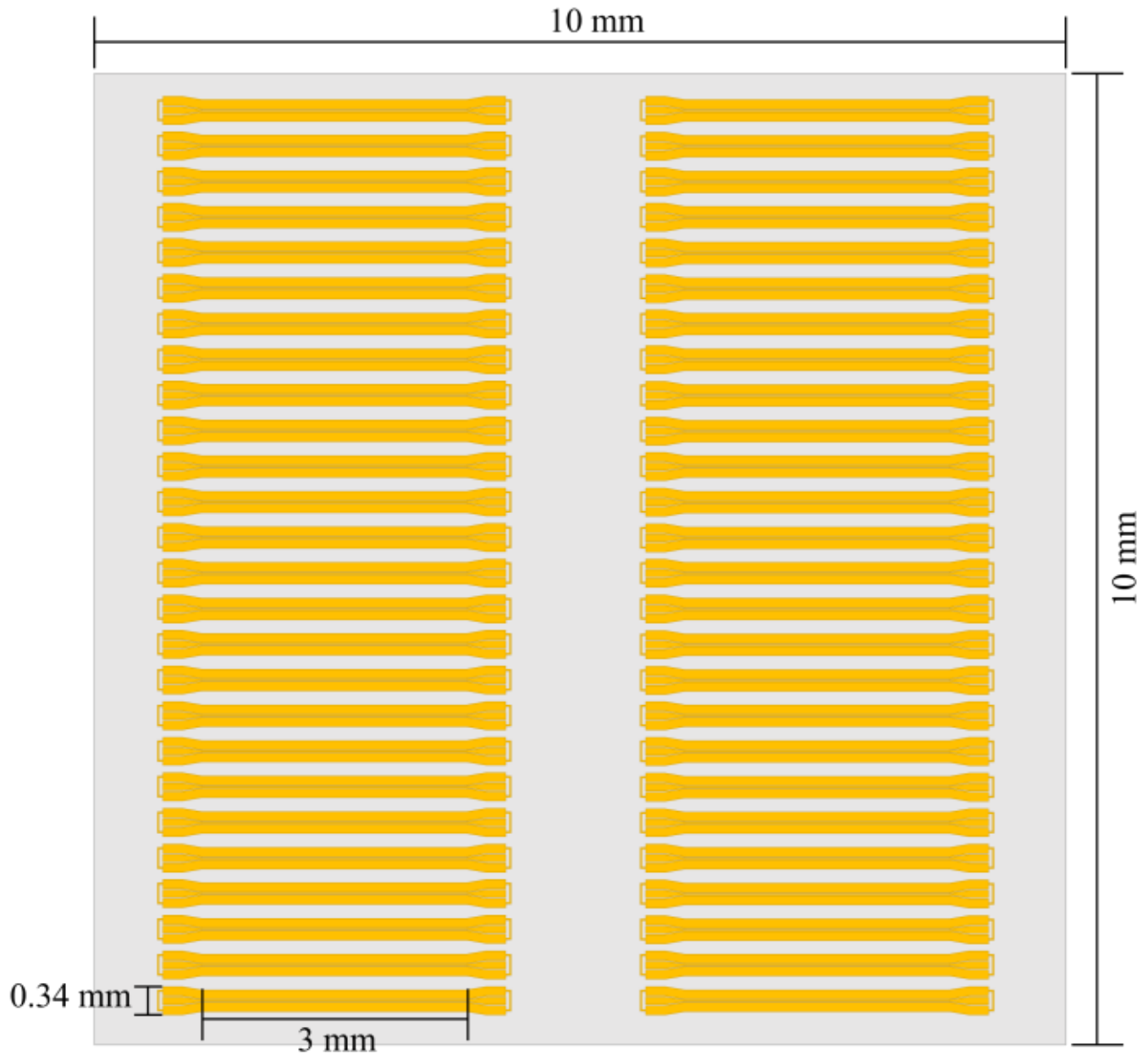

Figure R12. Conceptual layout of a parallel modulator array on a standard 10 mm×10 mm cryogenic chip carrier. Due to the high aspect ratio of the devices, a single chip can accommodate >25 independent channels side-by-side.

[Comment 4]

4. Are there any flaws in the data analysis, interpretation and conclusions? - Do these prohibit publication or require revision?

I found no flaws in the data analysis that would prevent publication, however there are some things that should be more precisely stated.

- The estimation of detection efficiency does not contain any error margins in the transmission of various optical components. While this would normally be immaterial, when the total system transmission is so poor 1%, these uncertainties may make a significant difference to the uncertainty in the final extraction efficiency. This final extraction efficiency should be quoted with an uncertainty.

Response:

We sincerely thank the reviewer for this rigorous and highly constructive comment. Minute uncertainties in the transmission of individual optical components can indeed significantly impact the calculation of the final extraction efficiency due to error propagation. We have now conducted a comprehensive evaluation of the transmission uncertainties across the entire optical path. In the revised **Supplementary Information Note VII**, we have detailed the transmission uncertainty for each optical element based on reasonable estimates derived from the official datasheets provided by the manufacturers.

a

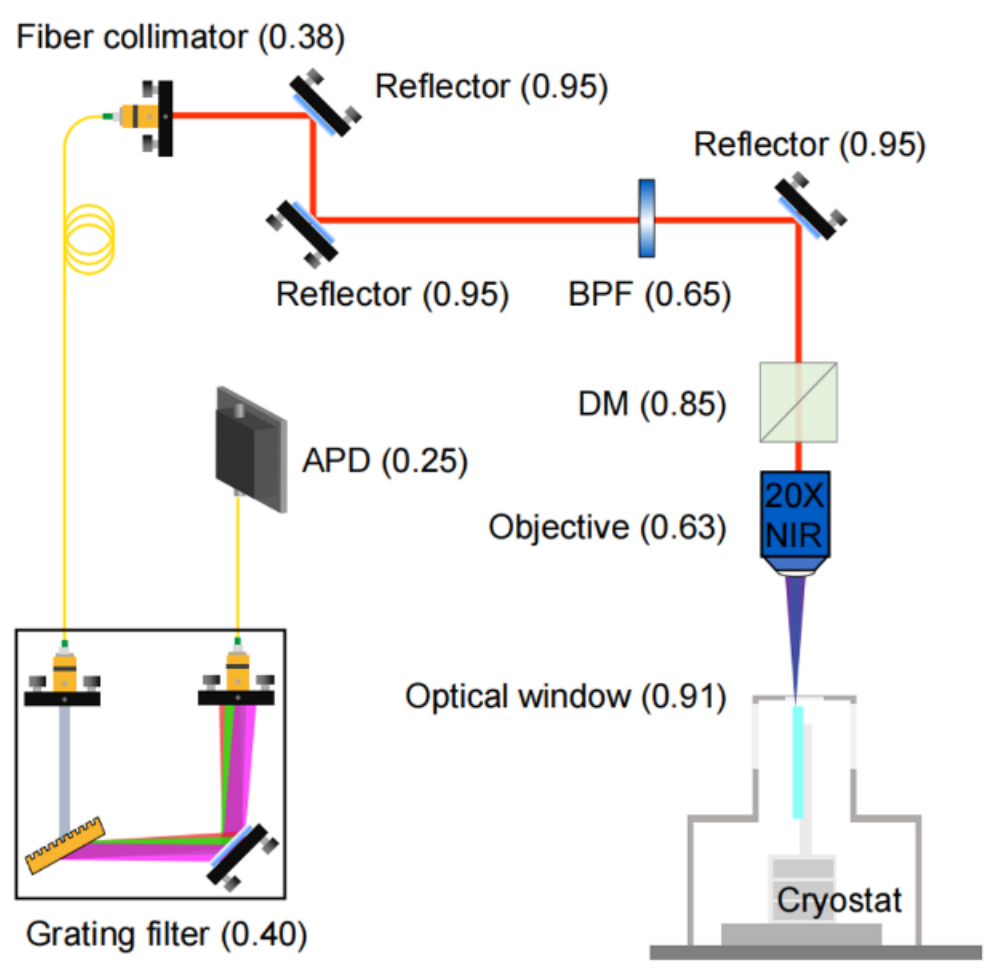

b

Optical setup	Transmission
Optical window	0.91±0.01
Objective	0.63±0.03
DM	0.85±0.02
BPF	0.65±0.02
Reflector×3	$(0.95\pm0.01)^3$
Fiber collimator	0.38±0.05
Grating filter	0.40±0.10
APD	0.25±0.02
Total setup	0.0103±0.0012

Figure R13. Calibration of optical transmission losses of the optic elements in the setup. (a) All the optical elements used in the collection path. (b) Transmissions of the optical elements in the setup with a total efficiency of 0.0103±0.0012.

The total transmission efficiency of the setup was measured at 0.0103±0.0012. Incorporating these error margins, we have re-evaluated the final single-photon extraction efficiency at the first objective lens. By employing an 80 MHz femtosecond pulsed laser for above-band excitation of the quantum dot, the saturated count rate of 0.245 ± 0.005 MHz was extracted from the power-dependent photoluminescence saturation curve in Fig. 4b. This yields a measured single-photon extraction efficiency at the first objective lens of $\eta_{exp} = \frac{0.245\pm0.005}{(0.0103\pm0.0012)\times80} = 0.297 \pm 0.035$.

Furthermore, we would like to clarify that the current extremely low total efficiency is primarily attributed to the inherently low detection efficiency (~25%) of the Avalanche Photodiode (APD) used in our experiments. In future work, upgrading the detection setup to a Superconducting Nanowire Single-Photon Detector (SNSPD, which typically offers a detection efficiency >75%) will multiply the total system efficiency.

- A plot of the experimental output mode profile that was measured to have an overlap of 0.39 with the objective would also be a good visual support to the discussion about system collection efficiency. A discussion on the use of tapers or gratings to improve this coupling efficiency, and how far it could be improved could increase the impact by allowing readers to recognise the potential performance improvements available in this system.

Response:

We greatly appreciate the reviewer's suggestion to provide visual support for the coupling efficiency analysis and to discuss the potential for future optimization. In the revised **Supplementary Information Note VII**, we have added Figure R14, which presents the FDTD-simulated far-field emission profile of the LTOI waveguide and its overlap with the collection optics.

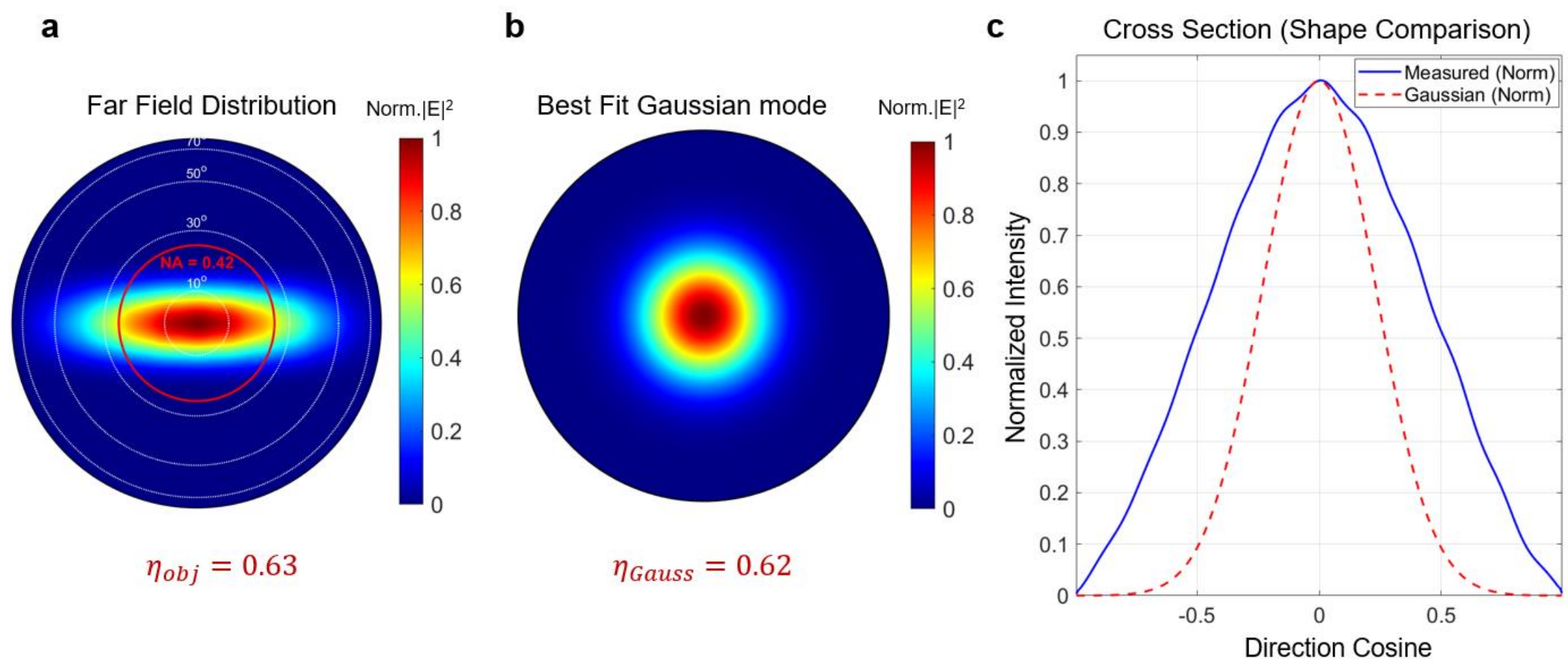

Figure R14. Far-field emission characteristics and fiber coupling analysis. (a) Simulated far-field radiation intensity distribution ($|E|^2$) emitted from the LTOI waveguide facet. The red dashed circle delineates the acceptance cone of the objective lens (NA=0.42), which collects approximately 63% of the total radiated power. (b) The fundamental Gaussian mode profile that provides the maximum overlap with the emitted far-field pattern. The calculated overlap integral yields a mode matching efficiency of $\eta_{Gauss} \approx 0.62$. (c) Comparison of the cross-sectional intensity profiles between the waveguide far-field emission (solid line) and the fitted Gaussian mode (dashed line). The deviation between the two curves visually illustrates the mode mismatch responsible for energy loss.

To clarify the "0.39" overlap factor, our analysis reveals it is the product of two physical constraints: the geometric collection efficiency of the objective lens ($\eta_{obj} \approx 0.63$) and the fiber mode overlap ($\eta_{Gauss} \approx 0.62$). Thus, 0.39 corresponds to the product of lens collection and fiber matching ($\eta_{obj} \times \eta_{Gauss} \approx 0.63 \times 0.62 \approx 0.39$).
As visualized in **Figure R14**, the strong optical confinement in the sub-wavelength LTOI ridge waveguide results in a highly divergent far-field pattern that matches poorly with the fundamental Gaussian mode of the fiber collimator. While the current device employs a simple cleaved facet for proof-of-concept demonstration, we acknowledge that this coupling efficiency is far from the theoretical limit of the material platform.

[Comment 5]
5. Is the methodology sound? Does the work meet the expected standards in your field?
The methodology is sound and meets expected standards for original research of moderate importance in this field.
- How are differences in coupling efficiency accounted for during the measurement of propagation loss through the cutback method. The standard method for propagation loss is to measure the quality factor of a resonant structure. Why is this non-standard method used?

Response:
We thank the reviewer for raising this important point regarding the measurement methodology. Regarding the choice of method, while the quality factor measurement using resonant structures is indeed a standard approach, it was not suitable for our specific experimental setup. We attempted to

characterize micro-ring resonators; however, due to the resolution limitations of our spectrometer in the 900 nm band, the measured resonance dips exhibited low extinction ratios. This made it difficult to accurately determine the full width at half maximum (FWHM) and extract the intrinsic Q-factor. Consequently, we adopted the cut-back method, which provided a more direct and reliable estimation of the propagation loss for our specific waveguide geometry and wavelength.

To address the variations in coupling efficiency during the cut-back measurements, we implemented a rigorous alignment protocol. For each waveguide of varying length, we meticulously optimized the fiber-to-chip alignment to maximize the transmission power, thereby ensuring that the coupling efficiency was consistent and optimal across all measurements. Furthermore, the propagation loss was extracted from the slope of the linear fit of transmission versus length, which averages out residual random fluctuations in coupling efficiency.

- The performance of the modulation should be reported using with common measurements for modulation performance, including insertion loss, 3dB modulation bandwidth, and Extinction ratio in dB.

Response:

We appreciate the reviewer's suggestion to adhere to standard performance metrics. We would like to clarify that these standard metrics are indeed fully characterized and presented in our manuscript:

1) Insertion Loss: As analyzed in the manuscript, the device exhibits a total fiber-to-fiber insertion loss of 8.5 dB. By calibrating out the edge coupling losses (using reference waveguides), we determined the intrinsic on-chip insertion loss to be approximately 0.5 dB, demonstrating the high quality of the LTOI fabrication.
2) 3 dB Modulation Bandwidth: The high-speed electro-optic response is detailed in Figure 2c, demonstrating a 3 dB bandwidth exceeding 40 GHz.
3) Extinction Ratio: The DC characterization presented in Figure 2d confirms a static extinction ratio exceeding 20 dB.

- Why are all of the transmission values for optical components so low? Is nothing in this setup anti-reflection coated, as would be standard?

Response:

We sincerely thank the reviewer for pointing this out. We would like to clarify that the transmission values are not theoretical estimates, but rather in-situ experimental data carefully calibrated using a narrow-linewidth continuous-wave laser at the exact emission wavelength of the quantum dots. Regarding the anti-reflection (AR) coatings, we utilized standard off-the-shelf optical components. While they possess generic broadband coatings, they were not custom-tailored or optimized for the specific emission wavelength of our quantum dots. Furthermore, the overall low efficiency stems from several practical constraints in our current lab-scale characterization setup, rather than fundamental limitations of the integrated chip itself:

1) **Facet Divergence and NA Limits:** Due to the strong optical confinement within the Lithium Tantalate (LT) ridge waveguide, the outcoupled beam diverges significantly at the unoptimized facet. Our objective lens ($NA = 0.42$) has a limited collection angle, which heavily restricts the collection efficiency.

2) **Bandpass Filter (BPF) Tilt Loss:** The center wavelength of our available BPF did not perfectly match the specific QD emission wavelength. To actively tune the passband to the target wavelength, the filter had to be physically tilted. This angle-tuning inevitably introduces substantial insertion losses and beam displacement.
3) **Grating Filter Losses:** The grating filter unit intrinsically introduces severe losses. This is due to the combination of the diffraction efficiency of the grating itself and the double fiber-coupling interfaces (from free-space to fiber, and vice versa) required in the spectrometer path.
4) **Detector Bottleneck (APD):** The most significant bottleneck in our total system efficiency is the single-photon detector. We currently employ an Avalanche Photodiode (APD) with a detection efficiency of only ~**25%** at this wavelength.

There would be a large room for optimization if the optical path were upgraded with custom AR-coated optics specifically designed for the QD wavelength, integrated with facet mode expander to improve fiber coupling, and measured using a Superconducting Nanowire Single-Photon Detector (SNSPD, which typically offers >75% detection efficiency), the total system efficiency would improve by orders of magnitude.

[Comment 6]

6. Is there enough detail provided in the methods for the work to be reproduced?

There is enough detail in the methods to reproduce this work with the following exception:

- A reference, or more detail, is necessary to be able to reproduce the GaAs/InAs quantum dot waveguide fabrication method.

Response:

We thank the reviewer for this valuable comment, which has helped us improve the completeness of the manuscript. Regarding the fabrication of the GaAs/InAs quantum dot waveguide, the following detailed process flow has been added to the **Supplementary Information Note II**:

The waveguide was processed from a wafer with embedded InAs/GaAs quantum dots grown by molecular-beam epitaxy. First, an ARP resist was spin-coated and patterned via electron-beam lithography to form a mask. Subsequently, a 200-nm-thick GaAs layer was etched through by inductively coupled plasma etching. After removal of the residual resist, the sample was immersed in a 2 % HF solution to wet-etch the AlGaAs sacrificial layer, ultimately yielding a suspended GaAs/InAs quantum dot waveguide. A scanning electron microscopy (SEM) image of the fabricated waveguide is presented in Fig. R15(b).

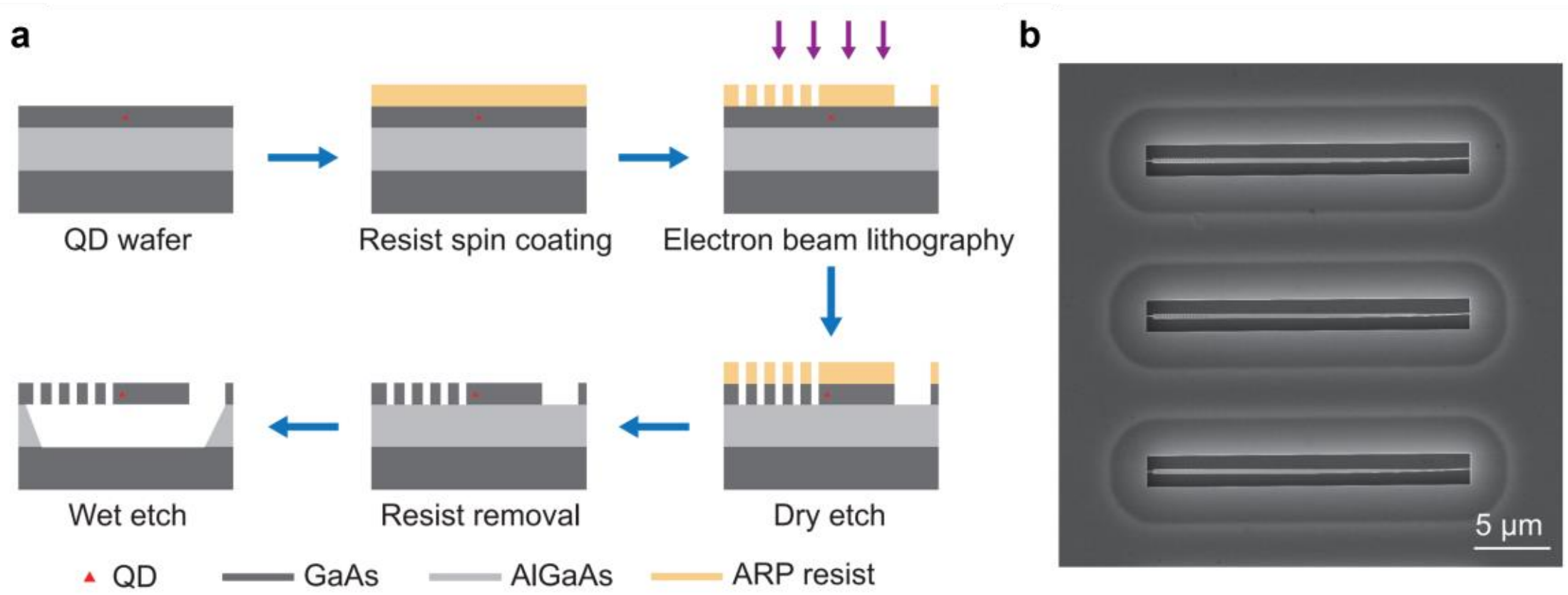

Figure R15. Fabrication of the suspended GaAs/InAs quantum dot waveguide. (a) Schematic of the fabrication process flow. (b) SEM image of the fabricated waveguide.

[Comment 7]

Journal-specific Guidelines:

• The quality of the data — whether they are technically sound, obtained with appropriate techniques, analysed and interpreted carefully, and presented in sufficient detail.

• While Fig 1a. is appropriate for a later section discussing the future potential in this platform, I believe a schematic of the actual device under test is much more suitable for inclusion in a figure presenting experimental results. Furthermore without a schematic of the actual device under test it is not so clear what this paper has achieved.

• Fig 1b,c,d seems like a very strange way to represent this data. As there is very little wavelength dependency, why not plot the independent variable vs the dependent variable, and represent the wavelength as a series. This would make the plots clearer. Additionally it would be instructive to include the experimentally measured values of spacing, lateral offset, and angle offset for the three placed GaAs waveguides either in the text or on these plots, to demonstrate what values were achieved.

Other Comments:

The labelling in fig 1 seems odd with fig f coming before fig e.

Response:

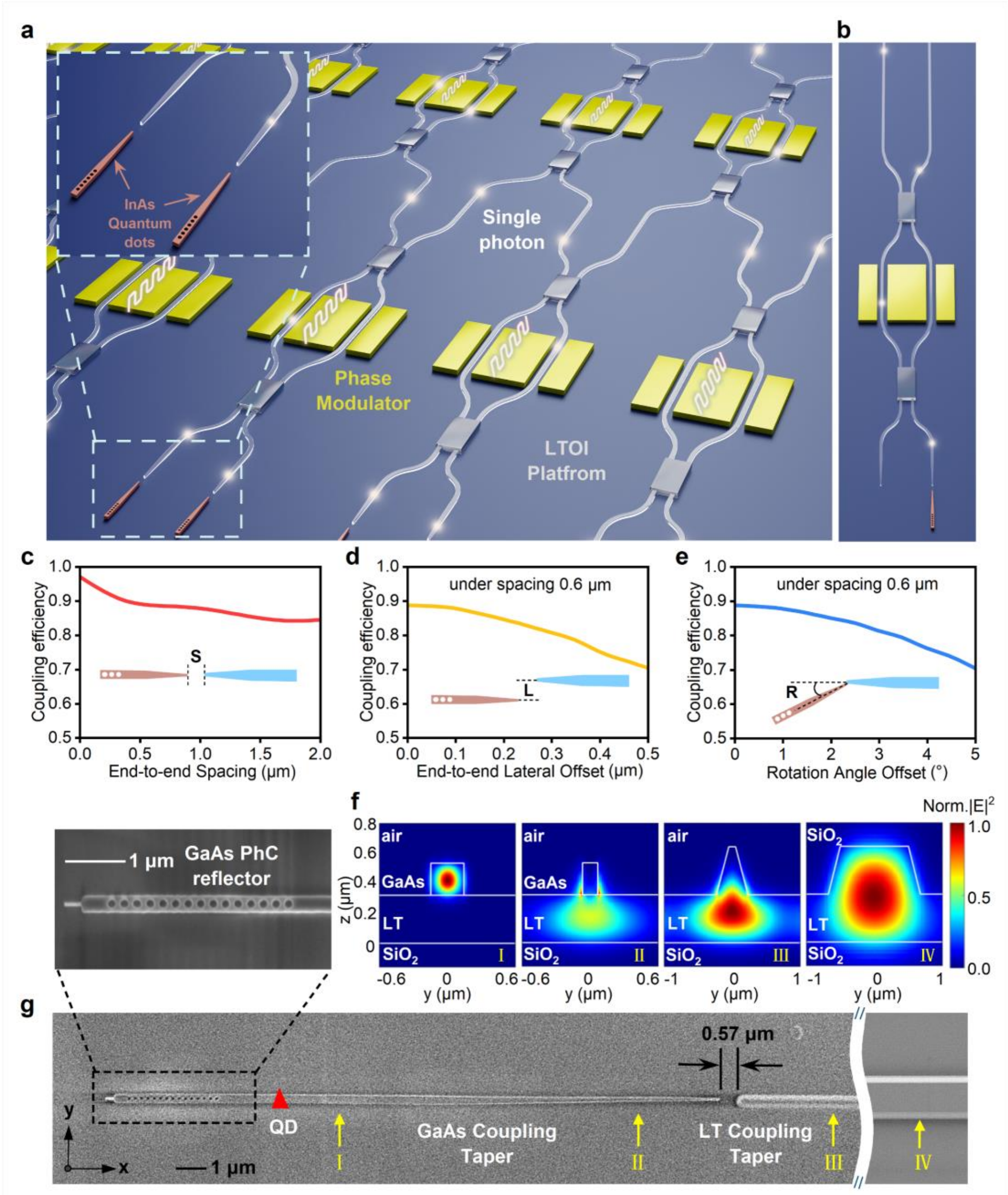

Figure R16. Hybrid quantum photonic device architecture. (a) Artistic schematic of the heterogeneous integration between indium arsenide (InAs) QDs and LT Mach-Zehnder interferometer (MZI). The inset shows a zoomed-in view of the butt-coupled tapered gallium arsenide (GaAs) and LT waveguides. (b) Schematic of the device configuration investigated in this work, comprising a single GaAs waveguide coupled to a single LT MZI. (c-e) Simulated coupling efficiency as a function of (c) end-to-end waveguide spacing, (d) lateral offset, and (e) relative angular misalignment. (f) Simulated cross-sectional electric field distributions at sequential planes (I to IV), illustrating the adiabatic mode transfer from the GaAs waveguide to the LTOI waveguide. Cross-section IV indicates the untapered LT waveguide. (g) False-colored scanning electron micrograph of the fabricated hybrid device: GaAs waveguide is aligned to the LT waveguide with sub-100 nm precision. The inset shows a photonic crystal reflector at the end of the GaAs waveguide.

We appreciate the reviewer's constructive suggestion. We have retained the original Fig. 1a (future platform architecture) and added some discussion to the main text to better reflect its role. And we have added a new Figure R16(b), which provides a schematic of the actual device under test. This schematic illustrates the specific heterogeneous integration in our experiments.
We have completely revised these plots (Figures R16(c), R16(d), and R16(e)). The independent variables—end-to-end waveguide spacing, lateral offset, and relative angular misalignment—are now plotted on the x-axis, while the coupling efficiency is on the y-axis.
We apologize for the oversight in the labeling sequence. We have re-labeled all subfigures in Fig. 1 in the correct alphabetical order (a through f) to ensure a logical flow that matches the narrative in the text.

- In figure 4a, the uncertainty of the fitted radiative lifetime should be quoted.
- In figure 4c, the count rate appears to peak, and then reduce with increasing pump power. What is the likely cause of this?

Response:
We have updated Figure 4a and the main text to include the uncertainty for the fitted radiative lifetime. The updated radiative lifetime is quoted as $\tau = 930.0 \pm 0.1$ ps.
We appreciate the reviewer's insightful observation regarding the photoluminescence (PL) saturation curve. The slight reduction in count rate at high pump powers, following the peak, is likely attributed to the thermal effects and decoherence. At high excitation powers, local heating of the sample can occur. This temperature increase enhances phonon-mediated dephasing and decoherence processes, which can reduce the radiative recombination efficiency of the quantum dot.

- The level of support for the conclusions — whether sufficiently strong evidence is provided for the authors' claims and all appropriate controls have been included.

Covered in Editors Q3

- The potential significance of the results — whether these results will be important to the field and advance understanding in a way that will move the field forward. (Note that posting of preprints and/or conference proceedings does not compromise novelty.)
- Covered in Editors Q2
